# Improving governance in the age of synthetic biology, artificial intelligence, and diverging threats

**DOI:** 10.3389/fbioe.2026.1705143

**Published:** 2026-04-20

**Authors:** Dunja M. Sabra, Johannes L. Frieß, Bernd Giese, Gunnar Jeremias

**Affiliations:** 1 Carl Friedrich von Weizsäcker-Center for Science and Peace Research (ZNF), Hamburg University, Hamburg, Germany; 2 Institute of Safety and Risk Sciences, BOKU University, Vienna, Austria

**Keywords:** artificial intelligence (AI), biological weapons (BW), biosecurity, emerging technologies, governance, synthetic biology (SynBio)

## Abstract

**Introduction:**

Advancements in synthetic biology (SynBio) and other emerging and converging technologies, such as artificial intelligence (AI) additive manufacturing (3D printing), and nanotechnology are driving progress at an unprecedented pace. However, these promising and groundbreaking advances could also lead to novel biological risks, including the potential development of SynBio-enabled bioweapons (BW).

**Methods:**

Conducting a Delphi process, we consulted 13 experts from diverse relevant sectors. The multi-stage process included insights from literature reviews, expert interviews, two rounds of expert surveys, and two workshops.

**Results:**

We identified consistent biological threat prioritizations and established consensus-driven policy recommendations. Based on this, we developed a novel hybrid governance framework. Our key proposal includes a multifaceted and integrative approach involving four sequential, iterative components: raising awareness; establishing robust training and monitoring systems to improve biosecurity measures; developing and implementing agile governance frameworks; and strengthening international treaties, such as the Biological Weapons Convention (BWC).

**Conclusion:**

We consider these integral, interconnected components to be interdependent and equally important. In an era of SynBio, AI-driven bioengineering, and democratization of biotechnology, implementing these recommendations will better safeguard against the potential misuse of these advancements in the context of the development and proliferation of BW.

## 1 Introduction

Global health and security are strained by latent crises, such as SARS-CoV-2 (World Health Organization, 2024b), Mpox (World Health Organization, 2024c), H5N1 (Kozlov, 2025) alongside increasingly used hybrid warfare tactics (Mumford and Carlucci, 2023). The latter blends military force, cyber operations (Komninos and Serpanos, 2024), disinformation (Jakob et al., 2022), and economic coercion (Lord Alderdice, 2024; Walker, 2024), blurring civilian-military boundaries in conflicts such as those in Ukraine and Gaza. Additionally, concerns about bioterrorism have also resurfaced (DiEuliis and Giordano, 2022; Jakob et al., 2022; Rezaei, 2025).

Geostrategic rivalries accelerate the fusion of previously separated technologies (Pretorius et al., 2025), creating novel capabilities but also new challenges (Groff-Vindman et al., 2025). This convergence and the rapid technological advances in Synthetic Biology (SynBio) (Mantellass, 2022; Sanz et al., 2022; Sheahan and Wieden, 2021; Undheim, 2024), engineering technologies (Pretorius et al., 2025), information technology (IT) and AI (De Haro, 2024), robotics (Courtney et al., 2023), nanotechnology (Kambouris et al., 2023), and other emerging technologies (Brockmann et al., 2019) are further increasing the complexity of global security by expanding dual-use (DU) risks (Morse, 2024). These rapid, scalable innovations lower barriers for malicious actors, create new vulnerabilities beyond current governance (De Haro, 2024; Mantellass, 2022; Rickli and Vllasi, 2025; Sokhansanj and Rosen, 2025; Undheim, 2024) and amplify attribution and systemic risks (DiEuliis et al., 2024) while complicating detection (Mo et al., 2024), and international response efforts.

### 1.1 Convergence of SynBio with emerging technologies

SynBio emerged in the early 2000s as an interdisciplinary field, combining molecular biology (synthetic) chemistry, IT, and engineering to design and redesign biological components for specific functions (Hanczyc, 2020; Raimbault et al., 2016; Science for Environment Policy, 2016). It aims to create biological systems using reliable, replicable modules, compartments, and even artificial cells or tissues (Benner and Sismour, 2005; Giese et al., 2015; Heinemann and Panke, 2006; Pfeifer et al., 2022; Schwille, 2011). Key goals can be to standardize component libraries for creating novel biological functions (Abramson and Levin, 2021; Blackiston et al., 2023), synthesizing extensive DNA segments, and exploring artificial biochemical systems (Hirschi et al., 2022; Jia et al., 2021; Niederholtmeyer, 2022). SynBio is guided by the key principles of standardization of parts, modularity and abstraction, rational design and modeling, and the Design-Built-Test-Learn (DBTL) cycle (Matzko and Konur, 2024).

It marks a transformative shift from traditional *ad hoc* genetic modification to a more predictable and systematic engineered biology, comparable to step-changes brought by the integrated circuit in microelectronics and the internet in IT and Computer Science (Benner and Sismour, 2005; Riolo and Steckl, 2022; Sun et al., 2022; Trump, 2020; Voigt, 2020).

In April 2025, the U.S. National Security Commission on Emerging Biotechnology (NSCEB) published its final report, identifying knowledge and technical gaps needed to unlock biotechnology’s national security, economic, and defense potential (National Security Commission on Emerging Biotechnology, 2025). The SynBio industry was valued USD 19.55 billion in 2025, and is expected to rise to USD 96.66 billion by 2035 (Nova One Advisor, 2026), with a projected annual growth rate of up to 28.63% between 2025 and 2033 (Precedence Research, 2025). Rapid advances in 3D printing, robotics, and AI are further accelerating progress in SynBio (Brockmann et al., 2019; De Haro, 2024).

The convergence of SynBio and the aforementioned technologies has profound biosecurity implications with unique threat profiles (see National Academies of Sciences, Engineering, and Medicine, 2018; 2025). The modification of bacteria and viruses, enhancing their pathogenicity, and creation of novel toxins or biological agents can result in the emergence of novel bioweapons (BW) with a heightened threat potential that may find application in military contexts. Drones can be repurposed to disperse BW agents, posing a national and international biosecurity threat. Their broad availability offers a cost-effective delivery method and lowers access barriers for state and nonstate actors. Drone-deployed BW agents pose dispersed, decentralized threats and new challenges to detection, deterrence, and response systems, enabling scenarios like targeted biological attacks and drone-assisted assassination or sabotage (DiEuliis and Giordano, 2022; Pethő-Kiss, 2022).

### 1.2 AI-enabled bioweapon (BW) agents

Key Public Health risks include the development of novel toxins, the emergence of new viral pathogens and vectors, and targeted modification of existing pathogens or their proteins (National Academies of Sciences, Engineering, and Medicine, 2018; 2025). These modifications mainly aim to increase the effectiveness of dissemination and pathogenesis of these agents by expanding the host range, increasing affinity for target cells, and evading immune responses (Thadani et al., 2023).

Especially AI tools, such as large language models (LLMs) and biological design tools (BDTs), are unlocking new possibilities (Carter and Butchello, 2026; Tegomoh, 2025). A crucial difference between LLMs and BDTs is the training data and the output. According to Sandbrink (2023), there exists a foundational differentiation between AI-based models. LLMs act primarily as barrier-lowering technologies for creating or manipulating pathogens, making sophisticated misuse more accessible. They democratize access to DU biological knowledge (Soice et al., 2023) by reducing technical barriers, extracting expert insights, and speeding up the diversification and codification of expertise (Brent and McKelvey, 2025; Hattoh et al., 2025; Zhang et al., 2025).

In contrast, BDTs are capability-raising tools that lead to an uplift of potential malicious actors, bringing with them distinct technical, operational, and biosecurity risk profiles (Batalis, 2023; Moulange et al., 2024). This category of instruments encompasses those utilized for the design of DNA, biological circuits, and cells for biomolecule production in bioreactors (Carter et al., 2023). BDTs increase the speed and precision of accessing biological knowledge. They also enable the design of advanced BW agents, broaden scientific expertise, and substantially reduce the time needed for experiments (National Academies of Sciences, Engineering, and Medicine, 2025; Rose and Nelson, 2023).

Subsequent studies have widely adopted and expanded on this conceptual differentiation (Bloomfield et al., 2024; De Haro, 2024; Groff-Vindman et al., 2025; Moulange et al., 2023; O’Brien and Nelson, 2020; Pannu et al., 2025; Pannu et al., 2024a; Soice et al., 2023; Wheeler, 2025; Wittmann et al., 2024). Empirical demonstrations, such as the prompts-to-pandemic proof-of-concept experiments involving LLMs (Gopal et al., 2023; Grant, 2025; Soice et al., 2023) support the assertion that LLMs substantially lower the technical barriers to misuse and expand access to DU biotechnology. They can contribute to BW risks (Brent and McKelvey, 2025). Concurrently, experimental scenarios involving BDT-mediated protein design misuse underscore vastly different points of concern (Baker and Church, 2024; Stevens, 2026; Webster et al., 2025; Wittmann et al., 2024).

For example, it has been recently demonstrated that AI-driven methods, especially BDTs, can generate novel proteins with tailored properties, such as improved thermal stability, solubility, (Drexel and Withers, 2024; Sumida et al., 2024; Watson et al., 2023), and binding specificity to defined target structures (Zambaldi et al., 2024). Using AI-assisted tools to precisely modify proteins may shorten the development cycles of BW-relevant agents and further amplify the risks associated with DU research. In addition, AI models, such as CRISPR-GPT (an LLM employed for the automated planning of gene editing experiments (Huang et al., 2024)) and BioGPT (an LLM trained on biomedical literature; (Luo et al., 2022)), hold the potential to enhance the efficacy of gene editing experiments in this context.

Furthermore, robotics and automation empower scientists to access, generate, and share knowledge on an unprecedented scale and speed. They further amplify these risks by democratizing advanced bioengineering techniques, facilitating both legitimate and potentially harmful SynBio applications (Groff-Vindman et al., 2025). They can also rapidly accelerate the DBTL-cycles and enable autonomous laboratory operations (Hynek, 2025). A recent example is the Biological Multi-Agent Robotic System (BioMARS), an intelligent platform that integrates LLMs, Vision Language Models (VLMs), and modular robotics to autonomously design, plan, and execute biological experiments (Qiu et al., 2025).

In the future, the detection and eradication of AI-enabled BWs could pose significant challenges (Pannu et al., 2024b). Therefore, intensified research aimed at detecting such novel agents and advancing appropriate safety and control measures is urgently needed (Drexel and Withers, 2024; Thadani et al., 2023). In response to emerging biosecurity challenges, robust cybersecurity measures are essential to prevent misuse and underpin the advancement of the evolving field of cyberbiosecurity (Murch et al., 2018). Cyberbiosecurity addresses the unique risks that arise at the intersection of the life sciences and digital technologies. These risks include threats to genetic databases, laboratory automation, and AI-powered biological platforms, which have the potential to cause real-world Public Health impact (Adam and McArthur, 2024; AIRE, 2025; Richardson et al., 2019; Schabacker et al., 2019).

### 1.3 Biosecurity and interdisciplinary strategies: problem definition and research questions

Despite the strong consensus in the field regarding the existence of these aforementioned risks and biosecurity implications, there is only moderate agreement on how close the field is to catastrophic misuse scenarios (AIxBio Global Forum, 2025) and several points of contention remain. The immediate risk magnitude posed by current frontier LLMs is debated. Bloomfield et al. (2024) acknowledge significant risks yet do not declare imminent catastrophic capability. However, some authors argue that existing models provide sufficient guidance for stepwise pathogen construction (Brent and McKelvey, 2025; Brent et al., 2024). This results in differing policy urgencies. Furthermore, some analyses emphasize “combinatorial” or “cascade” risks when LLMs and BDTs are used together (Groff-Vindman et al., 2025; Undheim, 2024), while others consider them largely distinct governance challenges, fueling debate over the appropriate regulatory scope (Trump et al., 2026). As barriers related to capabilities and resources diminish, the motivation and intent of threat actors are increasingly becoming the decisive factors shaping the biosecurity threat landscape.

The dynamic relationship between risk factors arising from accelerated scientific and technical advancements and the slow pace of governance adaptation to a changing threat landscape are mentioned in earlier studies (Brockmann et al., 2019; de Lima and Quaresma, 2025; World Health Organization, 2022a). There is an ongoing effort as demonstrated by recent policies and established biosafety and biosecurity governance measures (Table 1) and proposed elements for enhancing biosecurity currently dominating the discourse on the governance of AI-enabled SynBio and other converging technologies (Table 2). However, an overarching governance framework capable of addressing the complex and dynamic demands of modern SynBio- and technology-related risks is missing (Bromberg et al., 2025; DiEuliis, 2024; Undheim, 2024). Unfortunately, existing governance frameworks are being surpassed by the rise of intangible code and *in silico* workflows (Bloomfield et al., 2024; Hunter, 2024; Sun et al., 2022; Yassif et al., 2023). Therefore, ongoing empirical and theoretical work is essential to refine governance strategies and align international policy responses to safeguard against misuse without hindering scientific progress.

**TABLE 1:** Established biosecurity governance frameworks, treaties and initiatives (Table is non-exhaustive).

Framework/Treaty/Initiative	Objectives	Reference/Source (type)
WHO Global Guidance Framework for the Responsible Use of Life Sciences	Responsible research; risk governance; dual-use prevention; ethical oversight; capacity building; lifecycle governance of biotechnology; laboratory biosafety and biosecurity practices; agent/material control; prevention of misuse; safety standards in research labs	World Health Organization (2010), World Health Organization (2022a), World Health Organization (2024a)
Responsible Life Sciences Research for Global Health Security Guidance (WHO/NIH/NCBI)	Responsible science; dual-use risk management; governance mechanisms; ethics and controls in research	World Health Organization (2010)
FAO Biosecurity Framework / Toolkit Progressive Management Pathway for Terrestrial Animal Biosecurity (PMP-TAB)	Integrated biosecurity across agriculture, food, livestock, plants, environment; one health; national systems and capacity; stepwise implementation of livestock and animal-sector biosecurity; one health integration; disease prevention; AMR reduction	Food and Agriculture Organization of the United Nations (2007), Food and Agriculture Organization of the United Nations (2023)
IPPC – International Plant Protection Convention	Prevent introduction and spread of plant pests and invasive species; phytosanitary standards; global coordination	International Plant Protection Convention (2026)
GHSA – Global Health Security Agenda	Strengthen national capacities for detection, prevention, response; implement international health regulations; pandemic preparedness	Global Health Security Agenda (2025)
One Health Governance (EU scientific advisors/EC initiatives)	Cross-sector governance (human-animal-environment); surveillance; risk analysis; integrated responses; research and biosafety	European Commission (2025)
IBBIS – International Biosecurity and Biosafety Initiative for Science	Biosecurity in biotechnology/synthetic biology; DNA screening; certification and traceability; global governance proposals	The International Biosecurity and Biosafety Initiative for Science (2025)
IGSC – International Gene Synthesis Consortium	Industry-led organization to safeguard biosecurity, apply a common protocol for screening DNA sequences and customers while promoting the beneficial use of gene synthesis	The International Gene Synthesis Consortium (2020)
National biosecurity strategies (example: Nigeria NBMA)	National regulation of biological risks, agents and labs; surveillance; emergency response; cross-ministry governance	National Assembly of the Federal Republic of Nigeria (2015), National Assembly of the Federal Republic of Nigeria (2019)
Technical Advisory Groups	Advisory mechanisms for biosafety and biosecurity standards; global guidelines; strategic recommendations; e.g., WHO-TAG-b; national science advisory board for biosecurity, (NSABB, USA); national biodefense science board (NBSB, USA); advisory committee on dangerous pathogens (ACDP, UK); engineering biology responsible innovation advisory panel (RIAP, UK)	e.g., World Health Organization (2023), Administration for Strategic Preparedness and Response (2025), Gov UK (2023), Gov UK (2025a)
Regional/international CBRN and biosafety initiatives	Cross-border coordination; capacity building; bio surveillance; preparedness; standardized protocols; e.g., EU CBRN centres of excellence initiative	The European Union (EU) (2025)
Biological Weapons Convention (BWC)	Prohibits biological weapons development and possession; prevents deliberate misuse of biological agents; disarmament and non-proliferation	United Nations Office for Disarmament Affairs (1972)
CBD – Convention on Biological Diversity	Protect biodiversity; sustainable use of biological resources; governance of genetic resources	United Nations (1992)
Cartagena Protocol on Biosafety (to the CBD)	Regulates transboundary movement and use of GMOs/LMOs; biosafety; precautionary principle; risk assessment; biosafety clearing house	United Nations (2000)

**TABLE 2:** Proposed governance elements to enhance biosecurity governance (Table is non-exhaustive).

Governance element	Objectives	Reference
Strengthening international capabilities to prevent biological weapons development and use	Creating a scientific advisory mechanism; enhancing transparency, improving attribution, and promoting accountability for violations of the global norm against the development and use of bioweapons	Forman et al. (2025), Lentzos et al. (2025), López-Vergès et al. (2025), Shearer et al. (2025), Yassif et al. (2021)
Global but decentralized oversight	Polycentric and decentralized regulatory oversight adapted to an increasing number of domains and actors and anticipatory governance	Douglas and Stemerding (2014), Ienca and Vayena (2018), Lee (2025)
International and cross-sectoral cooperation	Governance frameworks that promote international harmonization and sectoral coordination, reflecting the transboundary and interdisciplinary nature of biosecurity threats	Hamilton et al. (2021), Hulme (2021), Li (2025)
Differentiated regulatory framework	The heterogeneity of threats across technical domains and actor capabilities necessitates specialized, rather than uniform, governance measures	Ienca and Vayena (2018)
Mixed approach	A balanced mix of precautionary (command-and-control, hard law), stewardship (soft law), bottom-up, and laissez-faire (industry-driven) approaches	Undheim (2024), Wang (2025)
Managing convergence risks via capacity building as well as adaptive and coordinated governance based on flexibility and agility	Raise awareness about biorisk management; accelerate the establishment of professional biosecurity research institutions; provide specialized educational resources, and promote the training of relevant personnel	Breyer et al. (2024), Elgabry and Johnson (2024), Richardson et al. (2019), World Health Organization (2022b)
	Create an international, adaptive governance framework with evidence-based regulatory mechanisms to address the ethical, legal, and existential challenges posed by converging cyberbiological systems	Helbing and Ienca (2024), OECD (2024), Reuel and Undheim (2024)
	Agile policy responses and continuous adaptation to emerging risks and technological innovation; experimentation and policy iteration guided by empirical feedback	Cook et al. (2010), Dittrich (2024), OECD (2024)
Polycentric and overlapping jurisdictions	Distribute authority and decision-making across multiple levels to ensure responsiveness and resilience	Cook et al. (2010), Daniell and Kay (2017), Ienca and Vayena (2018)
Top-down elements	Provide resources, coordination, and infrastructure to empower subnational and non-governmental actors	Hamilton et al. (2021)
	Establish regulatory oversight and monitoring, including rapid escalation and crisis management when needed	Gillum (2025a); OECD (2024)
	Establish legally binding treaties, and independent regulatory bodies with enforcement capabilities	Li (2025), Ou and Guo (2023)
Bottom-up elements	Establish self-regulatory guidelines and codes of conduct (e.g., tianjin biosecurity guidelines for codes of conduct for scientists); implement principle of caution and vigilance in biotechnology governance through self-discipline within the scientific community and voluntary acceptance of biosecurity oversight by scientists	Elgabry et al. (2025), UN Information Service (2005), Sun et al. (2022), Wang et al. (2021)
	Empowerment of local actors, including communities, laboratories, and industries, to adapt risk management to local circumstances	Ienca and Vayena (2018), Rawluk et al. (2021), Yoshizawa et al. (2024)
	Promotion of shared responsibility and partnerships among diverse stakeholders, leveraging local knowledge, surveillance, and rapid feedback mechanisms	Rawluk et al. (2021), Yoshizawa et al. (2024)
	Facilitate stakeholder dialogue and participatory decision-making around emergent risks and technology deployment; establishment of “engineering biology responsible innovation advisory panel”	Gov UK (2025b), Yoshizawa et al. (2024)
Dynamic information flow	Lateral communication and real-time data sharing across governance levels to enable the swift detection of threats and the dissemination of best practices	Cook et al. (2010), Rawluk et al. (2021)
Incentive alignment	Coordination of incentives across actors, from policymakers to local practitioners, to ensure the adoption and compliance of biosecurity measures	Cook et al. (2010), OECD (2024)
Technical and ethical integration	Adaptive frameworks that incorporate technical monitoring tools, ethical guidance, and codes of conduct which are periodically reviewed in light of new technological developments	Cook et al. (2010), Gillum (2025a), Li et al. (2023), Reuel and Undheim (2024)
Experimentation and policy innovation	Pilot projects and regulatory sandboxes which allow for the safe testing and gradual implementation of new solutions, ensuring the framework keeps pace with innovation	OECD (2024), Zhou et al. (2024)
Multi-stakeholder partnerships	Partnerships that are co-designed and enacted by governments, scientific and industrial communities, civil society, and international organizations to foster buy-in and resilience across scales	Breyer et al. (2024), OECD (2024), Rawluk et al. (2021)
“Web of prevention” and layered risk management	An integrated biosafety-biosecurity governance concept that links international and national measures to prevent the accidental or deliberate misuse of life sciences; polycentric systems ensure that if one layer fails (e.g., national oversight), others (e.g., local or institutional controls) will remain operative	Bezuidenhout (2012), Cook et al. (2010), Daniell and Kay (2017), Novossiolova et al. (2021), Rappert and McLeish (2012), Weir and Selgelid (2009)
Continuous learning and feedback	Imbedding mechanisms for feedback, policy evaluation, and iterative improvement to enhance adaptive capacity and responsiveness	Attal-Juncqua et al. (2025), Cook et al. (2010), OECD (2024)
Two-tier system	National-level oversight and institutional-level oversight	World Health Organization (2024a)

Especially in the realm of SynBio, AI, and emerging technologies, the potential biosecurity implications of scientific and technological advancements, as well as the necessary governmental and regulatory measures, remain underexplored (AIxBio Global Forum, 2025). This study addresses this gap by applying the Delphi process, a structured, iterative approach for expert elicitation and stakeholder involvement (Dalkey and Helmer, 1963; Khodyakov et al., 2023; Linstone and Turoff, 1976; Rowe and Wright, 2001) to systematically identify potential biological threats, assess biosecurity risks, and generate consensus-based policy recommendations.

This method has been effective in forecasting scenarios of emerging crime trends facilitated by biotechnology (Elgabry and Johnson, 2024; Elgabry et al., 2022), the assessment of DU risk factors of SynBio (Zhang et al., 2024), assessing the bioweapon threat (Boddie et al., 2015) and policy development, such as Brown et al.’s (2006) policy recommendations for biological incident response, and Zhang et al.’s (2024) development of a global code of conduct to reduce DU-SynBio-risks.

The current study focuses on two research questions:

1. How might the continuing evolution of SynBio and related converging technologies influence future biosecurity vulnerabilities, particularly with regard to deliberate misuse or the development of novel biological threats?
2. What governance approaches are required to develop a holistic, adaptive framework that effectively mitigates biosecurity risks emerging from SynBio and related converging technologies?

Hence, the main goal was to develop an overarching governance framework to mitigate biological threats from SynBio-driven BW. Since the scientific and technological developmental landscape is changing rapidly, a wide range of differing opinions and conflicting views were to be expected. We therefore introduced the concept of tendential consensus in our Delphi process. We define a tendential consensus as a collective understanding that falls short of unanimity in Likert-scale questions (see Section 2.2.6).

Our generated findings are integrated into the existing recommendations and partially well-established governance elements (Table 2). These contribute to a new and more comprehensive governance framework for managing biosecurity risk arising from the intersection of SynBio and emerging technologies. Moreover, they offer valuable insights for policymakers and stakeholders in safeguarding Public Health and security amidst rapid technological progress and global challenges.

## 2 Methods and materials

Given the future-oriented nature of the present inquiry and the paucity of empirical data, the research employs a foresight approach. The Delphi method was used to explore scenarios related to potential biothreat landscapes arising from SynBio and other emerging and converging technologies.

### 2.1 Literature review and preliminary analysis

A comprehensive literature review was conducted to serve multiple purposes:

- To establish a foundational understanding of the historical development and use of BW.
- To examine the role of rapidly evolving and converging technologies - including additive manufacturing (3D printing), robotics, AI, laboratory automation, and nanotechnology - in accelerating the development and capabilities within SynBio. Special emphasis was placed on analyzing the accessibility and availability of relevant knowledge and technological advancements that could facilitate the development of SynBio-driven BW.
- To identify the most important and prominent scientific and technological drivers, expected biosecurity implications, indicators, and structural trend shifts with biosecurity implications, such as the potential development of SynBio-enabled BW (Amanatidou et al., 2012).
- To identify biosecurity experts for the Delphi-Expert panel;

### 2.2 The Group Delphi process

The Group Delphi process evaluates current knowledge (Niederberger and Spranger, 2020), especially uncertain and complex issues (Nelson et al., 2021; Steinmüller, 1997) and facilitates consensus-building around future scenarios (Dalkey and Helmer, 1963; Niederberger and Deckert, 2022). It enables the systematic collection of expert opinions through a multi-stage process iteratively aggregating anonymous expert judgments via interviews and surveys (Linstone and Turoff, 2002). While with each round, views based on prior collective input are refined, anonymity ensures equal weighting of contributions, mitigating dominance bias from influential individuals (Winkler and Moser, 2016).

**FIGURE 1:**
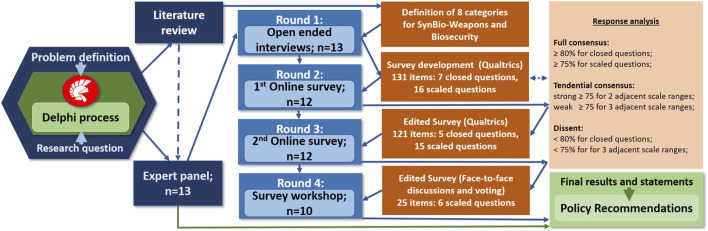
Structure of the conducted Delphi process.

We conducted interviews and two rounds of online surveys, as well as two virtual group workshops. Eight key topics and research questions on SynBio-enabled weapons and biosecurity were identified (see Supplementary Material S1) to refine the focus throughout the Delphi rounds. Additionally, the literature review provided a baseline for interpreting the Delphi results in subsequent stages of the study. The gained insights were integrated into the survey to ensure alignment with the current state of research. The systematic structure of the Delphi process is depicted in Figure 1. Furthermore, the study explored possibilities to enhance governance frameworks through a survey workshop. The recommendations derived from this process were further elaborated to provide valuable insights for policymakers to effectively address current and emerging biosecurity threats.

#### 2.2.1 Expert panel

To identify suitable experts for this study, a targeted search was conducted within German-speaking countries. While criteria for defining expertise vary (Shang, 2023), we focused on individuals from various disciplines in the fields of biosafety and biosecurity some of which are participants of the Biological Weapons Convention (BWC) working group meetings. We did not include specific experts on the various converging technologies discussed, because of our focus on biosecurity (see “Limitations” section). Due to high attrition rates in Delphi studies (Loë et al., 2016) and to ensure a comprehensive and diverse range of perspectives, the snowball method (referrals from participants; Denscombe, 1997) was employed. The selection criteria emphasized professional qualifications, requiring candidates to hold an advanced academic degree (e.g., at least a Ph.D. or equivalent) and/or possess extensive practical experience in the relevant field (Avella, 2016; Okoli and Pawlowski, 2004; Watson et al., 2017). Additionally, publication activity was considered essential, with experts expected to have authored several peer-reviewed publications or reports related to the study’s subject matter. Finally, the reputation and influence of candidates were evaluated, considering recognition by peers, membership in relevant professional societies, and involvement in advisory roles. These stringent criteria ensured the inclusion of highly qualified experts from Germany, Austria, and Switzerland, and a suitable panel size of 8–20 participants (Shang, 2023), capable of contributing valuable insights to the research. Although we were aware of the “homophily bias” (Holeman et al., 2024) when selecting participants, we refrained from recruiting non-traditional experts (Elgabry et al., 2025) due to the dedicated topic of potential SynBio-enabled BW risks.

Out of 25 contacted experts, 13 (52%) participated in the interviews. The expert panel was aged between 20 and 80 years, consisting of four women and nine men with different affiliations, divided into governmental, non-governmental, industrial, military, and academic (Table 3). 12 (92,3%) of them took part in the online surveys, and 10 (76,9%) of them participated in the online workshops. 12 out of 12 experts (100%) consider themselves to have moderate to high comprehensive knowledge in biosecurity, 9 (75%) of them in conventional biological warfare agents, and 7 (58,3%) of them in SynBio warfare agents (Supplementary Figure S3).

**TABLE 3:** Affiliations of the expert panel members.

Affiliation type	Institution organization	Description
Governmental	Friedrich-Loeffler-Institute (FLI)	Key role in veterinary public health and biosafety research
	Robert Koch Institute (RKI)	A national public health institute responsible for monitoring, preventing, and responding to infectious diseases. It conducts research epidemiology, health monitoring, and health protection
	Spiez Laboratory (Swiss Federal Institute for NBC-Protection)	Internationally respected research and security institution focused on biosecurity and nuclear, biological, chemical (NBC) threats; strong civil protection orientation
	SPRIND (Federal Agency for Disruptive Innovation)	Identifies and funds high-risk, breakthrough innovations and drives government-funded innovation, including SynBio
Military	Austrian Federal Ministry of Defense (BMLV)	Responsible for military defense and national security in Austria; specializes in CBRN (chemical, biological, radiological, nuclear) defense
	Bundeswehr Institute of Microbiology; Department for Medical Bio-Reconnaissance and Bioforensics	Responsible for medical intelligence on biological threats, verification of potential biological warfare agents, and assessment of biological hazards in defense/security contexts
Non-governmental organization (NGO)/Academic	German Association for Synthetic Biology (GASB)	Academic/scientific association founded by SynBio researchers to promote and connect Germany’s SynBio community
	German National Academy of Sciences Leopoldina	Provides independent, evidence-based reports and statements on scientific and social issues (e.g., emerging and converging technologies, health policy, and digitalization); offers policy advice on scientific and socially relevant issues
NGO/Advisory	Nuclear Threat Initiative (NTI) and International Biosecurity and Biosafety Initiative for Science (IBBIS) (represented by a biosecurity consultant)	Focuses on international biosecurity and biosafety initiatives
Industry	Gene Art (former director) and international gene synthesis consortium (IGSC) co-founder	An industry-led organization to ensure biosecurity, standard screening protocols for DNA sequences and customers, and responsible use of gene synthesis
Academic	Bernhard Nocht Institute for Tropical Medicine (BNITM)	Specializes in tropical medicine, focusing on infectious diseases such as malaria, ebola, and other pathogens endemic to tropical and subtropical regions
	Charité	Leading center for medicine, research, and teaching in Germany and worldwide
	Karlsruhe Institute of Technology (KIT)	Combines academic education and cutting-edge research; member of the Helmholtz association; one of Europe’s top technical universities

#### 2.2.2 Structured interviews

The interviews were conducted online in February 2024. Each expert was interviewed individually by two interviewers. The interview covered topics in biosecurity, SynBio, AI, and technology governance (see Supplementary Material for more details). The interviews were kept as open as possible, allowing the experts to focus on aspects they found most relevant. Experts were not given the questions in advance, ensuring they relied on their prior knowledge and could respond freely. The interviews were conducted via Zoom and recorded in both video and audio.

#### 2.2.3 Analysis

The interviews were transcribed using Microsoft Word’s transcription feature, manually corrected, and processed in MAXQDA 2022, a software for computer-assisted qualitative thematic data analysis (Gizzi and Rädiker, 2021). Applying the four-eye principle, verbatim responses were sorted by question and coded manually into thematic categories, resulting in a total of 2.322 codes from the interviews (Gizzi and Rädiker, 2021; Kuckartz and Rädiker, 2022).

#### 2.2.4 Online survey

The results of the interviews were used to create an online survey. This served to gather expert opinions, to identify the consensus topics within the expert panel, and to enable the formulation of transparent and consistent threat prioritizations, along with recommendations grounded in evidence for policymakers (Montibeller et al., 2020). The online survey was created and conducted using Qualtrics software (XM). The survey covered 23 questions with a total of 131 items concerning eight categories: prior knowledge (1 question); threat perception (4 questions); technological and scientific advancements (4 questions); security gaps (1 question); biosecurity implications (6 questions); government and regulatory measures (4 questions); public awareness and education (2 questions); and future-oriented actions (1 question) (see Figure 1).

To enable the use of consensus criteria, 18 open questions were rated on various Likert scales (e.g., “very low” to “very high”) (Romero-Collado, 2021). Most open questions used five-point scales to reduce distortions typically caused by overly fine gradations. Three questions used three-point scales for lower complexity, and two questions required ranking five or seven items to offer sufficient differentiation. To complement Likert-scale questions and reduce ambiguity, seven closed questions requiring “yes” or “no” answers, were included. Questions that achieved consensus among the experts were eliminated from the second round of the survey. Hence, the second survey entailed 20 questions with 121 items, including five closed questions (Figure 1).

The questions were designed to be clear and concise, ensuring that relevant responses were obtained for the study’s objectives. The questionnaire length was kept reasonable, to complete it in 15–20 min to reduce dropout rates (see Supplementary Material S1 for the full survey).

#### 2.2.5 Survey data analysis

The survey results were exported from Qualtrics into Microsoft Excel and analyzed. Data were presented in grouped and stacked bar charts. Where appropriate, rankings were created by assigning point values to responses, which were multiplied by the number of answers and totaled.

#### 2.2.6 Consensus process

For response analysis, at least 80% agreement for closed (yes-or-no-) questions was set as the threshold for “full consensus”, whereas at least 75% was necessary for scaled question consensus (Figure 1) as commonly practiced (Avella, 2016; Diamond et al., 2014; Niederberger and Deckert, 2022). To refer to a situation in which the expert group leans toward a particular opinion or agreement without reaching a complete, formal consensus, in this study, we introduced the concept of “tendential consensus”. It implies a shared understanding or decision rather than unanimous agreement. Strong tendential consensus required at least 75% agreement within 2 adjacent scale ranges, while weak tendential consensus required at least 75% agreement within 3 adjacent scale ranges. Consequently, “dissent” prevailed if less than 80% reached the same result for closed questions, or less than 75% in 3 adjacent scale ranges in open questions (Figure 1).

#### 2.2.7 Workshops

To ensure common ground among all participants, we developed three BW scenarios based on red team exercises (Moran, 2021; Zhang and Gronvall, 2020), involving BW agents such as bacteria, bioregulators, and gene drive-yeasts involving varying degrees of complexity in their acquisition, development and deployment. The expert panel was given 2 weeks to evaluate the scenarios based on the following criteria: BW agent development, release, human health impact, societal impact, economic impact, environmental impact, and mitigation. A virtual scenario workshop was held in April 2024, in which the BW scenarios were thoroughly discussed, with a special emphasis on converging technologies and SynBio (see Supplementary Material S1). Based on this experience, a fourth virus-based biothreat scenario was developed together with the expert panel. The results are being withheld due to potential information hazards.

The expert judgements and prioritization rankings derived from the online surveys were then discussed during a virtual survey workshop in August 2024 to formulate evidence- and consensus-based recommendations for policymakers (see Supplementary Material S1). During this workshop, survey outcomes of certain questions with clear dissent were further discussed in contrast to items with tendential consensus, to prevent false consensus due to biases (Winkler and Moser, 2016).

### 2.3 Development of an overarching governance framework

Combining the results from the literature review and the Delphi process, especially the individual policy recommendations, we have subsequently elaborated an overarching governance framework. We have opted to integrate the recommendations into a cyclical approach. This approach entails the ongoing process of identifying, assessing, and addressing risks associated with this rapidly evolving field of SynBio and other emerging and converging technologies. Its aim is to counter potential biological threats while managing the complex requirements of modern bio- and technology-related progress.

## 3 Results

### 3.1 Delphi process

In the following, only the most relevant results from the Delphi study are presented, especially those majorly affecting the subsequent policy recommendations. For the comprehensive results, see the Supplementary Material S1.

In order to evaluate the importance and threat connected to synthetic biowarfare agents, we provided our experts with a set of hypothetical biothreats and asked them which they find most concerning. These biothreats entailed natural or genetically modified pathogens that are either naturally occurring, unintentionally, or intentionally released. When asked to rank their concerns, experts tended to be most concerned about naturally occurring pathogens, followed by unintentionally and then intentionally released natural pathogens. The latter is equally concerning as the unintentional release of genetically modified pathogens, while the experts are least concerned about the deliberate release of genetically modified pathogens (Table 4, also see Supplementary Figure S8).

**TABLE 4:** Biothreat ranked by the experts’ concern.

Origin/Release pattern	Natural pathogen	Genetically modified pathogen
Naturally occurring	54	N/A
Unintentional release	42	39
Intentional release	39	31

We further asked whether awareness of this subject is sufficient among experts. There is a clear consensus that awareness of BW in general and synthetic biowarfare agents specifically is insufficient among experts. There is, however, dissent regarding the awareness of general biothreats.

When asked to predict a time frame in which synthetic biowarfare agents might become more relevant than conventional biowarfare agents, eleven out of twelve experts would say within the next 50 years. Out of these eleven experts, eight (73%) would even place synthetic biowarfare agents within the realm of possibilities within the next 10 years (Supplementary Figure S12).

The experts assessed the most impactful drivers for biological threats posed by synthetic BW as the technological and scientific progress, the greater availability of technologies, the continuously decreasing costs of technologies, and the limited or inadequate control measures (Supplementary Figure S16).

Moreover, the impact of the pace of technological progress, especially SynBio and other emerging and converging technologies was rated as moderate to high. Given the continuous and rapid progress of technology, numerous current developments in SynBio have profound implications for biosecurity. The experts identified simplified handling, decreasing costs, accelerated development, and greater availability as having the strongest impact on the potential threat arising from synthetic biowarfare agents (Supplementary Figure S20).

The experts identified AI-modified protein design tools, e.g., AlphaFold, synthetic chemistry and machine learning (ML) for reaction prediction, and LLMs as the most relevant converging technologies, followed by automation and robotics (Supplementary Figure S28). At the same time, the three most relevant advances that may lead to biosecurity problems within 10 years were identified as bench-top DNA synthesizers, Gain-of-Function (GOF) research, and synthetic bioregulators, followed by *de novo* synthesis of nucleic acids and modular biology with kits for everything and everyone (Supplementary Figure S24).

**FIGURE 2:**
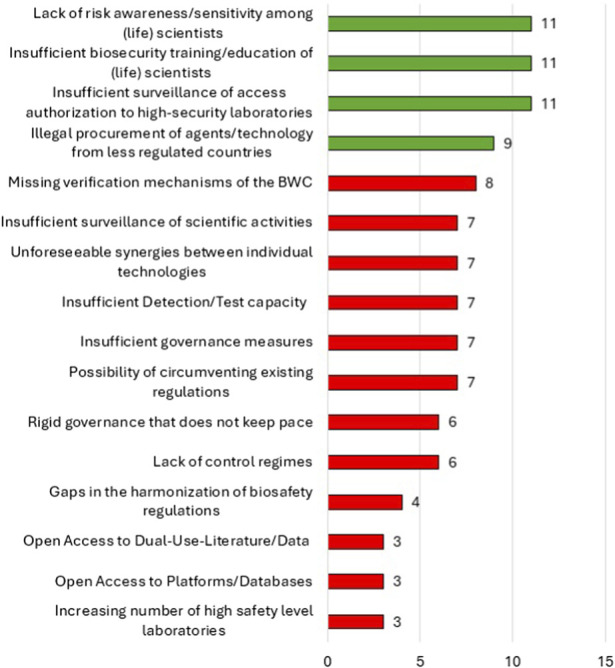
Ranking of relevant vulnerabilities that could facilitate misuse for the production of synthetic biowarfare agents and weapons, after the second survey round. Green items were voted on with consensus. Red bars indicate dissent.

In terms of misuse, out of many items, the experts consensually determined that lack of risk awareness, insufficient biosecurity training, and inadequate monitoring of access authorization to high-security laboratories as the most relevant biosecurity vulnerabilities, followed by the potential illegal procurement of agents or technology from less-regulated countries, as well as the lack of verification instruments under the BWC (Figure 2).

It is the experts’ consensus view that current (as of 2024) biosafety legislation is sufficient (Supplementary Figure S45). However, they also consensually estimate that existing regulations and safeguards will not keep pace with the rapid advances in SynBio (Supplementary Figure S38). Additionally, there is a consensus that governance measures are currently unable to be adapted with sufficient flexibility and promptness to the dynamically changing science and technology landscape, thus jeopardizing the prevention of the development and deployment of synthetic biowarfare agents (Supplementary Figure S47). Similarly, the ability of the scientific community to detect advances and drivers that could facilitate the development of synthetic biowarfare agents, and the ability of states to identify and appropriately respond to it, are both rated as low to moderate (Supplementary Figure S40).

**FIGURE 3:**
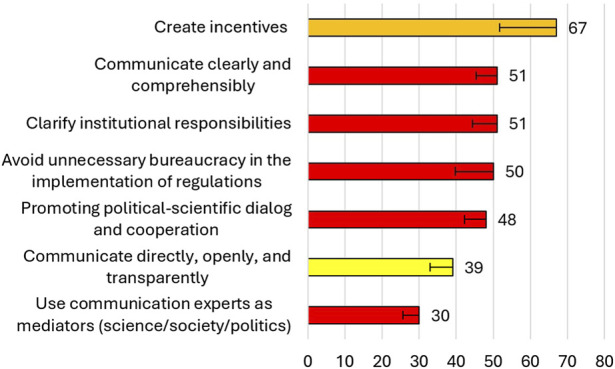
Ranking of ideas on how awareness of biorisks could be raised after the second survey round. Red items did not achieve consensus; the yellow item were voted on with strong tendential consensus, the orange item was voted on with weak tendential consensus. Error bars represent the standard deviation depicted only in one direction for clarity.

Although the expert panel agreed that raising awareness is the most effective approach by governments and international organizations to improve global biosecurity, the experts did not reach a consensus on how to raise awareness of biorisks. Through a weak tendential consensus, incentives were suggested as a measure to raise awareness of biorisks. This was followed by a weak tendential consensus regarding clear and comprehensive communication, as well as clarified institutional responsibilities (Figure 3).

**FIGURE 4:**
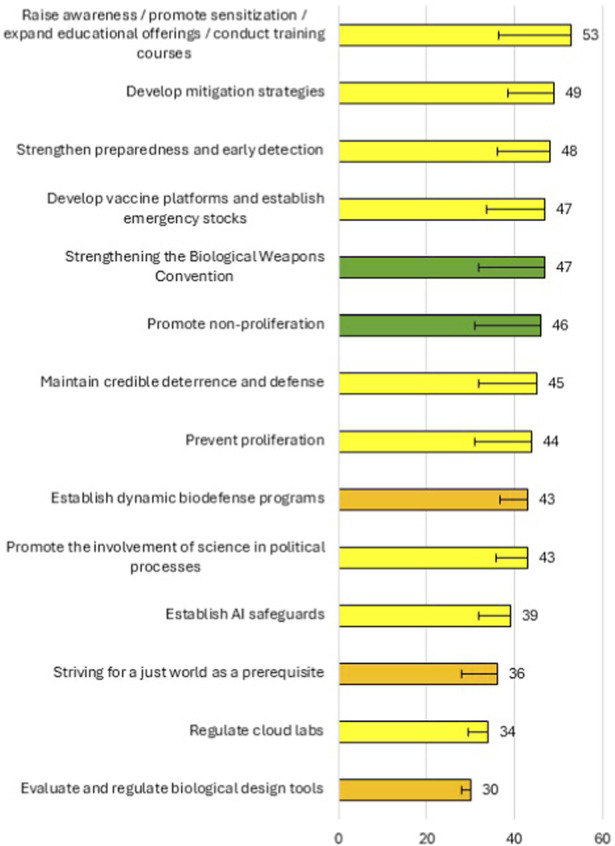
Ranking of the effectiveness of different approaches to improve global biosecurity, after the second survey round. Yellow items achieved strong tendential consensus. Orange items achieved weak tendential consensus. Green items achieved full consensus. Error bars represent the standard deviation depicted only in one direction for clarity.

According to the ranking, the most effective measure to improve global biosecurity is to create awareness through sensitization, expand education, and conduct appropriate training of research staff. This is followed by the development of mitigation strategies and strengthen preparedness and early detection, as well as by developing vaccine platforms and establishing personal protection equipment (PPE) emergency stocks. However, only a strong tendential consensus was reached on these biosecurity measures. Yet, there was consensus on the approaches of strengthening the BWC and promoting non-proliferation (Figure 4).

These findings were further discussed and refined with the expert panel in a survey workshop. In this workshop, political recommendations on how to address the identified biosecurity issues were collected and roughly sketched out. From these notes, we afterwards formulated the following elaborate recommendations, which could best be integrated into an overarching governance framework.

### 3.2 Political recommendations to mitigate biological threats from synthetic biology-driven biological weapons

The expert consultations revealed a consensus that rapid advancements in SynBio together with other constantly evolving emerging and converging technologies present significant biosecurity challenges, particularly concerning synthetic BW. These advancements are facilitating the manipulation of pathogens with reduced financial and personnel requirements, and with increasing technical ease. The reduction in access barriers to BW development could broaden the scope of malicious actors. Consequently, there is an urgent need to understand the range of evolving biosecurity risks, to establish a strategic priority list of biological threats, and to develop effective and sustainable solutions to address them. In the age of AI, there is an increased likelihood that these developments will continually outpace the efforts of governments to address these issues. We therefore developed transparent and consistent threat prioritizations, accompanied by evidence-based policy recommendations, some new, some previously discussed (see Tables 1, 2).

#### 3.2.1 Raising awareness through sensitization, education, and incentivization

Insufficient awareness of SynBio and contemporary biothreats among professionals in relevant fields can lead to an underestimation of associated SynBio risks. To address this shortcoming, general awareness-raising campaigns and comprehensive educational programs should be implemented across various sectors, primarily in academia, as well as in government and industry.

Educating scientists about biosafety and biosecurity is key to positive cultural change in industry and academia. Scientists must be aware of their responsibilities, the importance of biosecurity measures and biorisk management must be recognized. The goal is to proactively identify potential risks related to safety, security, and DU in both basic and applied life sciences and to implement suitable mitigation and precautionary measures.

Students and trainees in basic and applied life science, technology, engineering, and mathematics should receive compulsory general education on dual-use research of concern (DURC) and biorisk management principles and competencies from academic institutions. These topics should be integrated into life science curricula at all levels, from undergraduate classes to doctoral research. Moreover, all scientific community members should engage in ongoing education. For instance, employees in public, private, and academic research or diagnostic laboratories, private and public research funders, editors and publishers of research, and the general public should all be targeted for awareness-raising efforts.

Workshops, seminars, and online courses can be utilized to disseminate information about the potential threats and ethical considerations on SynBio. Tailored campaigns to a variety of audiences can positively impact biosecurity behaviors. Additionally, collaboration with international organizations can help standardize training and awareness initiatives globally. This will eventually lead to an enhanced biosafety and biosecurity culture in research and development environments, as well as related fields.

Fostering the knowledge exchange between the biosciences and biosecurity experts can help minimize biological threats. Leveraging existing domestic expertise will not only reduce biosafety and biosecurity risk but will also help support the growing bioeconomy and minimize the misuse potential. Public engagement campaigns can play a crucial role in educating the general population about the implications of SynBio and its DU potential.

Research institutions, funding bodies, and other stakeholders (national governments, publishers and editors, the private sector, and civil society networks, and publics) should mandate and promote ongoing education and training in biorisk management, including standardized risk assessment and risk mitigation techniques. Professional certification in biosecurity and biorisk management could incentivize these endeavors. Similarly, promoting grassroots initiatives by acknowledging or rewarding the most effective biosecurity approach will have an ongoing impact.

Moreover, the presence of multi-stakeholder advisory bodies and the implementation of transparency-driven dialogue processes are crucial elements in this context. We propose the establishment of comprehensive advisory committees that integrate diverse perspectives from non-governmental organizations, academic institutions, industry stakeholders, and citizen groups to inform policy development and strengthen democratic legitimacy in emerging technology governance. These advisory bodies would function as pivotal intermediaries between technical expertise (provided by scientists, risk assessors, and biosecurity specialists) and public interest considerations, including ethical, societal, and legal dimensions, thereby ensuring that governance decisions reflect broader societal values while maintaining scientific rigor. The European Biosecurity Regulators, which unite multiple European organizations to create joint guidance and training resources, may provide a working blueprint for multi-sector governance. In addition to this institutional approach, we advocate for the implementation of systematic transparency and open dialogue initiatives that facilitate public engagement with complex technological developments, enabling discourse on the beneficial applications and potential misuse scenarios of SynBio, AI, and other converging technologies.

Such a multi-stakeholder advisory mechanism can significantly enhance public trust, improve policy effectiveness, and create more responsive governance systems capable of adapting to technological change (IAP, 2024; Moya et al., 2025). The integration of these approaches may offer a pathway toward more inclusive and legitimate governance frameworks that can better manage the DU nature of SynBio and emerging technologies while fostering innovation and protecting Public Health. These should be structured bottom-up on an institutional, regional, national and eventually international level (Box 1).

Box 1 **Recommendations for raising awareness through sensitization, education, and incentivization.**

- **Academic education initiatives:**
  - Launch national and regional campaigns to improve biotechnology literacy, focusing on the dual-use potential of SynBio, AI, and other converging technologies in the creation of bioweapons.
  - Integrate the dual-use topic into university curricula to ensure that the next-generation understands the potential of, and risks associated with, emerging technologies.
- **Professional sensitization:**
  - Mandate regular biosecurity and dual-use awareness training for researchers, technical staff, and industry professionals involved in SynBio and AI.
  - Develop sector-specific guidelines and case studies that illustrate real-world dual-use risks and ethical dilemmas.
- **Incentivization for responsible conduct:**
  - Establish awards, grants, and certifications for institutions and individuals demonstrating an exceptional commitment to biosecurity and responsible innovation.
  - Provide funding for public engagement projects that encourage discussion between scientists, policymakers, and the public about biosecurity issues.
- **Multi-stakeholder advisory bodies:**
  - Set up advisory committees comprising NGOs, academia, industry, and citizen groups to inform policy direction and enhance legitimacy and trust.
  - Structure on an institutional, regional, national and eventually international level.
- **Transparency and open dialogue:**
  - Promote open forums and consultations to discuss advances in SynBio and AI, as well as the potential for misuse and its societal implications.

#### 3.2.2 Improve biosecurity through robust training and monitoring systems

The most pressing weaknesses in biosecurity concerning SynBio include inadequate training, poor monitoring of access authorizations, and a lack of risk awareness among personnel. To mitigate these vulnerabilities, rigorous and conceptual biosecurity training programs and holistic monitoring systems must be established. This entails the implementation of exhaustive training programs, including interdisciplinary concepts, systems thinking, and collaborative methodologies, which must be regularly updated to reflect the latest advancements in SynBio and other converging technologies. The rationale for prioritizing biosecurity and biorisk management must be clear to all stakeholders and governance enhancements pinpointed at individual, institutional, regional, national, and international levels. Training should enable professionals to identify potential vulnerabilities and develop comprehensive biosecurity strategies that address the entire system, rather than isolated components. Likely suitable benchmarks are established guidelines, such as the CDC/NIH Handbook “Biosafety in Microbiological and Biomedical Laboratories (BMBL 6th Edition)” (CDC and NIH, 2020) and ISO-based biorisk management systems.

Concurrently, rigorous assessment methods must be developed to ensure effective monitoring of biosecurity system resilience. This evaluation should comprehensively identify the system’s strengths and weaknesses. Integrating continuous monitoring systems with diverse data sources is crucial for obtaining real-time feedback and improving compliance. The WHO’s “Hub for pandemic and epidemic intelligence” (2021) might be a good example (WHO-HUB, 2021). Digital access logs, biometric identification systems, and even AI could be used by academic institutions, industry, and organizations to track access to sensitive materials and technologies or even detect anomalies (Crawford et al., 2023).

Regular evaluations of the efficacy of biosecurity, as well as biosafety measures on an institutional and regional level are essential, with necessary adjustments being made based on insights from ongoing monitoring data and feedback. It is also imperative to conduct regular risk assessments to identify and address potential vulnerabilities in biosecurity protocols. Furthermore, for AI-assisted tools, continuous audits should be conducted, maybe according to the NIST “AI Risk Management Framework (AI RMF 1.0)” (NIST, 2023).

In addition, we propose the systematic implementation of red teaming exercises and scenario planning as essential practices for identifying vulnerabilities across biological laboratories, biotechnology supply chains, and associated data infrastructure (see, e.g., Elgabry et al., 2022; Mouton et al., 2024; Nelson et al., 2021). These proactive security assessments empower organizations to anticipate and mitigate potential threats before they materialize into actual security breaches. This includes structured testing, documentation of findings and follow-up corrective actions, and complements formal DURC/enhanced potential pandemic pathogen (ePPP) review processes as discussed by the US bodies (National Science Advisory Board for Biosecurity, 2023) in the context of revising biosecurity oversight.

Establishing “regulated sandboxes”, as controlled testing environments, would allow high-risk SynBio and AI applications to be tested in real-world settings under regulatory supervision – with clearly defined safeguards, protocols, and exit criteria. The EU AI Act explicitly provides for AI regulatory sandboxes to combine innovation with risk management and oversight (Article 57 et seq. (EUR-Lex, 2024)).

Institutional boards should systematically review project proposals for DU risks and ePPP/GOF aspects as informed by the current NSABB recommendations (NSABB, 2023). Furthermore, mobile applications for biosafety, biosecurity, and DU oversight may be helpful guides (IEGBBR International Experts Group of Biosafety and Biosecurity Regulators, 2024). Therefore, research institutions, funders, and other stakeholders should establish and empower diverse, interdisciplinary, institutional, and external committees to review research proposals, evaluate potential harm, and determine suitable precautionary measures based on the progressing state of the art potentials and the advances in SynBio and other converging technologies. Research should be halted if its risks outweigh the benefits. Indeed, moratoria have been successfully installed in the past (Bonham, 2022), for instance, Asilomar (Berg et al., 1974), GOF research from 2014 to 2017 (White House Office of Science and Technology Policy, 2014; 2017), Chimera Research and human cloning (National Institutes of Health, 2015), as well as human genome editing (Lander et al., 2019).

To complement these assessment mechanisms, we advocate for the enforcement of minimum cyberbiosecurity standards (Elgabry and Johnson, 2024; George, 2019; Greenbaum, 2023), including established frameworks, alongside mandatory digital signature requirements for gene library transactions to ensure supply chain integrity. In the context of SynBio and converging digital technologies, efforts to integrate cybersecurity principles into biosecurity are still in the early stages. Cyberbiosecurity practices are fragmented and only partially systematized in terms of communication, training, and policy development.

It would be prudent to shift critical production capacities (such as vaccines) into distributed modular networks. This reduces single points of failure and increases response speed. Indeed, the WHO is establishing a network of distributed production capabilities with the mRNA Technology Transfer Hub in South Africa (WHO, 2025). At the same time, the WHO Pandemic Hub is building capacities for improved data and analytics flows, which are crucial for more resilient supply and production chains (WHO, 2024).

Finally, we address the need for developing standardized interdisciplinary terminology and fostering collaborative partnerships across cybersecurity and biosecurity domains to facilitate more effective risk identification and coordinated response strategies (Box 2).

Box 2 **Recommendations for improving biosecurity by implementing robust training and monitoring systems.**

- **Comprehensive training programs:**
  - Develop and mandate conceptual training programs for all personnel in biotech and AI laboratories, incorporating modules on dual-use research, cyberbiosecurity, and ethical decision-making.
  - Regularly update training content to reflect evolving risks, new technologies, and lessons learned from incidents.
- **Holistic monitoring systems:**
  - Implement real-time monitoring and early warning systems for emerging biosecurity threats, leveraging AI for anomaly detection and rapid response.
  - Conduct continuous model audits and risk assessments of AI-driven biotech tools across the entire innovation chain, from material and data access to lab procedures and end-user intent.
- **Red teaming and scenario planning:**
  - Establish red teaming exercises and simulated threat scenarios as standard practice to proactively identify vulnerabilities in bio-labs, supply chains, and data infrastructure.
- **Regional innovation sandboxes:**
  - Create regulated environments for testing high-risk SynBio and AI technologies, allowing for real-world experimentation under strict oversight.
- **Oversight committees for DURC evaluations**
  - Implement oversight committees to assess DURC evaluations in project proposals and grant-seeking research.
- **Cyberbiosecurity standards:**
  - Enforce minimum cyber hygiene standards (e.g., NCSC Cyber Essentials) and implement digital signatures for gene library transactions.
  - Provide organizations with resources and training to enhance their cyberbiosecurity posture.
- **Decentralized manufacturing and resilience:**
  - Shift critical infrastructure (e.g., vaccine production) towards distributed models to reduce single points of failure and increase system resilience.
- **Interdisciplinary lexicon and collaboration:**
  - Develop a shared vocabulary and promote interdisciplinary collaboration to facilitate effective risk identification and response.

#### 3.2.3 Adapt governance to keep pace with advances in SynBio through agile, adaptive and flexible governance measures

Many governance approaches seem to remain trapped in the past. Contemporary governance continues to depend on antiquated regulations for governing a new emerging field of converging technologies (Helbing and Ienca, 2024), which evidently is inadequate to guarantee security in the upcoming decades.

To ensure that regulations remain relevant, a proactive approach is necessary, one that is flexible enough to be quickly modified in response to scientific and technological advancements and emerging threats. It could involve the establishment of dedicated national task forces that monitor SynBio developments and recommend timely updates to biosecurity regulations, serving as a central point of coordination for all biosecurity stakeholders (Box 3).

Box 3 **Recommendations for governance adaptations to keep pace with advances in SynBio.**

- **Agile and adaptive regulatory frameworks:**
  - Establish flexible governance structures that can be rapidly updated in response to technological advances and emerging risks.
  - Incorporate iterative policy review cycles and stakeholder feedback mechanisms to ensure that governance remains relevant and effective.
- **Centralized, regional node-based global governance:**
  - Organize governance around regional coordination platforms or centers (e.g., EU, ASEAN, African Union, Mercosur), each acting as a focal point for their respective areas and interconnected through decentralized information systems for transparent policy exchange.
  - Establish an inter-regional coordination council to align objectives, mediate disputes, and review agreements among the regional nodes.
  - Promote global coordination mechanisms to harmonize policies, share intelligence, and enforce regulations across borders, especially for dual-use and high-risk SynBio applications.
- **Peer accountability and competitive collaboration:**
  - Implement periodic peer reviews among regional hubs to promote transparency and adherence to standards, while encouraging innovation and knowledge transfer.
- **Ambidextrous governance:**
  - Balance close monitoring of high-risk activities with the flexibility needed to foster significant innovation.
- **Learning orientation:**
  - Foster a culture of continuous learning and adaptation, drawing on case studies and best practices from other domains (e.g., public health, climate change, nuclear regulation).

For example, the EU is currently exploring anticipatory governance models such as “regulatory sandboxes” and policy labs, already applied in AI policy, to faster adapt to innovation cycles (Ahern, 2025). The planned EU Biotech Act also proposes streamline and harmonize biotechnology regulation across the EU with integrated risk-based frameworks (EPRS, 2025).

Policymakers should consider implementing a tiered regulatory approach, where different levels of oversight are applied based on the potential risk associated with specific SynBio applications. Engaging stakeholders from various sectors in the governance process can also enhance responsiveness to regulatory measures.

Thorough biosecurity and biorisk management policies require the creation of tools and mechanisms to oversee fundamental and applied life sciences. These should encompass legislation, regulations, standards, guidelines, best practices, ethical codes, and research review procedures, as well as education and training. Governance tools and mechanisms should be complementary, mutually reinforcing, and encompass biosafety, biosecurity, and DU research.

Fostering structured dialogue between regulatory authorities and scientific experts ensures that regulatory frameworks keep pace with the most recent scientific advancements and their potential biothreat implications. Governance structures should be supported by iterative policy reviews and ongoing stakeholder feedback.

Such initiatives may be supported by top-down governance, but will necessitate incentivization, harmonization and communication at the international level. Stakeholders should ensure that institutions’ legal roles and responsibilities for risk assessment, education, training, and internal oversight in biosafety and biosecurity, as well as biorisk management are clearly defined. Research that could pose significant societal risks should be governed with particular care. It is hence crucial to convene diverse stakeholders to assess whether and to what extent life science research and development is potentially harmful. This includes activities ranging from funding to publication or commercialization. Crucially, all stakeholders must participate in discussions on the scope and extent of oversight and regulation, whether at the national or international level.

In instances where top-down governance falls short, it will be necessary for other entities - such as universities, non-profit organizations, and businesses - to utilize their own gatekeeping and oversight abilities to safeguard against malicious individuals.

Moreover, global governance should be centralized and based on regional coordination platforms, such as the EU, ASEAN, the African Union, and Mercosur. These platforms would serve as focal points and be linked through decentralized information systems to facilitate transparent policy exchange.

In addition, an inter-regional coordination council should be established to align objectives, mediate disputes, and regularly review interregional agreements. Equally important, mechanisms must be developed to promote global policy harmonization, cross-border intelligence sharing, and regulatory enforcement, especially for DU and high-risk SynBio applications.

Periodic peer reviews among regional hubs should be introduced to enhance accountability, transparency, and knowledge transfer while fostering healthy competition and collaboration. To minimize the harm through information and attention hazards, publishers ought to advocate for and implement a culture of biosafety, biosecurity and biorisk management within the realm of scientific publishing. Editorial guidelines for the identification, review, and publication of manuscripts with biosecurity or DU concerns and advisory committees with a diverse and interdisciplinary background should be implemented to assess such manuscripts.

Due to the DU nature of SynBio and other emerging and converging technologies, governance must remain ambidextrous, balancing strict monitoring of high-risk activities with the flexibility required to nurture innovative breakthroughs. Unfortunately, biosecurity efforts are complicated by ignorance of SynBio’s ongoing progressive potential and the diverse intentions of involved stakeholders/actors. Therefore, a learning-oriented culture must be fostered in SynBio governance by systematically drawing on case studies and best practices from domains such as public health, climate change, and nuclear regulation to continuously improve oversight and responsiveness.

#### 3.2.4 Strengthening international treaties, and establishing robust verification

Strengthening international treaties, such as the BWC and establishing robust verification is imperative, as advances in SynBio, AI, and digital bio-manufacturing (Park et al., 2024; Udugama et al., 2021) present new challenges that go beyond traditional biological agents. These technologies should be explicitly addressed, for instance by the proposed Science and Technology board currently under negotiation. This highlights the urgent need to adapt the BWC to current scientific realities and threats. Explicitly including enabling technologies, such as gene editing and AI-driven pathogen design, in the currently negotiated verification mechanism might close loopholes and ensure its relevance to contemporary DU risks and scientific developments. Accordingly, the BWC should be modernized and expanded to include regulatory provisions for the emerging biosecurity threat landscape.

The BWC’s lack of a formal, workable verification mechanism is a critical vulnerability that undermines its ability to prevent the development and use of BW as biotechnology continues to evolve. Unlike other arms control treaties, the BWC faces unique challenges due to the DU nature of biological research, accessible materials, and rapid biotechnological advances. Previous negotiation efforts have also failed to reach an agreement on verification protocols due to inherent political and technical complexities. Nevertheless, effective compliance with the BWC requires future-proof verification mechanisms. This would require the implementation of on-site inspections, digital monitoring methods, and the sharing of real-time data. These tools can improve transparency, enable the rapid identification of violations, and bolster international trust in compliance processes (Stiefel et al., 2025). Without such mechanisms, confidence in compliance is limited, and enforcement remains difficult. We therefore advocate a modular, incremental approach to verification that leverages scientific tools, data transparency, and cooperative assessments. Implementing partial verification measures would increase transparency, foster trust among states, and mitigate the risks posed by emerging biotechnologies.

Negotiating binding international agreements that focus on AI-enabled biothreats and DU research is necessary to reinforce global biosecurity. Particularly, GOF research is an issue that should be regulated internationally due to the risks associated with it being carried internationally, such as ePPP (Epstein, 2025; Pannu et al., 2022). These agreements should focus on regulating and overseeing newly emerging technologies, while prioritizing security and ethical standards for global biosecurity. Additionally, fostering international cooperation on research and development in SynBio can help to establish norms and standards that discourage the misuse of these technologies. Non-proliferation efforts should be prioritized, with a focus on preventing the development and dissemination of BW.

Effective biosecurity depends on the coordination of intelligence among international agencies, such as INTERPOL and the WHO. The timely sharing of threat information and risk assessments would enable proactive monitoring and rapid, coordinated responses to global biosecurity emergencies.

Multinational AI-biosecurity task forces consisting of AI and biosecurity expert groups should be established to continuously assess risks, advise on treaty implementation, and coordinate crisis responses. These task forces must stay informed on the latest technological advances to inform policies and best practices.

Hence, adaptive monitoring capabilities to detect traditional BW development and novel AI-assisted bioengineering activities are needed. Successfully monitoring compliance will require a concerted effort by governments and civil society, coupled with international cooperation frameworks that can respond to rapidly evolving technological landscapes. A multinational AI-biosecurity task force operating under a strengthened BWC could coordinate rapid responses to biothreats detected via real-time monitoring systems. These comprehensive governance improvements are essential to maintaining global biosecurity while preserving the benefits of legitimate SynBio and AI research.

We also suggest modular, jurisdiction-specific treaty designs with clear enforcement and dispute resolution pathways that can improve governance flexibility. Modular treaty frameworks enhance biosecurity by enabling flexibility and adaptation to regional jurisdictions. They support the incremental implementation of measures that are tailored to the varying capacities and contexts of different nations. Clear enforcement guidelines, mutual recognition of actions, and built-in dispute resolution mechanisms strengthen governance and ensure compliance amid evolving technological landscapes.

Pooled resource management and international funding can further support verification infrastructure, capacity building, and equitable biosecurity access, ensuring a resilient and adaptive global treaty system. International funding and capacity building should be coordinated to support treaty compliance and a robust verification infrastructure. Providing equitable access to resources ensures that all parties to the treaty, regardless of their capabilities, can implement strong biosafety and biosecurity measures.

These aspects should function synergistically to ensure a future-proofed, practical, and robust international biosecurity regime (Box 4).

Box 4 **| Recommendations to strengthen international treaties and establish robust verification.**

- **Modernize and expand the BWC:**
  - Explicitly address advances in SynBio, AI, and digital bio-manufacturing, e.g., in the Science and Technology board, which is under negotiation.
  - Develop and implement a technically future-proof verification mechanism able to deal with current and future technological challenges, such as gene editing and AI-driven pathogen design.
- **Establish robust verification mechanisms:**
  - Establish a verification protocol possibly including but not limited to on-site inspections, digital monitoring, and real-time data sharing, to ensure compliance with treaty obligations.
- **Binding international agreements:**
  - Negotiate and adopt binding international agreements specifically targeting AI. These shall among others also encompass AI-enabled biothreats and DU, including GOF.
- **Intelligence sharing and coordination:**
  - Facilitate intelligence sharing among international agencies (e.g., INTERPOL, WHO) to monitor and respond to global biosecurity threats.
- **AI-biosecurity task forces:**
  - Establish multinational expert groups to provide regular assessments, coordinate crisis response, and advise on treaty implementation.
  - Make the AI treaty flexible, modular, with clear jurisdictional boundaries, mutual recognition of enforcement actions, and dispute-resolution mechanisms.
- **Pooled resource management:**
  - Coordinate international funding and capacity-building efforts to support treaty compliance, verification infrastructure, and equitable access to biosecurity resources.

### 3.3 An overarching governance framework to mitigate SynBio-Driven biological weapon threats

**FIGURE 5:**
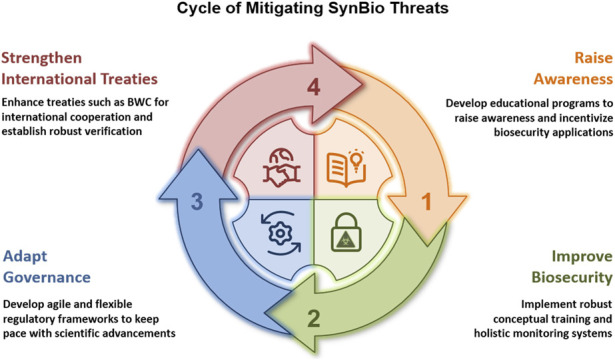
Political recommendations for mitigating SynBio threats include increasing awareness, improving biosecurity training, implementing agile and adaptable governance measures and strengthening international cooperation and treaties, such as the BWC.

By integrating existing and new policy recommendations into a common analytical framework, we outline an overarching, structured governance model for a rapidly evolving, convergent science and technology landscape. We propose a systematic, cyclic approach that continuously identifies, assesses and addresses SynBio-related biorisks and those from converging technologies (Figure 5).

This framework aims first to enhance biosecurity by raising awareness of BWs and the misuse potential of SynBio, through sensitization, education, and incentivization to promote a shared culture of responsibility (Box 1).

Secondly, to improve biosecurity, it is crucial to implement robust conceptual training and holistic monitoring systems that continuously analyze and assess potential risks and novel biological threats (Box 2). These ongoing threat assessments should then lead to the implementation of a culture of biosafety and biosecurity in relevant institutions and the continuous adjustments in governance.

Thirdly, the governance of SynBio faces complex challenges and needs robust, adaptive regulatory frameworks that emphasize flexibility, rapid response, and inclusive stakeholder participation, capable of managing these complexities as they emerge to sustain ethical responsibility while mitigating biosecurity threats (Box 3).

Lastly, although more difficult and slower to implement, it is equally imperative to strongly advocate for the strengthening of international cooperation and treaties such as the BWC to reinforce global non-proliferation efforts. In the face of rapidly advancing scientific and technological developments, we advocate for the evolution of the BWC to ensure its continued relevance. The BWC regime must transition from reactive to proactive and anticipatory governance. This shift would enable the BWC to more effectively address potential threats and uphold BW non-proliferation amid evolving capabilities and novel misuse possibilities (Box 4).

This multi-level biosecurity governance framework emphasizes flexibility and continuous adaptation to enable rapid prevention of and response to accidental or deliberate misuse of SynBio. It combines Top-Down regulation with Bottom-Up engagement (See Table 2 for examples) in polycentric, overlapping jurisdictions across global, national, regional, and local levels, to ensure resilience and context-specific risk management.

This hybrid framework supports real-time information sharing and aligns incentives across actors, to speed threat detection, diffuse best practices, and drive adoption as well as compliance. It combines monitoring tools with ethical guidance, and regularly updated codes of conduct. In addition, it fosters experimentation and policy innovation through pilot projects and regulatory sandboxes to keep pace with innovation (see Zhou et al., 2024) alongside international, cross-sectoral cooperation; multi-stakeholder co-design; layered risk management; and continuous feedback.

## 4 Discussion

This Delphi study first investigated the most pressing factors for potential new biological risks through threat prioritization by experts. Secondly, it generated consensus-based policy recommendations that would enable the establishment of appropriate governance improvements. On this basis, we developed an overarching governance framework to coherently and comprehensively implement these recommendations and thereby mitigate biological threats, especially those involving SynBio-driven BW.

According to our expert panel, the threat landscape of synthetic BW is increasingly shaped by the dual dynamics of “de-skilling” certain scientific tasks and their democratization, as has been previously discussed (Götting et al., 2025; National Academies of Sciences, Engineering, and Medicine, 2025; Peppin et al., 2025; Rose et al., 2024; Soice et al., 2023; Williams et al., 2025). They pinpointed enhanced accessibility as one of three main drivers of this risk, along with rapid scientific and technological advances and declining costs of relevant technologies. Moreover, the experts rated the impact of SynBio and new technologies on biosecurity as moderate to high. They identified simplified handling, accelerated development processes and increased availability of enabling technologies as the most significant contributors to the threat of synthetic BW.

Our study suggests that collectively, these factors reduce entry barriers to advanced techniques, facilitate the faster and more effective design, development, and deployment of (synthetic) BW agents, while potentially enabling a broader spectrum of malicious actors. Consequently, this considerably exacerbates the difficulties associated with effective biosecurity, governance, and non-proliferation endeavors.

In accordance with our findings, recent scientific literature highlighted that rapid advancement and convergence of SynBio, AI, robotics, drones, 3D printing, laboratory automation, and nanotechnology introduce new biosecurity risks, (Brockmann et al., 2019; Endy et al., 2025; National Academies of Sciences, Engineering, and Medicine, 2025; O’Brien and Nelson, 2020; Pannu et al., 2024a). Favaro et al. (2022) showed that emerging technologies like SynBio may be militarily deployable by major powers, such as the U.S., Russia, and China by 2040. This significantly impacts biosecurity management and BW threat governance (Groff-Vindman et al., 2025; Gronvall, 2019; Zeng et al., 2022). Especially AI tools could also increase the potential scale of harm by enabling the creation of novel pathogens with enhanced virulence or transmissibility that surpass what is currently possible with biological knowledge alone (Batalis and Venkatram, 2025; Donaldson, 2024; Mouton et al., 2024; National Academies of Sciences, Engineering, and Medicine, 2025; Tegomoh, 2025).

Whereas timelines remain uncertain, the majority of experts we surveyed would even place synthetic biowarfare agents within the realm of possibilities within the next 10 years. In alignment with these findings, AIxBio Global Forum (2025) concludes that such misuse scenarios could become feasible within a few years if proper safeguards are not implemented. This prospect requires the development of comprehensive policies that consider the technical, social, economic, and political dimensions of disruptive technologies such as SynBio, AI, and other converging technologies (Li et al., 2023; Taeihagh, 2023).

In addition, the Delphi expert panel criticized existing biosafety and biosecurity governance regimes and regulation (designed for a different and older set of technological capabilities) for being fragmentary among national and international bodies and inconsistent across sectors, agencies, and institutions. This lack of harmonization creates policy mismatches and enforcement gaps complicating compliance efforts and impeding coordinated risk mitigation. The literature discusses these drawbacks (Gillum, 2025b) and suggests several governance approaches, such as precautionary (command-and-control, hard law), stewardship (soft law), bottom-up, and laissez-faire (industry-driven). However, their integration or balance remains unclear as summarized by Undheim (2024).

The realm of AI converging with SynBio is a good example of the prevailing governance challenges. A broad consensus exists among global, U.S., and German reviews that current legal framework, including the BWC, United Nations Security Council Resolution 1540, and EU Dual-Use Regulation 2021/821, do not yet adequately address the risks posed by digital bio-design and AI-enabled technologies (Bloomfield et al., 2024; Groff-Vindman et al., 2025; Jakob et al., 2025; Undheim, 2024; Wheeler, 2025).

Moreover, proposed solutions are highly contested. Some scholars and policymakers advocate for the enactment of binding legislation and statutory requirements for model evaluations (Bloomfield et al., 2024; Pannu et al., 2025). Others support the German model of academic self-regulation and warn that excessive regulation could stifle research progress (Jakob et al., 2025). There is also disagreement about the most effective level of governance. Proposals range from amending major international treaties, such as the BWC (Bloomfield et al., 2024; Pannu et al., 2025; Undheim, 2024), to adjustments in national export-control laws (Batalis et al., 2024), to the adoption of industry-led codes of conduct (Baker and Church, 2024).

These differences reflect contrasting national legal traditions, such as the constitutional protection of “freedom of research” in Germany and divergent assessments of the maturity and urgency of the threat. The lack of confirmed cases of misuse further dampens enthusiasm for preventive legislation. Although the existence of legal loopholes is widely recognized, consensus on the optimal remedy remains elusive. In this case, implementing a problem formulation approach (Paoli et al., 2022) into the governance framework, specifically having a process where ‘pathways to harm’ are formulated, may guide resource allocation regarding monitoring and verification.

The interdisciplinary governance discourse reveals a spectrum of evidence qualities that influence policy recommendations. Empirical studies that highlight the potential for abuse, such as pathogenic design using LLMs (Gopal et al., 2023) and nucleic acid sequence screening stress tests (Wittmann et al., 2024) provide actionable insights that shape policy recommendations. Conversely, conceptual reviews (Groff-Vindman et al., 2025; Wheeler, 2025) agree on the types of risk, but lack quantitative substantiation, such as benchmarking, etc. This results in broader, less specific governance proposals.

Notably, a divide persists between U.S. and international proponents of mandatory regulatory measures (Bloomfield et al., 2024; Pannu et al., 2025), who analogize oversight to nuclear strict liability, and German scholars who favor ethical oversight and voluntary awareness-raising frameworks (Jakob et al., 2025). These differences reflect divergent regulatory cultures. Resolving these tensions and generating granular empirical evidence are crucial for developing harmonized, effective governance of AI-enabled SynBio.

Non-governmental organizations and international initiatives, such as IBBIS, have emerged to promote harmonized governance mechanisms. Specifically, progress was achieved in the realm of DNA synthesis screening (Wittmann et al., 2025). However, benchtop DNA synthesizers still pose regulatory problems (Gillum and Moritz, 2025; Simon, 2025). Nevertheless, international collaboration remains incomplete without clear mandates, consistent standards, and enforcement capacities. Cross-border harmonization of regulatory frameworks, verification mechanisms, and biosecurity standards is critical to closing these gaps. However, achieving consensus across diverse political and economic systems presents ongoing challenges (Epstein, 2025; Gillum, 2025a).

In light of these discourses, we strongly believe that besides being comprehensive and harmonized, governance models must shift from a reactive to a prospective and adaptive approach. While each of our aforementioned individual policy recommendations addresses specific shortcomings and threats, our proposed iterative, systematic and cyclic approach represents an adaptive and multi-level governance framework.

Many of our considerations are not entirely new. We listed several recommendations from other authors in Table 2. Indeed, policy recommendations to minimize the risks arising from SynBio research and its convergence with AI and other emerging technologies have been discussed (Breyer et al., 2024; Carter et al., 2023; Chaudhry and Klein, 2024; Endy et al., 2025; Esvelt, 2022; Smith et al., 2024). However, these recommendations frequently overlook the dynamic relationship between these interwoven scientific and technological advances and the necessity for proactive and dynamic governance enhancement.

Our proposed framework not only addresses the complex demands of modern bio- and technology-related risks but could adapt to dynamic threat landscapes by responding to rapid innovation cycles and emergent biosecurity threats. In addition, it combines top-down elements, such as government-driven regulation and oversight, and bottom-up elements, such as local, community, and stakeholder engagement. Accordingly, this framework relies on flexibility, distributed authority, strong communication, and a culture of shared responsibility and inclusivity across all levels of governance. Stakeholder engagement and policy tool diversity are also essential.

Hence, we emphasize the need for increased awareness of biothreats and DU technologies through education, training, and incentives; the introduction of robust conceptual biosecurity training and holistic monitoring systems; and the urgent need for agile and adaptable governance to keep pace with advances in SynBio and other converging technologies.

Moreover, in order to protect biological DURC data, cyberbiosecurity must be advanced by implementing minimum cyber hygiene standards. This approach enables public and private sector stakeholders to develop practical, cost-effective mitigation strategies, strengthening resilience and enabling more effective risk management within biosecurity-relevant operations (Adam and McArthur, 2024; Schabacker et al., 2019).

Public trust in bioscience governance is fragile, especially since high-profile debates around GOF research and pandemic origins have become politicized (Horton, 2025; Mills, 2025). The dissemination of AI-designed pathogens or other advanced biological agents poses information hazards with the potential for misuse in bioterrorism or deliberate harm (Haley and Burrell, 2025). These risks are exacerbated by the democratization and de-skilling facilitated by AI tools, which reduce technical barriers and make DU capabilities more accessible to a broader range of individuals, including those with malicious intent. Wang et al. (2025) demand the establishment of standardized risk assessments for AI-biotechnology tools and setting up global monitoring systems to detect emerging threats.

Governance frameworks often face tension between fostering innovation and ensuring biosecurity (Undheim, 2024). Our approach involves the continuous process of identifying, assessing, and addressing risks associated with the rapidly evolving field of SynBio in combination with other emerging and converging technologies. Furthermore, this comprehensive approach encompasses both the prevention and response elements, with the overarching objective being to mitigate potential harm from both accidental and deliberate misuse of (Syn-)biotechnologies.

Indeed, one of the most promising approaches to managing the complexity of modern biological research is adopting a tiered-risk governance framework. This framework classifies research activities according to their risk level based on the potential consequences and likelihood of DU or biosecurity hazards. Moreover, it allows balancing innovation and biosecurity, and they should be regarded as complementary, not mutually exclusive. Additionally, implemented policy instruments need to be diversified beyond supply-side measures to include demand-side incentives and regulatory sandboxes, as have been suggested by Zhou et al. (2024).

Adaptive governance models fit well within this structure, offering the flexibility to adjust oversight mechanisms as technologies and scientific understanding evolve. Tiered models allow for differentiated review processes, reserving more intensive scrutiny for experiments involving higher-risk outcomes, such as GOF studies on pathogens with pandemic potential. This stratification allows for the more efficient allocation of regulatory resources, reduces unnecessary burdens on low-risk research, and encourages innovation. Examples include implementing tiered Institutional Biosafety Committee (IBC) review protocols and federal funding agency guidelines that align risk tiers with corresponding oversight intensity (Gillum, 2025a; 2025b). AI applications may improve governance by providing predictive analytics, risk assessments, and decision support systems. However, research on integrating these tools responsibly within governance structures is limited (Danchin, 2024).

In view of the transnational nature of biotechnology research and the risks posed by pathogens, there is a clear need for global cooperative governance. We therefore strongly advocate for cooperation and the improvement of non-proliferation efforts through international treaties such as the BWC and establish future-proof verification as a crucial cornerstone. Although scientists worldwide highlight its importance (Forman et al., 2025; López-Vergès et al., 2025; Zakaria et al., 2025), it is clear that this will be the most difficult part of the proposed framework.

## 5 Limitations

The results presented should be considered in light of the limitations of this study. The Delphi method has been criticized for potential “homophily bias,” (Holeman et al., 2024) which we attempted to minimize by increasing participant diversity. For the group workshops, anonymity was waived for the benefit of the expert discussions as is common in Group Delphi processes (Niederberger and Renn, 2019). This may have caused various biases such as framing, anchoring, desirability bias, bandwagon effect, etc. (Winkler and Moser, 2016). We tried to reduce these biases utilizing multiple mixed breakout sessions during the workshops. However, the experts strongly voiced against it, preferring forum discussions.

Although we aimed to recruit participants with an active interest in this field, the study is limited by the willingness of those invited to participate. Since only biosecurity experts from German-speaking countries were included, perspectives of experts from other disciplines or regions were not included. We did not invite experts on the various converging technologies due to concerns how such experts might contribute to discussions on biosecurity. Although in hindsight, an expert on AI, especially BDTs, might have been an asset. While response stability could not be measured, most experts did not substantially change their judgements, despite other experts’ justifications. Discussing policy recommendations and topics of dissent during the group workshop were deemed more important than refining survey consensus, though consensus may have been reached through additional survey rounds.

## 6 Conclusion

In conclusion, addressing the challenges posed by rapid innovation and emerging biosecurity threats requires adaptive, multi-level governance frameworks that combine top-down regulation with bottom-up stakeholder engagement. We recommend the continuous development of this proposed comprehensive governance framework in accordance with the developments in this dynamic field. We acknowledge that its implementation would require significant political change in a context where governance has historically evolved through incremental adjustments. While the optimal entry point for this process is uncertain, incidental reforms will likely yield measurable improvements and may facilitate more far-reaching structural transformations over time. Future work should identify the most qualified governmental bodies and stakeholders, clarify their responsibilities and competencies, as well as the specific methodology for implementing governance changes.

## Data Availability

The original contributions presented in the study are included in the article/Supplementary Material, further inquiries can be directed to the corresponding author.
