# Supplementary material for: Improving governance in the age of synthetic biology, artificial intelligence, and diverging threats
Source: Front Bioeng Biotechnol. 2026 Apr 20;14:1705143. doi: 10.3389/fbioe.2026.1705143 (PMC13136710; doi:10.3389/fbioe.2026.1705143)
Supplement: Supplementary file 1 [file DataSheet1.pdf]

Improving Governance in the Age of Synthetic Biology, Artificial Intelligence, and Diverging Threats

Dunja M. Sabra[1], Johannes L. Frieß[2], Bernd M. Giese[2], Gunnar Jeremias[1]

1: Carl von Weizsäcker-Centre for Science and Peace Studies, Hamburg University, Germany

2: Institute of Safety/Security and Risk Sciences, BOKU University, Vienna, Austria

Supplementary Material

Delphi Expert Panel

The selection of the expert panel took into account the necessary technical expertise, interdisciplinary experience, and demographic diversity to ensure a comprehensive assessment of the risk of abuse. The members have different demographic backgrounds, including different age groups and genders, and come from across the DACh region. The youngest expert among the four women and eight men (Figure 2) was under 30 years old, and the oldest experts were over 70 years old (Figure S1 and S2).

Delphi studies often have high attrition rates (De Loë et al., 2016) and so additional recruitment was conducted using a snowballing method (referrals from participants) (Denscombe, 2021). Out of 25 contacted experts, 13 participated in the interviews. 12 of them took part in the online survey and 11 participated in the online scenario workshop and 10 in the online survey workshop to generate the political recommendations. The interviews and surveys were conducted individually and thus anonymously. Anonymity was waived for the group workshops. In between scheduled interactions, no communication beyond invitations and the dissemination of preparatory material took place between us and the experts.

Results of the Delphi Process

The expert panel is made up of a diverse group of specialists with a wide range of affiliations who are active in various fields of biological and medical sciences, infectious disease epidemiology, diagnostics and bioforensics of dangerous pathogens and biotoxins, biosecurity and high-security laboratories, biosecurity consulting, technology monitoring and technology assessment, risk analysis and damage control, biological reconnaissance and verification, sabotage protection and NBC protection, as well as governance, CBRN arms control and further development of the Biological Weapons Convention.

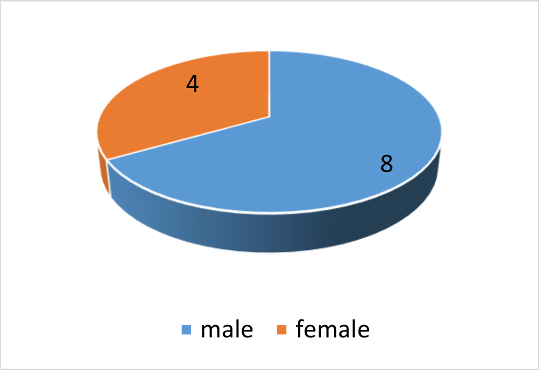

Figure S1: Gender distribution of experts.

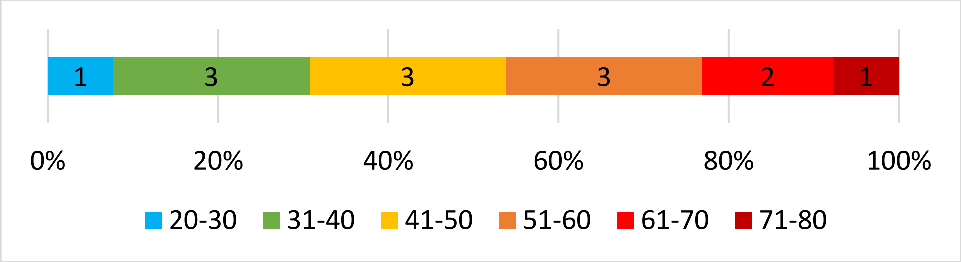

Figure S2: Age distribution of experts.

Interview questions

The structured free-flow interviews were conducted following these questions:

I - Personal information

- Would you please provide your name, gender, and age?
- What is your current position?
- How long have you been working in this field?
- What are your main interests within your area of expertise?
- Is there any other information you would like to share about yourself?

II - Introductory questions

- Do you have experience in the field of biosecurity?
- Which area of biosecurity sparked your interest and what motivated you to participate in this study?
- How do you keep up to date with the latest developments and trends?
- What sources or resources do you use on the topic of biosecurity, i.e. where do you obtain information on biosecurity?
- Have you already carried out projects or activities related to biosecurity?

III – Key questions

a) Technological progress and scenarios

- Which technologies, possibly also in connection with synthetic biology, do you consider problematic in terms of their dual-use potential?
- Which technologies would you consider to be converging with synthetic biology?
- To what extent do these technologies converge with or support the development of synthetic biological warfare agents?
- Where do you see the problem in relation to the production of a(n) (more) effective biological weapon?

- In your view, what are the most likely and most worrying scenarios for biological threats from biological weapons in the next 10 years?
    - Using synthetic biology?
    - And other converging and emerging technology systems?
- What are the main drivers and prerequisites for biological threats from synthetic biological warfare agents?

b) Targets and vulnerabilities

- What are the most vulnerable and resilient targets and sectors to biological threats compared to synthetic biological warfare agents?
- What are the most critical and neglected gaps and challenges in current and future research and development in synthetic biology and biosecurity?
    - And other converging and emerging technology systems

c) Actors

- Which potential actors do you consider to be the most dangerous in relation to synthetic biological warfare agents?
- How do you assess the current and future threats posed by synthetic biological warfare agents?
    - In relation to bioterrorism
    - In relation to armed conflicts

d) Implications

- What are the biggest, most challenging implications (and perhaps also opportunities) of synthetic biology and other converging and emerging technological systems in terms of biosafety?
- How can misuse be prevented?

e) Countermeasures

- Do you see a need for action in the area of biological threats posed by synthetic biological warfare agents?
- What are the practices and strategies for intervening in/containing biological threats posed by synthetic biological warfare agents?
- Which of these approaches do you consider promising or problematic?
- How can readiness for their implementation be promoted?

f) Communication and stakeholder engagement

- How can cooperation and coordination between different stakeholders and sectors be promoted in order to address biosecurity issues?
- How much awareness is there of the biosecurity risks associated with synthetic biologicalwarfare agents? How can this awareness be increased?
- How can individual stakeholders be encouraged to develop appropriate biosecurity measures?
    - How can the public and policymakers be informed about the benefits and risks of synthetic biology and other converging and emerging technology systems for biosecurity, and what form should this communication take?

g) Governance

- How do you rate the effectiveness of current biosecurity policies and regulations?
- Is current legislation on biosecurity sufficient?
- If not, how could the existing legal framework be adapted to meet both the demands of technological innovation and societal safety concerns?
- How do you assess the current regulation and monitoring of biotechnologies to prevent biological hazards?
    - Do you have any suggestions for improvement?

III – Final question

- What doomsday scenarios can you imagine?
- How realistic do you think such a scenario is?
- What needs to be done today to prevent this scenario from happening?
- In your opinion, have we forgotten any questions?

Survey results

Below the results of the online surveys are discussed in detail. The results of the first survey are compared directly with those of the second survey round. In both surveys, twelve experts participated.

1. Prior Knowledge

1.1 How comprehensive would you say is your prior knowledge of the following topics?

The answers chosen by the experts are summarized in Figure S3.

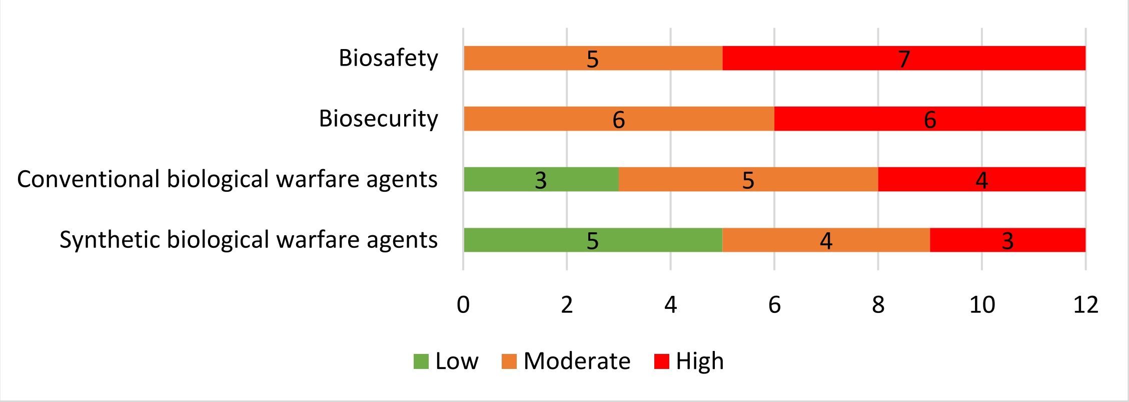

Figure S3: Stacked bar chart of responses to question 1.1, of the first round of surveys

The expert panel considers its prior knowledge to be greater in the field of biosecurity than in the field of biological weapons. This becomes even clearer when looking at the ranking in Figure S4. As expected, knowledge in the field of synthetic biowarfare agents is even lower than that of conventional biowarfare agents. Since prior knowledge would not change between the two online surveys, this question was removed from the second questionnaire.

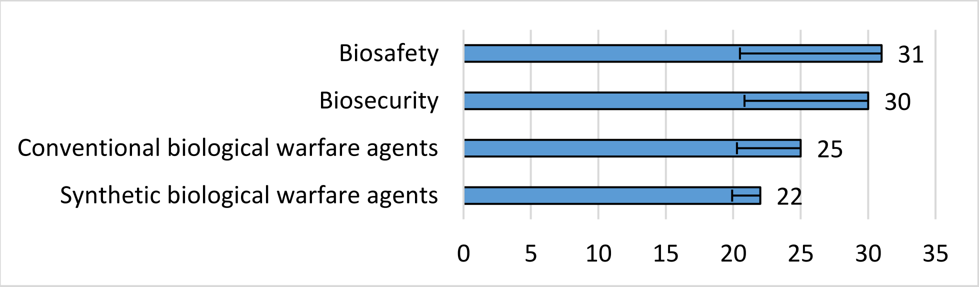

Figure S4: Scoring and ranking of the answers given to question 1.1 of the first round of surveys. Error bars represent the standard deviation depicted only in one direction for clarity.

2. Threat perception

2.1 How concerned are you about the following biological threats? Please rank them as follows: 1st position = most concerned, 5th position = least concerned.

In the first online survey, the experts responded as shown in Figure S5.

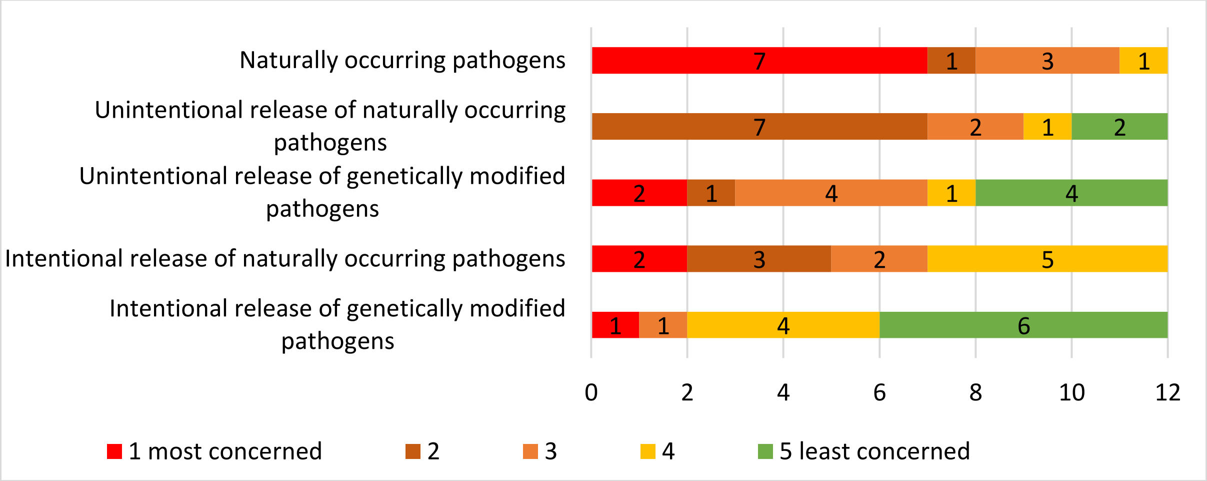

Figure S5: Stacked bar chart showing the responses to question 2.1 of the first online survey.

Most experts (7) say they are most concerned about naturally occurring pathogens. The same number of experts rank the unintentional release of a pathogen second. Six experts are least concerned about the intentional release of a genetically modified pathogen. No consensus could be found on any item. While strong tendential consensus were observed with regard to the intentional release of a genetically modified pathogen and the unintentional release of a pathogen, there were weak tendential consensus regarding unintentional releases of genetically modified pathogens. A ranking is shown in Figure S6.

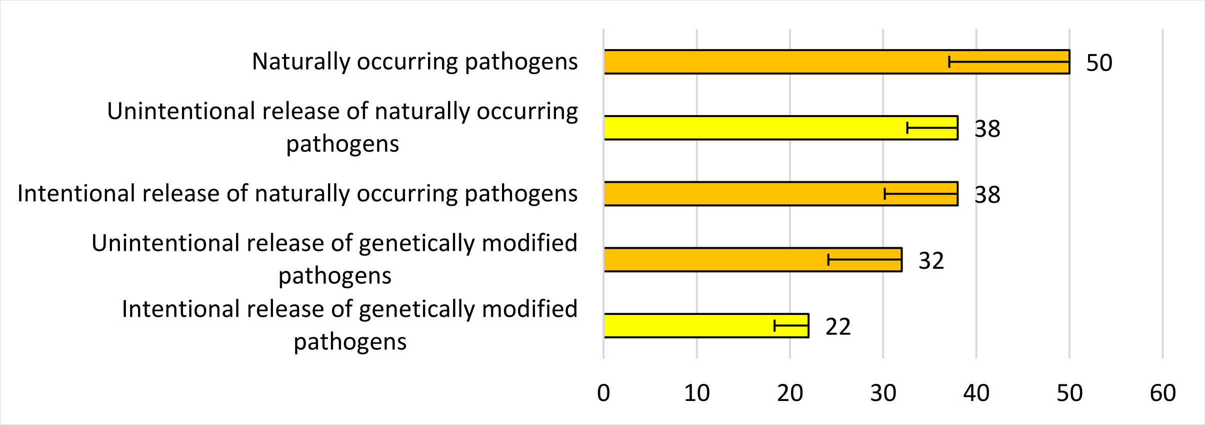

Figure S6: Ranking of responses to question 2.1 of the first survey round. A high score corresponds to greater concern. Yellow bars indicate strong tendential consensus and orange bars indicate weak tendential consensus. Error bars represent the standard deviation depicted only in one direction for clarity.

Experts are most concerned about naturally occurring pathogens, while the release of naturally occurring pathogens, whether intentional or unintentional, is considered to be balanced. Experts are less concerned about the unintentional release of a genetically modified pathogen and are least concerned about the intentional release of such a pathogen. The responses to the second online survey are shown in Figure S7. There was consensus regarding naturally occurring pathogens.

However, there was strong disagreement regarding the intentional release of genetically modified pathogens, with four and six responses at opposite ends of the scale.

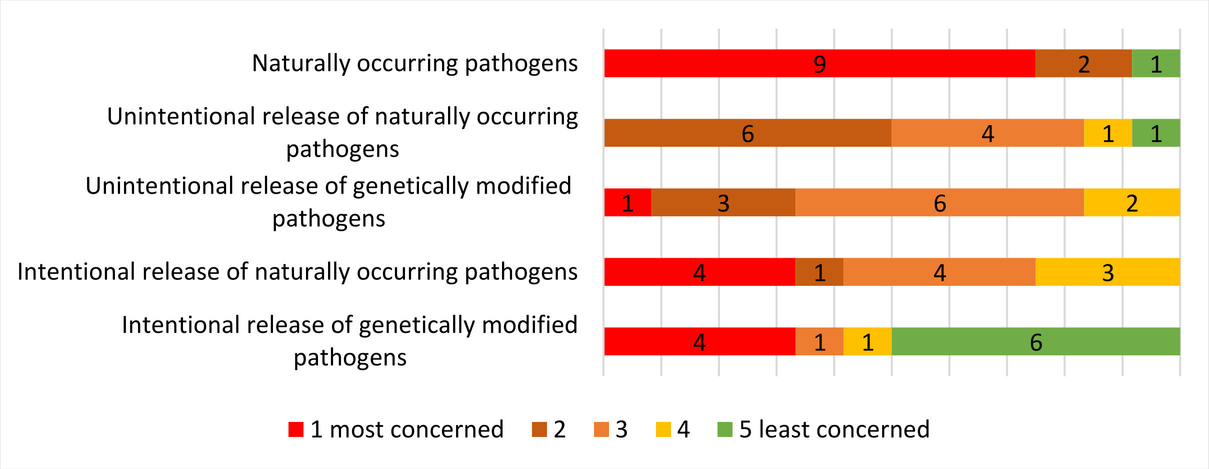

Figure S7: Stacked bar chart of responses to question 2.1 of the second online survey.

A ranking of the responses received in the second round of the survey is shown in Figure S8. This shows that the response “naturally occurring pathogens” has now received 4 more ranking points. In addition, a consensus has been reached. While the intentional release of naturally occurring pathogens has now become more worrying, albeit with dissent, than the unintentional release. However, there is now no difference in concern between unintentional releases of genetically modified or naturally occurring pathogens. Experts remain least concerned about the intentional release of genetically modified pathogens, although this value is also associated with dissent.

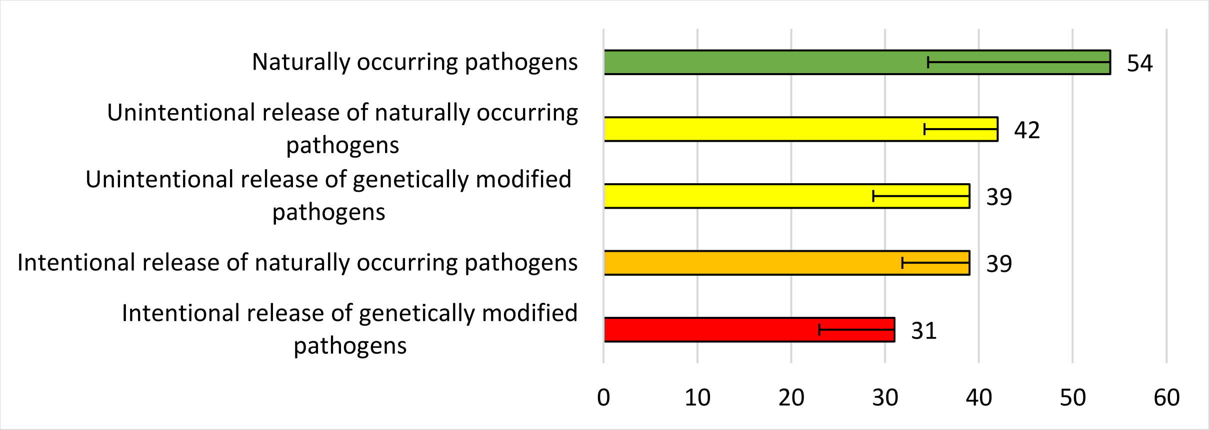

Figure S8: Ranking of responses to question 2.1 of the second round of the survey. A high score corresponds to greater concern. Green bars represent consensus. Yellow bars indicate strong tendential consensus and orange bars indicate weak tendential consensus. Red bars indicate dissent. Error bars represent the standard deviation depicted only in one direction for clarity.

2.2 Do you believe that there is sufficient awareness among experts and the scientific community of the potential risks posed by the following biological threats?

As shown in Figure S9, the experts responded as follows in the first round of the survey.

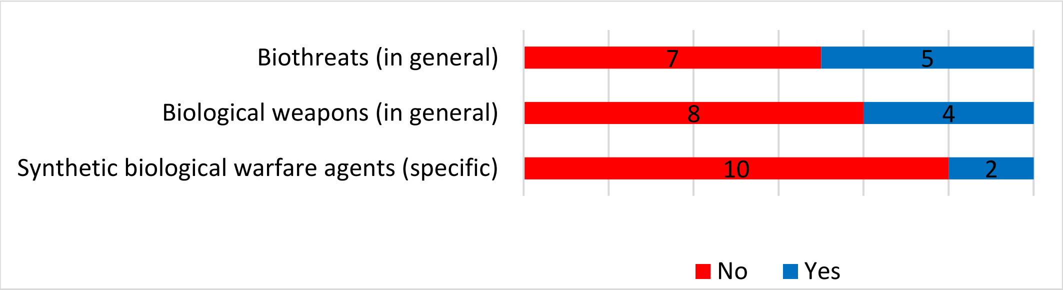

Figure S9: Stacked bar chart showing the responses to question 2.2 of the first online survey.

With regard to synthetic biological warfare agents in particular, there was a clear 10:2 consensus among the experts that awareness of potential risks among specialists and in the scientific community is insufficient. This item was therefore removed from the questionnaire for the second round of online surveys. In general, the majority of experts consider awareness of all three items mentioned to be insufficient. The responses from the second round of the survey are shown in Figure S10.

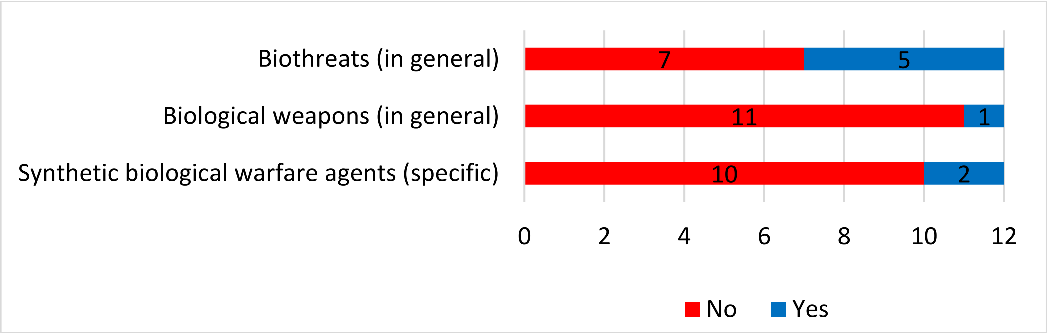

Figure S10: Stacked bar chart showing the responses to question 2.2 of the second online survey.

In the second survey round, there was only one change regarding biological weapons in general. The experts now reached a clear consensus that awareness of the potential risks of biological weapons in general is insufficient among experts and the scientific community.

2.3 In your opinion, when could synthetic biological warfare agents become a more relevant threat than conventional biological weapons?

The results of the first survey round are shown in Figure S11. Here, a very heterogeneous picture emerged, with each answer being selected by at least two experts. This changed dramatically in the second survey round, as shown in Figure S12.

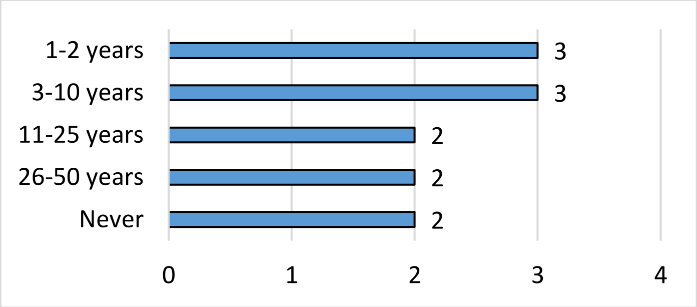

Figure S11: Bar chart showing the responses to question 2.3 of the first survey round.

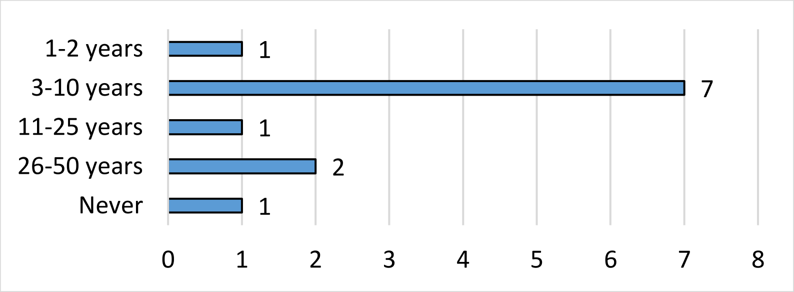

Figure S12: Bar chart showing the responses to question 2.3 of the second survey round.

At least four experts changed their opinion to the effect that, after the second survey round, a slight majority of the expert panel believed that synthetic biological warfare agents could become a more relevant threat than conventional biological weapons within the next 3–10 years.

2.4 How do you assess the following potential drivers in relation to biosecurity or the biological threat posed by synthetic biological warfare agents?

Figure S13 shows the results of the first survey round. After the first round, dissent was observed with regard to the items “Increased accessibility for a broader public”, “Lower costs of technologies”, “Existing or increasing ideological motivation”, and “Few or inadequate control options. Weak tendential consensus was obtained for “Technological and scientific progress”, “Declining tacit knowledge requirements,” “Open Access data/databases,” and “Greater availability of technologies.” In addition, strong tendential consensus emerged in the items “Geopolitical developments and tensions” and “Simplified misuse of technology and science”.

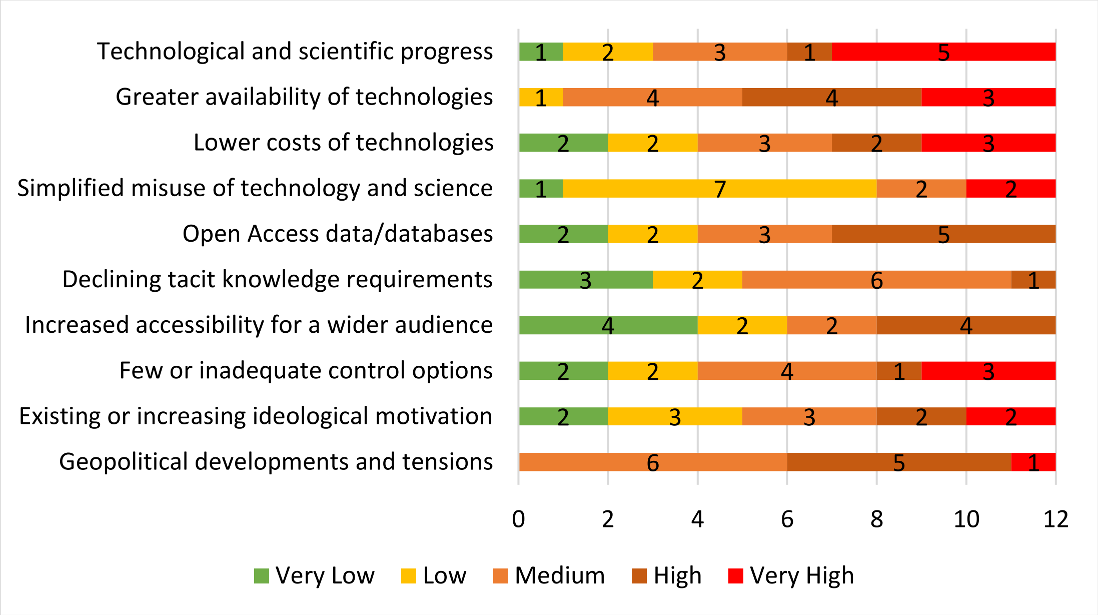

Figure S13: Stacked bar chart showing the responses to question 2.4 of the first survey round.

These results were used to create a ranking of the driving forces related to biosecurity and the biological threat posed by synthetic biological warfare agents (Figure S14). The tendential consensus described above is also highlighted in color. The experts rated "Greater availability of technologies," "Technological and scientific progress," and "Geopolitical developments and tensions" as the three most important of the driving forces mentioned. "Declining tacit knowledge requirements" was rated as the least important. The results of the second round of surveys are shown in Figure S15.

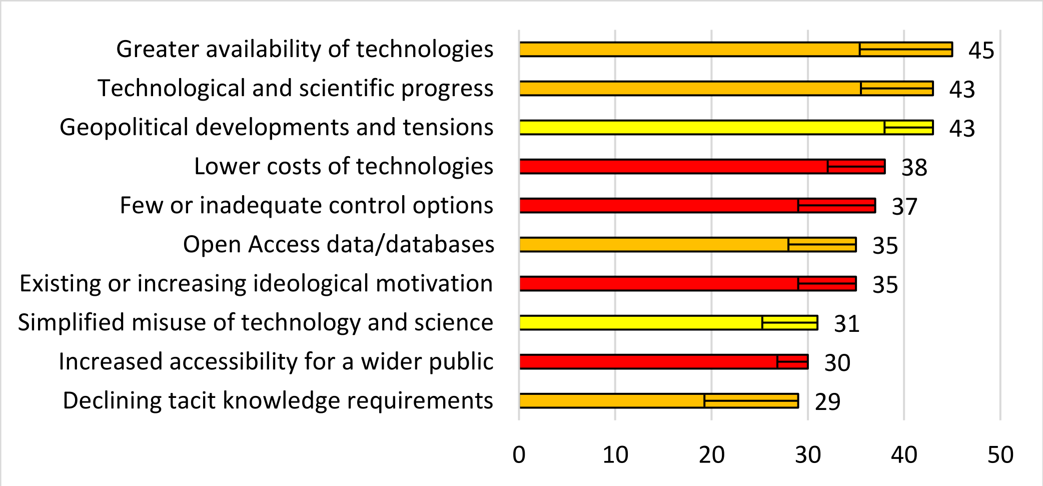

Figure S14: Ranking of responses to question 2.4 of the first round of the survey. Yellow bars indicate strong tendential consensus and orange bars indicate weak tendential consensus. Red bars indicate dissent. Error bars represent the standard deviation depicted only in one direction for clarity.

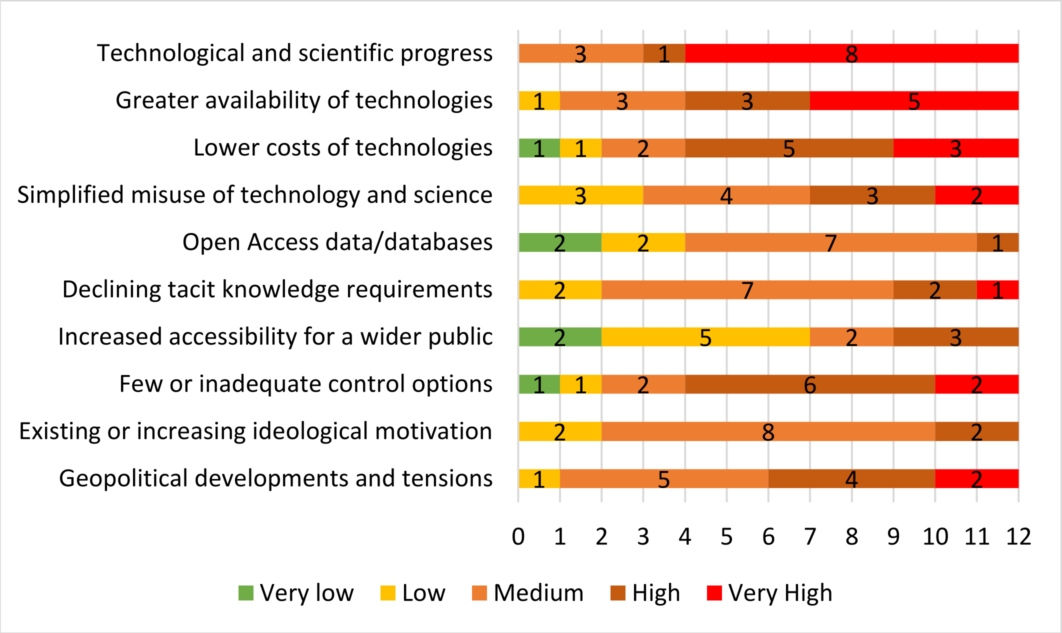

Figure S15: Stacked bar chart showing the responses to question 2.4 of the second survey round.

The second round of surveys revealed significantly more tendential consensus. Strong tendential consensus emerged in relation to the items "Technological and scientific progress," "Geopolitical developments and tensions," "Declining tacit knowledge requirements," "Existing or increasing ideological motivation," and "Open Access data/databases." Weak tendential consensus emerged in "Lower costs of technologies," "Few or inadequate control options," "Simplified misuse of technology and science," and "Increased accessibility for a broader audience." A ranking of these results is shown in Figure 19. The ranking has changed significantly between the two survey rounds. In the second round, the items "Technological and scientific progress," "Greater availability of technologies," and "Lower costs of technologies" were rated as the most important drivers, while this time "Increased accessibility for a broader mass" was rated as the least important.

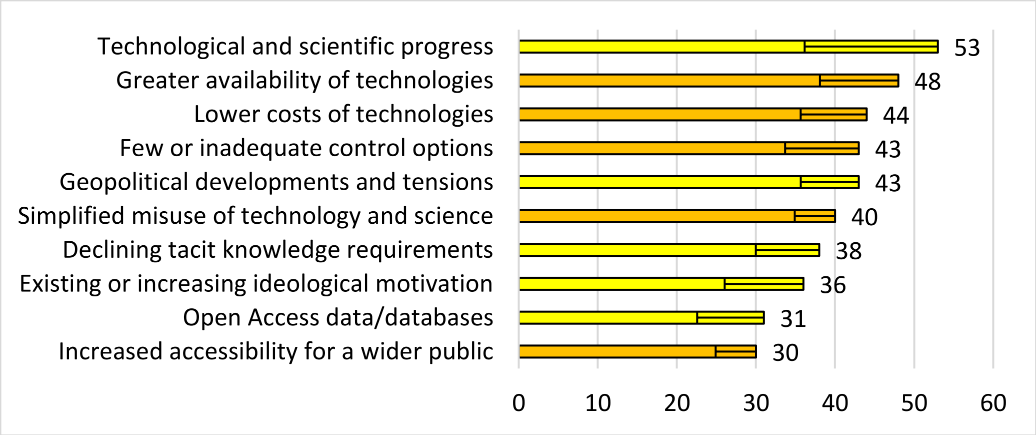

Figure S16:Ranking of responses to question 2.4 of the second survey round. Yellow bars indicate strong tendential consensus and orange bars indicate weak tendential consensus. Error bars represent the standard deviation depicted only in one direction for clarity.

3. Technological and scientific advances

3.1 The pace of technological progress and many current developments in synthetic biology have a major impact on biosafety. In your opinion, how do the following developments influence a potential biological threat from synthetic biological warfare agents?

The results of the first survey round are shown in Figure S17. The responses showed strong tendential consensus in the items “Increased accessibility for a broader audience”, “Accelerated development”, “Accelerated processes through automation”, and “Greater availability”. Weak tendential consensus was seen in the items “Simplified handling of technologies” and “Decreasing costs.” These tendential consensus and the ranking of the items are shown in Figure S18.

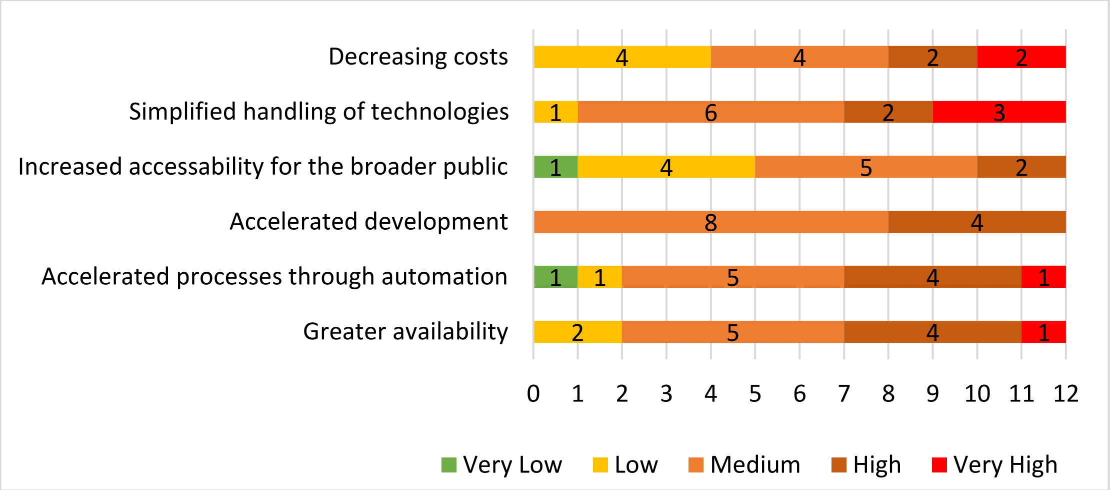

Figure S17: Stacked bar chart showing the responses to question 3.1 of the first survey round.

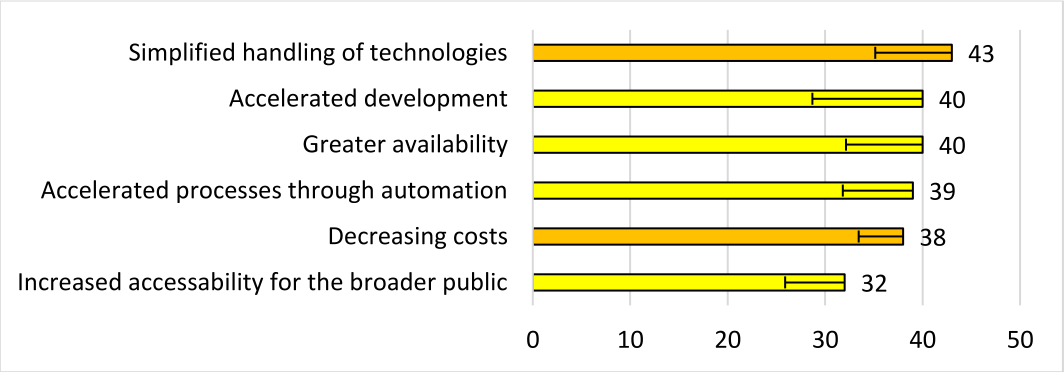

Figure S18: Ranking of responses to question 3.1 of the first survey round. Higher values indicate greater influence. Yellow bars indicate strong tendential consensus and orange bars indicate weak tendential consensus. Error bars represent the standard deviation depicted only in one direction for clarity.

In the second survey round, the tendential consensus for the items “Declining costs” and “Simplified handling of technology” strengthened, while the tendential consensus for “Accelerated processes through automation” and “Increased accessibility for a broader public” weakened. The results are shown in Figure 22. The tendential consensus and the ranking of the responses are shown in Figure 23.

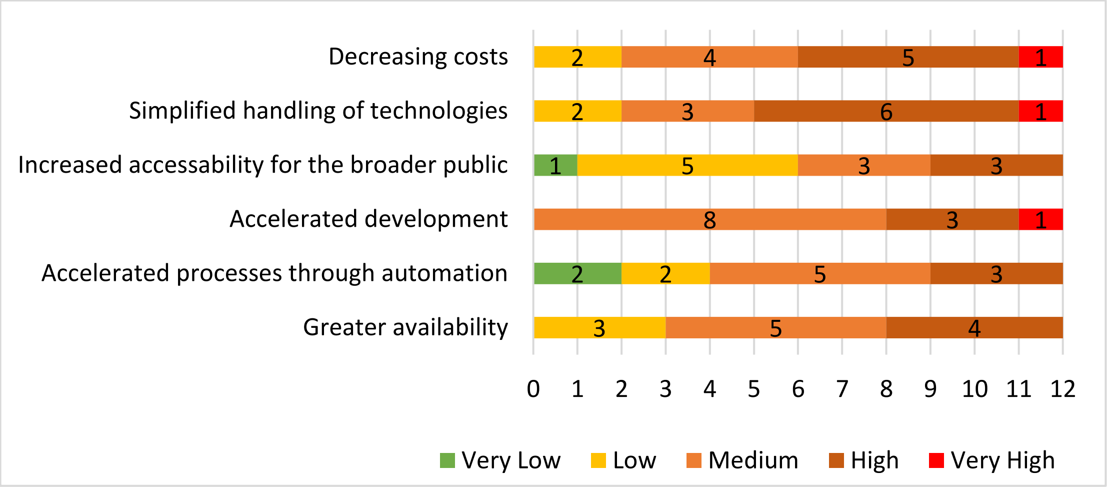

Figure S19: Stacked bar chart showing the responses to question 3.1 of the second survey round.

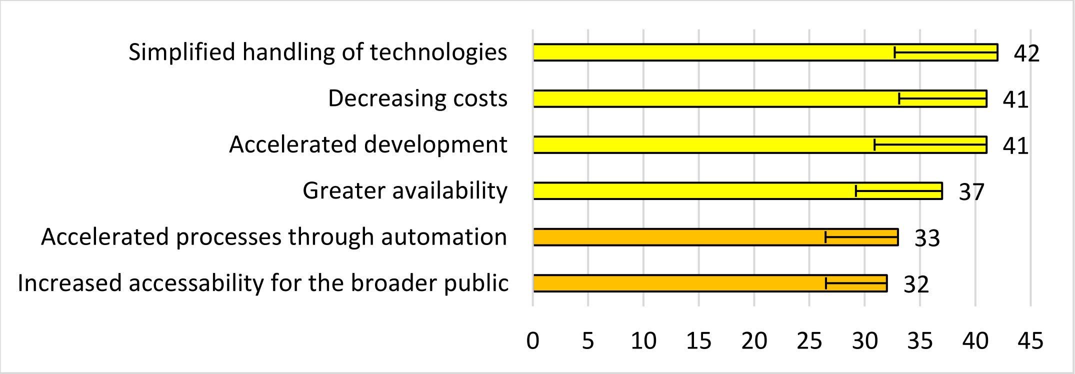

Figure S20: Ranking of responses to question 3.1 of the second survey round. Higher values indicate greater influence. Yellow bars indicate strong tendential consensus and orange bars indicate weak tendential consensus. Error bars represent the standard deviation depicted only in one direction for clarity.

3.2 In your opinion, how significant are the following advances in biotechnology, identified in the expert interviews, for a potential biosafety scenario involving synthetic biological weapons in the next 10 years?

The results of the first round of surveys are shown in Figure S21. Consensus was reached on “Artificial organelles.” Accordingly, this item was removed from the following round of surveys. Strong tendential consensus was observed in the items “Next-generation sequencing”, “Gene drives”, and “HEGAA (Insect Allies).” Meanwhile, the items “Gain-of-function research”, “Targeted drug delivery,” “Bench-top DNA synthesizers”, “Modular biology (kit for everyone/everything)”, “CRISPR/Cas”, “De novo synthesis of nucleic acids”, “Genetically modified bacteriophages”, and “Synthetic bioregulators” showed weak tendential consensus.

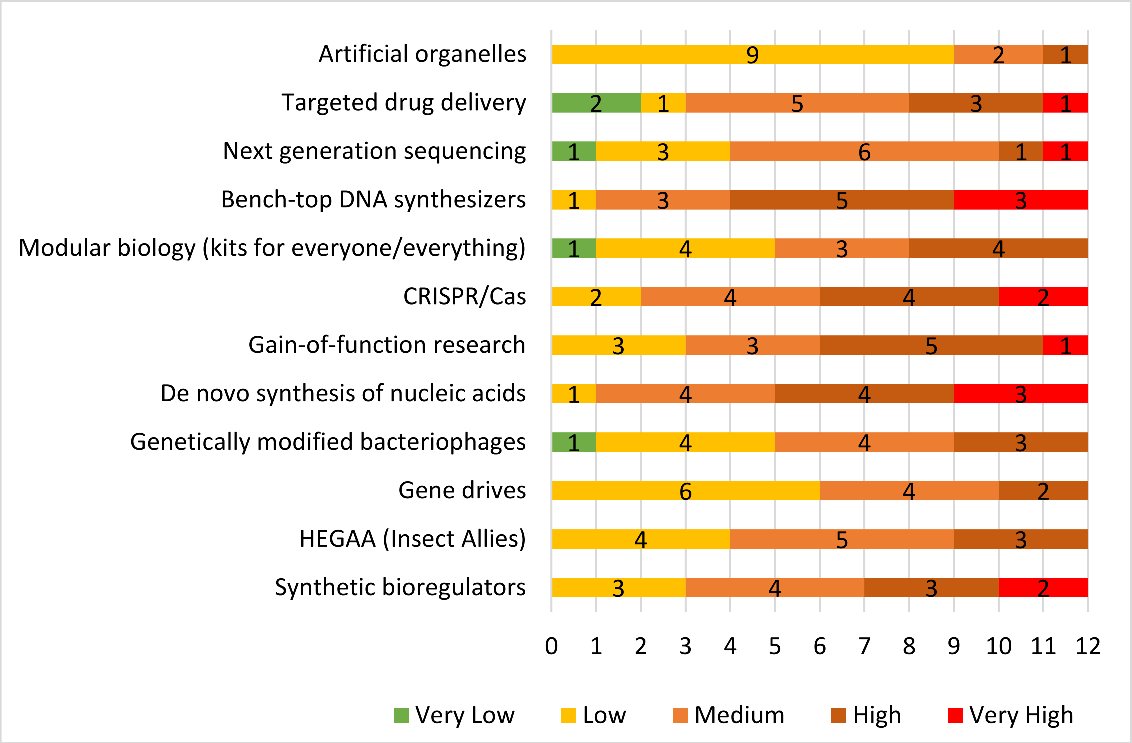

Figure S21: Stacked bar chart of responses to question 3.2 of the first survey round.

Figure S22 shows the ranking of responses from the first round of the survey. The experts rated “Bench-top DNA synthesizers,” “De novo synthesis of nucleic acids,” and “CRISPR/Cas” as the most significant advances in biotechnology for a potential biosafety scenario involving synthetic biological warfare agents over the next 10 years. “Artificial organelles,” on the other hand, was rated as the least significant.

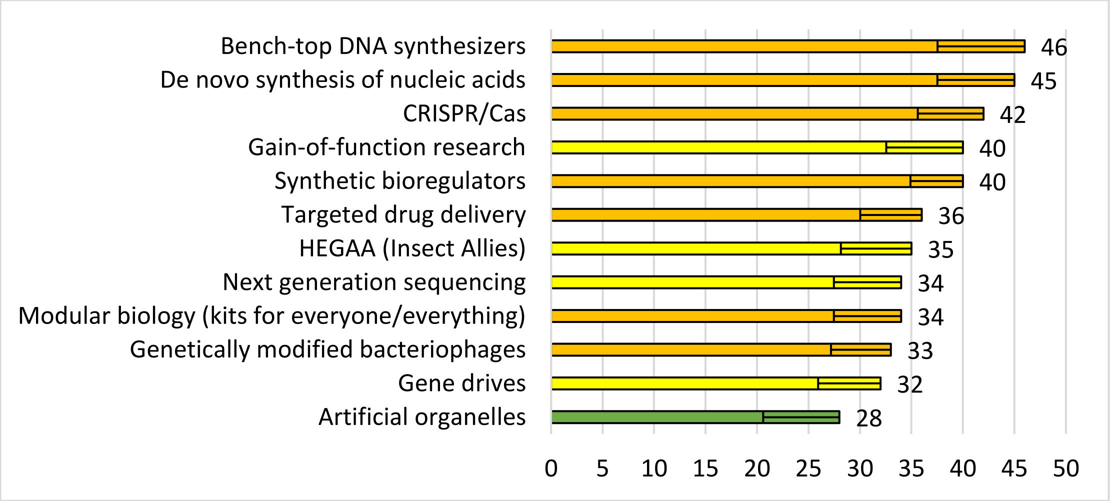

Figure S22: Ranking of responses to question 3.2 of the first survey round. Higher values indicate greater influence. Green bars indicate consensus. Yellow bars indicate strong tendential consensus and orange bars indicate weak tendential consensus. Error bars represent the standard deviation depicted only in one direction for clarity.

The results of the second survey round are shown in Figure S23. The items “Bench-top DNA synthesizers”, “Genetically modified bacteriophages”, “HEGAA (Insect Allies)”, and “Gene drives” show strong tendential consensus. The other items show weak tendential consensus.

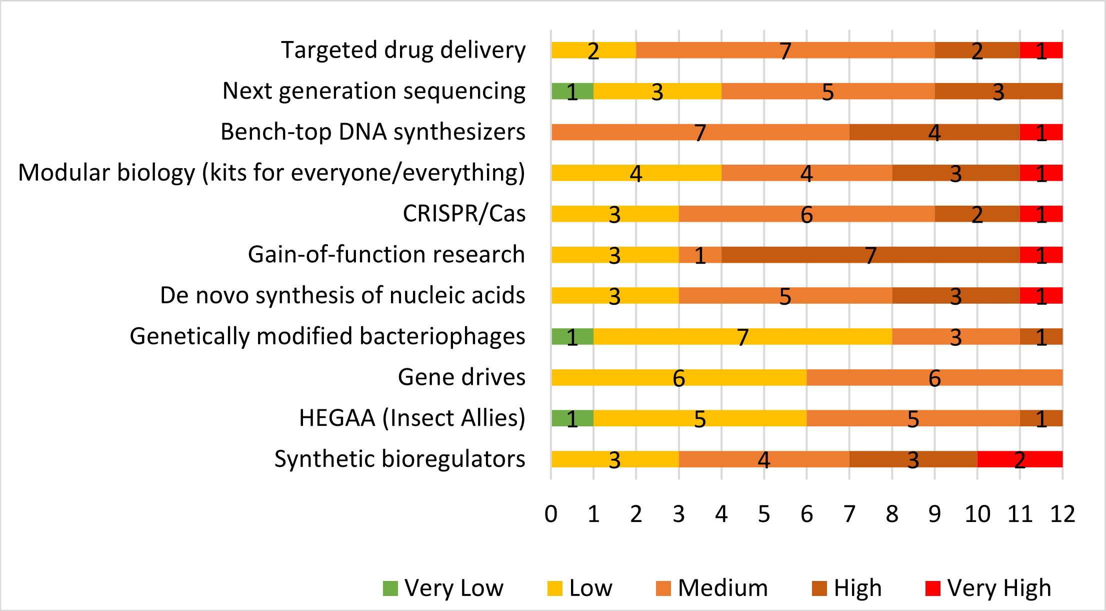

Figure S23: Stacked bar chart showing the responses to question 3.2 of the second survey round.

In the second survey round, the experts rated the items “Gain-of-function research”, “Bench-top DNA synthesizers”, and “Synthetic bioregulators” as the three most significant advances in biotechnology for a potential biosafety scenario involving synthetic biological warfare agents in the next 10 years. This time, “Artificial organelles” were rated as the least significant.

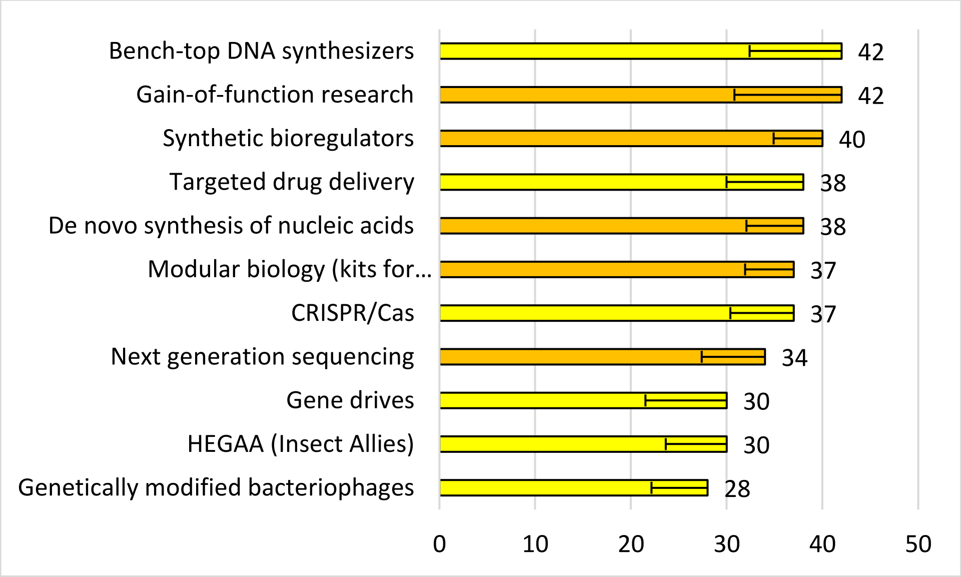

Figure S24: Ranking of responses to question 3.2 of the second survey round. Higher values indicate greater influence. Yellow bars indicate strong tendential consensus and orange bars indicate weak tendential consensus. Error bars represent the standard deviation depicted only in one direction for clarity.

The results of the second survey round are shown in Figure S23. The items “Bench-top DNA synthesizers”, “Genetically modified bacteriophages”, “HEGAA (Insect Allies)”, and “Gene drives” show strong tendential consensus. The other items show weak tendential consensus.

3.3 In your opinion, how significant are the following advances in converging technologies, identified in the expert interviews, for a potential biosecurity scenario involving synthetic biological warfare agents in the next 10 years?

The results of the first survey round are shown in Figure 28. After the first survey, there was no consensus but weak tendential consensus in the items "Cloud Labs" and "AI-modified protein design and AlphaFold." Strong tendential consensus emerged in all other items.

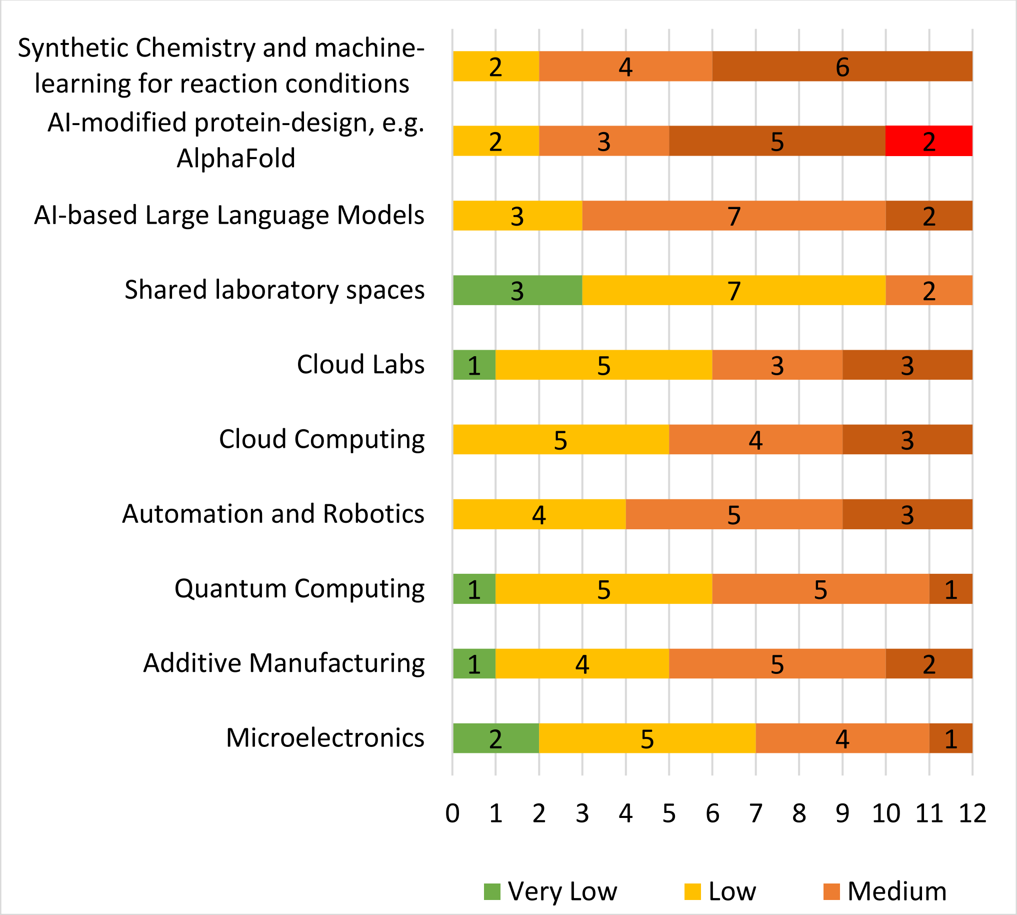

Figure S25: Stacked bar chart of responses to question 3.3 of the first survey round.

The ranking of items according to the experts' assessments is shown in Figure S26. The assessments from the first round of the survey rank "AI-modified protein design, e.g. AlphaFold," "Synthetic Chemistry and machine learning for reaction conditions" as the most significant converging technologies for a potential biosecurity scenario involving synthetic biological weapons in the next 10 years, while "AI-based Large Language Models" and "Automation and Robotics" share the third place. "Shared laboratory spaces" were rated as the least significant.

The results of the second round of surveys are shown in Figure S27. Even after the second round, no consensus was reached. However, the trend toward "AI-modified protein design, e.g. AlphaFold" strengthened. On the other hand, the tendential consensus toward "quantum computing" and "automation and robotics" weakened. Finally, "AI-based large language models" completely lost its previous strong trend. The ranking of converging technologies in the second round of the survey is shown in Figure S28.

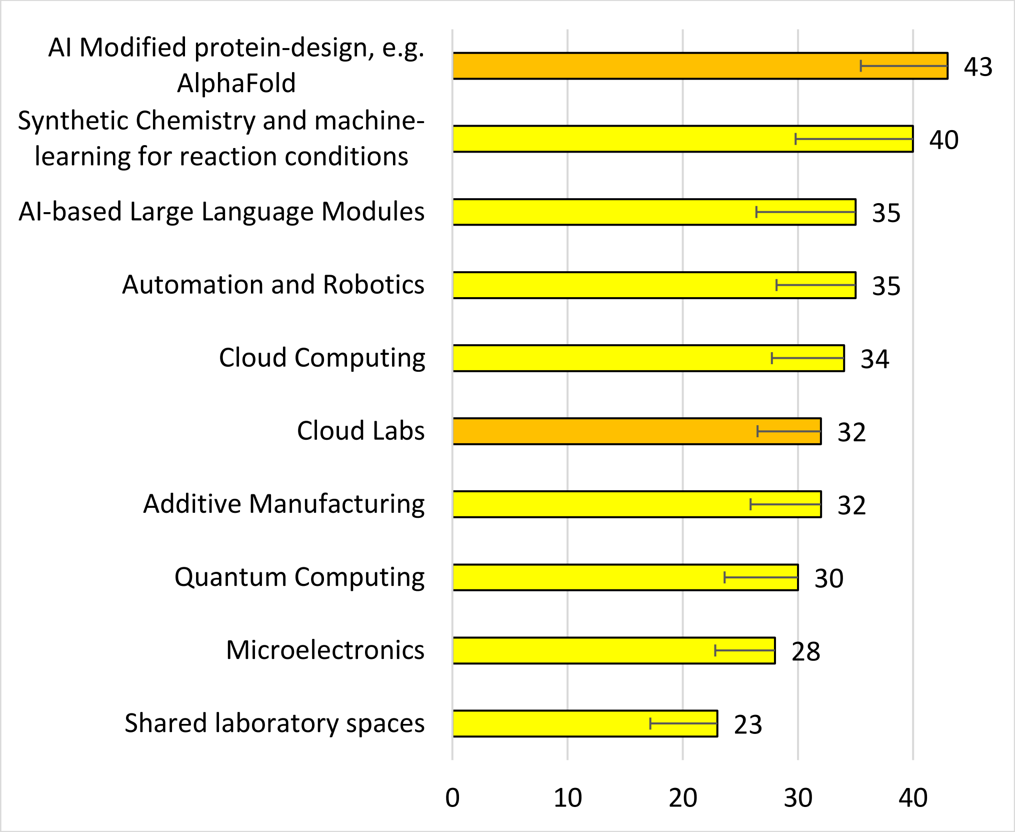

Figure S26: Ranking of responses to question 3.3 of the first survey round. Higher values indicate greater influence. Yellow bars indicate strong tendential consensus and orange bars indicate weak tendential consensus. Error bars represent the standard deviation depicted only in one direction for clarity.

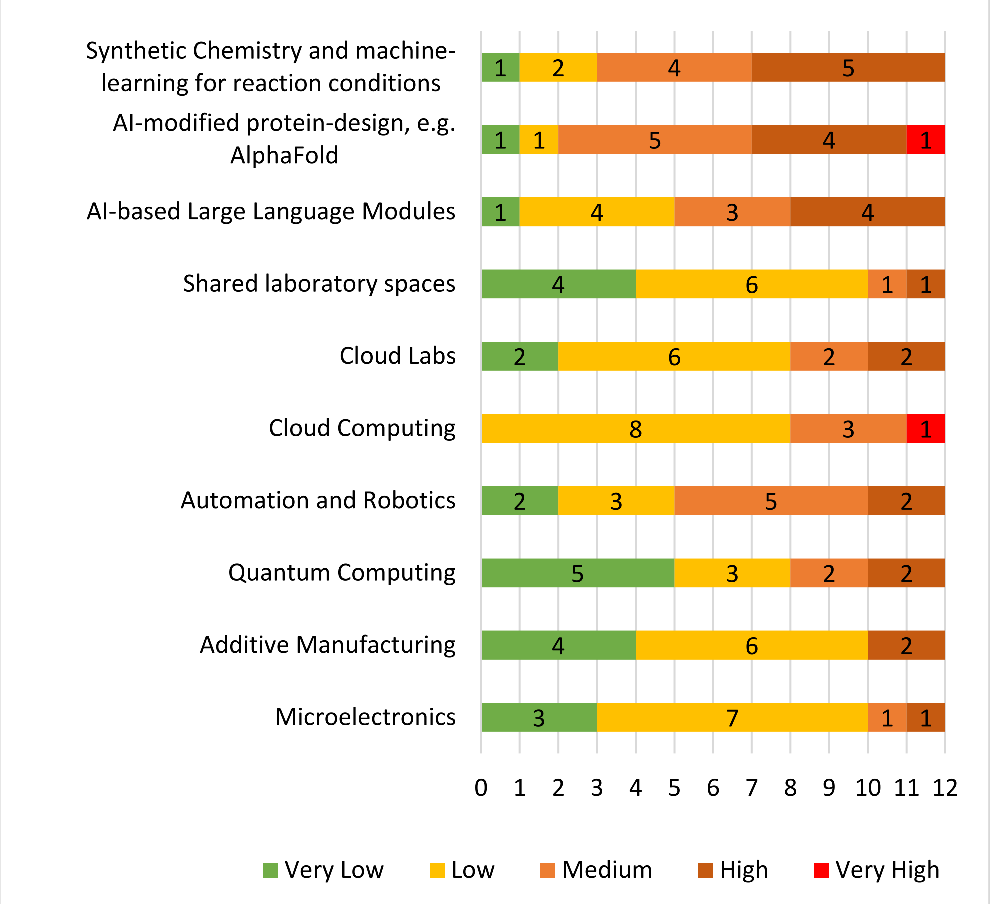

Figure S27: Stacked bar chart showing the responses to question 3.3 of the second survey round.

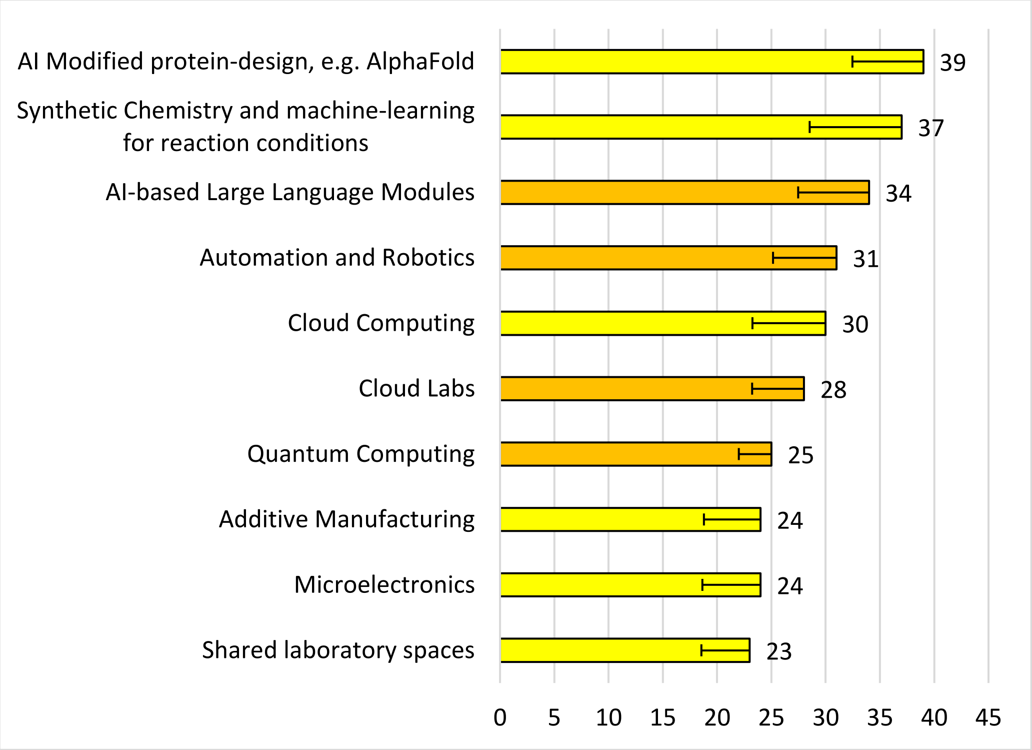

Figure S28: Ranking of responses to question 3.3 of the second survey round. Higher values indicate greater influence. Yellow bars indicate strong tendential consensus and orange bars indicate weak tendential consensus. Error bars represent the standard deviation depicted only in one direction for clarity.

3.4 In your opinion, what are the most likely and most concerning potential uses of biological threats from bioweapons over the next 10 years?

The results for question 3.4 of the first survey round are shown in Figure S29. The use of conventional biological weapons, whether enhanced by converging technologies or not, was rated as moderately to most concerning. The use of synthetic biological warfare agents was rated moderately concerning. There was clear disagreement regarding the “Use of synthetic biological warfare agents enhanced by converging technologies”.

The ranking of the assessments is shown in Figure S30. The assessments in the first round rank the “Use of conventional biological weapons enhanced by converging technologies”, followed by the “Use of conventional biological weapons”, followed by the “Use of synthetic biological warfare agents enhanced by converging technologies”, and finally the “Use of synthetic biological warfare agents”.

The results of the second survey round are shown in Figure S31. The use of conventional biological weapons was rated as most significant. The disagreement regarding the “Use of synthetic biological weapons enhanced by converging technologies” has been resolved. Figure S32 shows the ranking of the responses. The ranking has changed in that the “Use of conventional biological weapons” is now considered the most significant, followed by conventional biological weapons with converging technology, on par with synthetic biological warfare agents, and finally, synthetic biological warfare agents with converging technologies.

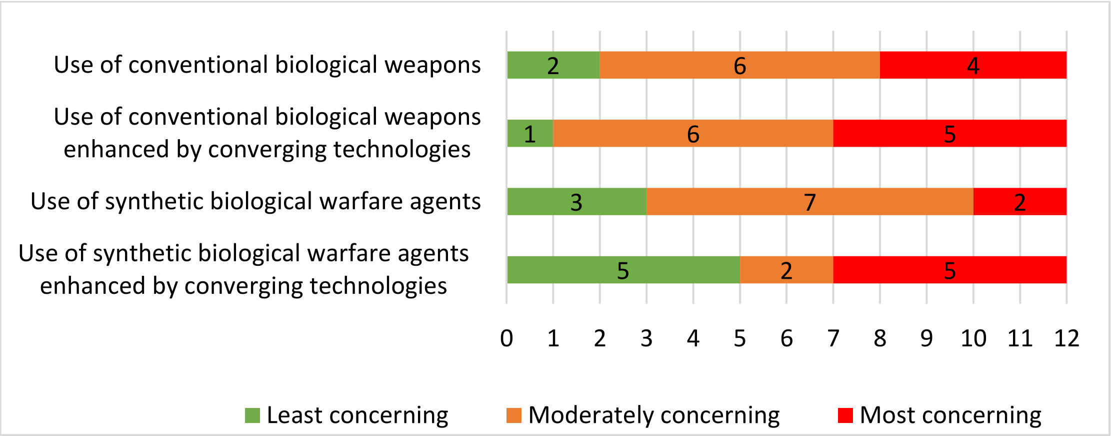

Figure S29: Stacked bar chart showing the responses to question 3.4 of the first survey round.

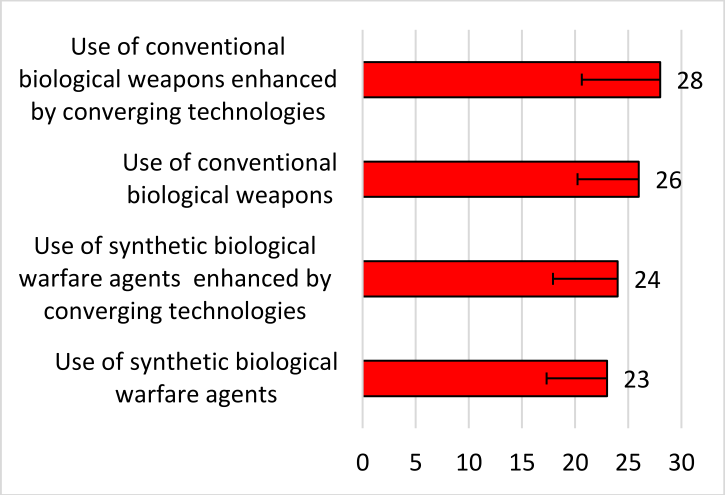

Figure S30: Ranking of responses to question 3.4 from the first survey round. Higher values indicate greater influence. Green bars indicate consensus. Red bars indicate dissent. Error bars represent the standard deviation depicted only in one direction for clarity.

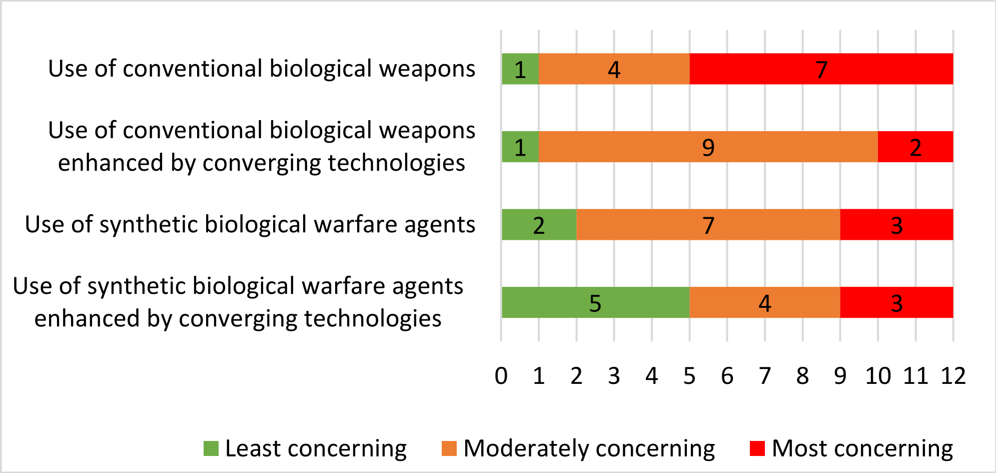

Figure S31: Stacked bar chart showing the responses to question 3.4 of the second survey round.

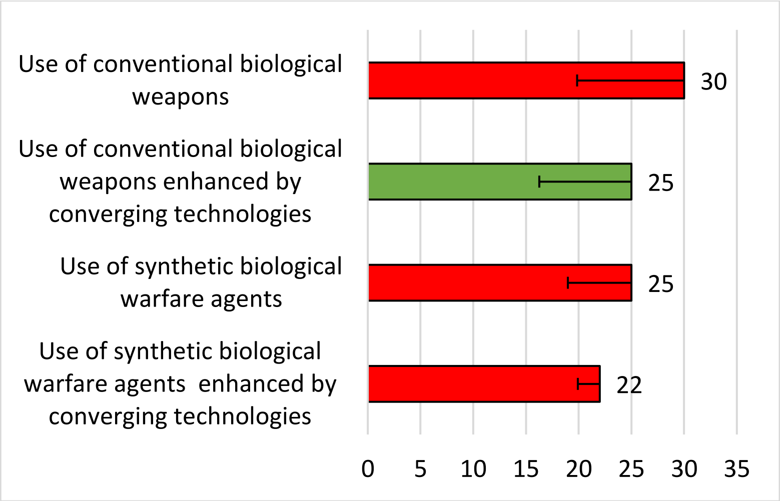

Figure S32: Ranking of responses to question 3.4 of the second survey round. Higher values indicate greater influence. Green bars indicate consensus. Red bars indicate dissent. Error bars represent the standard deviation depicted only in one direction for clarity.

4. Security vulnerabilities

4.1 In your opinion, which vulnerabilities are relevant and could facilitate misuse for the production of synthetic biological warfare agents and weapons?

The results for question 4.1 are shown in order of ranking in Figure S33. Consensus was reached on the items “Lack of risk awareness/sensitivity among (life) scientists” and “Illegal procurement of agents/technology from less regulated countries.”

The results from the second survey round are shown in order of ranking in Figure S34. In the second round, additional consensus was reached regarding “Insufficient surveillance of access authorization to high-security laboratories” and “Illegal procurement of agents/technology from less regulated countries”.

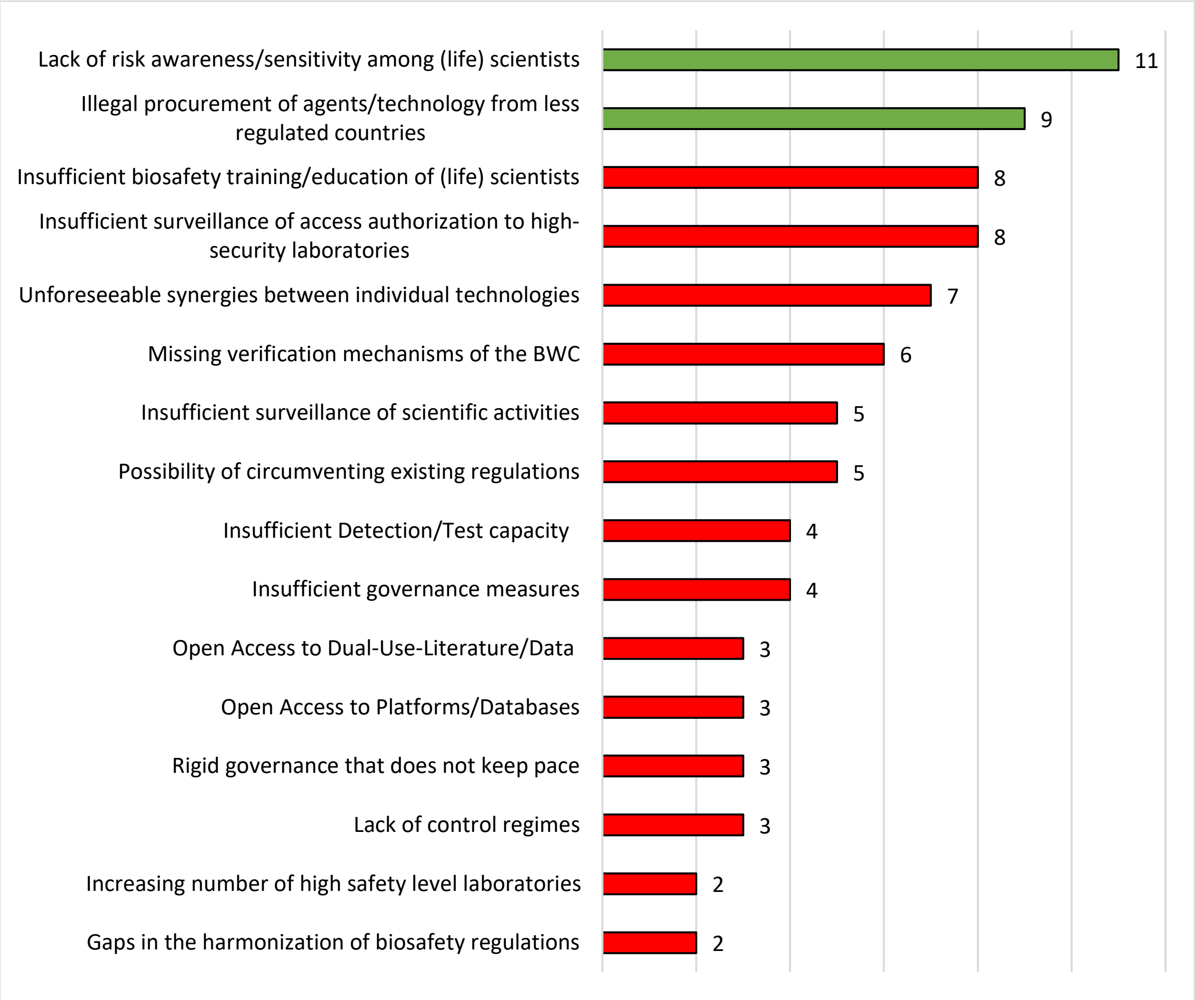

Figure S33: Ranking of responses to question 4.1 of the first survey round. Higher values indicate greater influence. Green bars indicate consensus. Red bars indicate dissent.

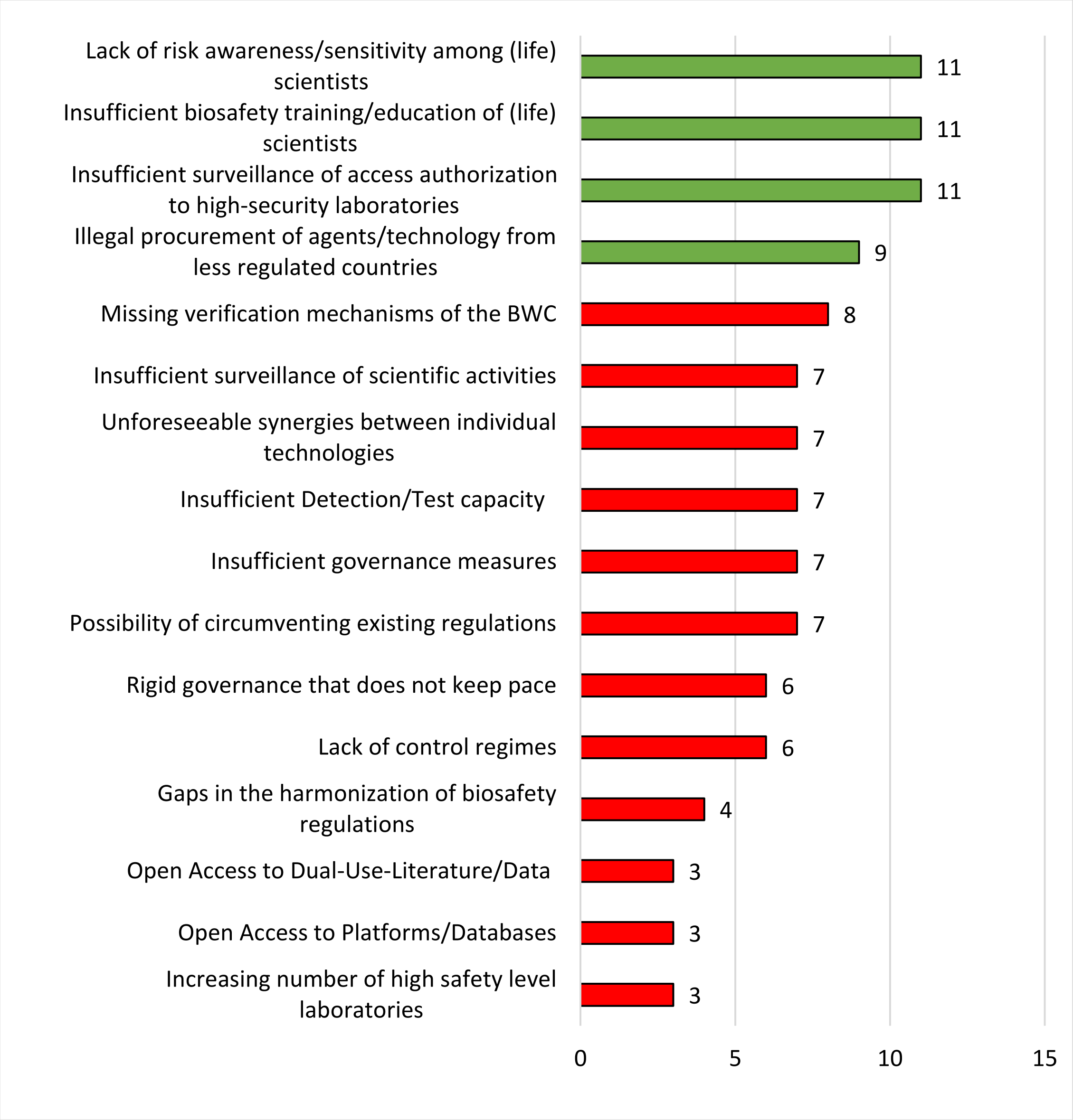

Figure S34: Ranking of responses to question 4.1 of the second survey round. Higher values indicate greater influence. Green bars indicate consensus. Red bars indicate dissent.

5. Implications for biosecurity

5.1 How do you assess the potential impact of synthetic biology and new technologies (e.g., gene editing, CRISPR, etc.) on biosecurity?

The results of the first survey round are shown in Figure S35. A strong tendential consensus was observed here. The results of the second round are shown in Figure S36. In the second survey round, at least two experts changed their opinion, but the strong tendential consensus remained.

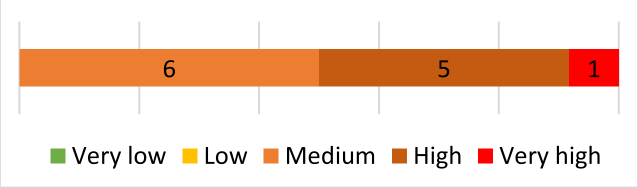

Figure S35: Stacked bar chart showing the responses to question 5.1 of the first survey round.

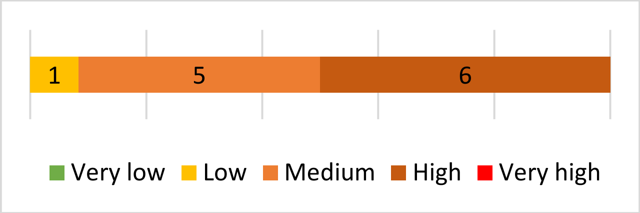

Figure S36: Stacked bar chart showing the responses to question 5.1 of the second survey round.

5.2 Do you think that existing regulations and safeguards will keep pace with the rapid advances in synthetic biology?

The results of the first survey round are shown in Figure S37. The experts voted “No” by a ratio of 5:7. The results of the second survey round are shown in Figure S38. This time, a consensus was reached in the answer “No.”

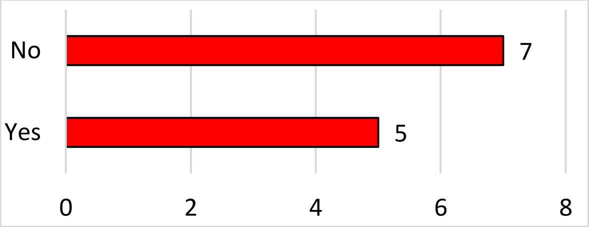

Figure S37: Bar chart showing the responses to question 5.2 of the first survey round. Red bars indicate dissent.

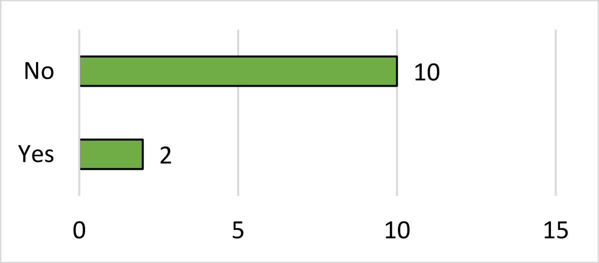

Figure S38: Bar chart showing the responses to question 5.2 of the second survey round. Green bars indicate consensus.

5.3 How do you rate the scientific community's ability to early on identify the scientific advances and driving forces that could facilitate the development of synthetic biological warfare agents and weapons?

The results of the first survey round are presented in Figure S39. A weak tendential consensus was already apparent. The results of the second survey round are shown in Figure S40. There, the weak tendential consensus has shifted to strong, as at least three experts have changed their minds.

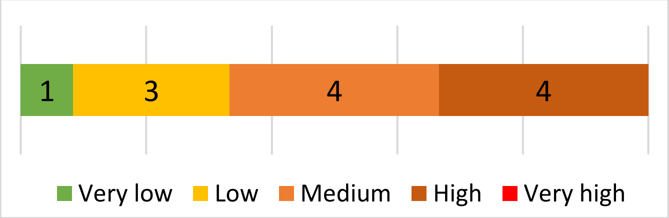

Figure S39: Stacked bar chart showing the responses to question 5.3 of the first survey round.

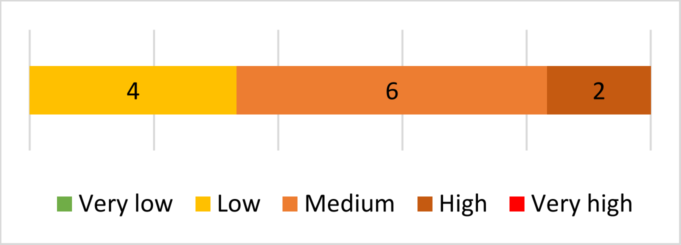

Figure S40: Stacked bar chart showing the responses to question 5.3 of the second survey round.

5.4 How do you assess the ability of states to detect the development of synthetic biological warfare agents and weapons and to respond appropriately?

The results of the first survey round are presented in Figure S41, while the results of the second survey round are shown in Figure S42. In both rounds, a strong tendential consensus was evident, which was further strengthened by one vote in the second round.

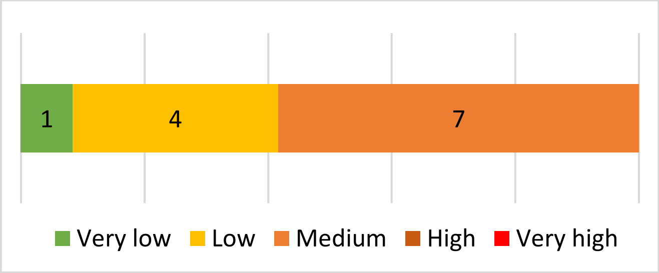

Figure S41: Stacked bar chart showing the responses to question 5.4 of the first survey round.

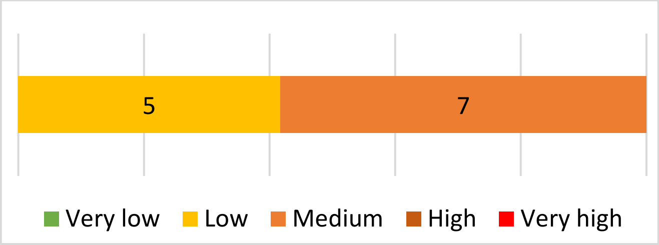

Figure S42: Stacked bar chart showing the responses to question 5.4 of the second survey round.

5.5 Are specific ethical guidelines necessary in research to prevent misuse for biological weapons purposes?

The results of this question are shown in Figure S43. Here, a 10:2 consensus in favor of "yes" was already achieved in the first survey round. As a result, the question was removed from the questionnaire for the second round.

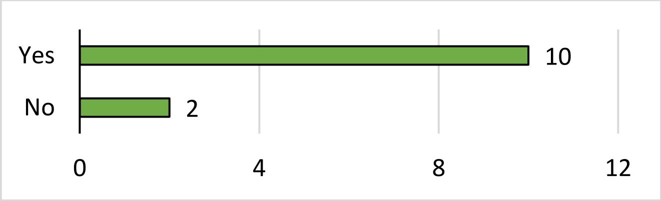

Figure S43: Bar chart showing responses to question 5.5 of the first survey round. Green bars indicate consensus.

6. Government and regulatory measures

6.1 Is current legislation sufficient with regard to biosecurity?

The results of the first survey round are shown in Figure S44. The responses were 2:9 in favor of "yes" for biosafety and unclear concerning biosecurity, with a ratio of 5:6. The missing vote concerning biosafety is due to an expert forgetting to vote on the issue. The results of the second round of surveys are shown in Figure S45. Here, there was a 2:10 consensus in favor of "yes" with regard to biosafety.

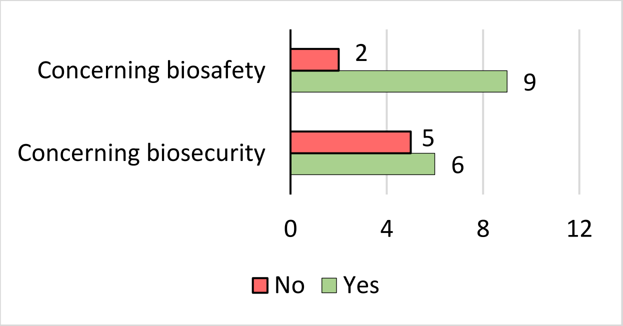

Figure S44: Bar chart showing responses to question 6.1 of the first survey round.

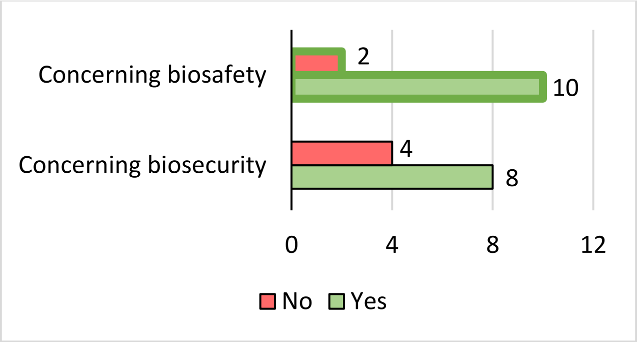

Figure S45: Bar chart showing responses to question 6.1 of the second survey round. Green outlines indicate consensus.

6.2 Do you think governance measures can be adjusted flexibly and quickly to a rapidly changing scientific and technological landscape to prevent the potential development and use of synthetic biological warfare agents and weapons?

Figure S46 shows the results of the first survey round. Here, there was a clear 6:6 disagreement among the experts. The results of the second survey round are shown in Figure S47. This time, the experts reached a 10:2 consensus in favor of the answer "No."

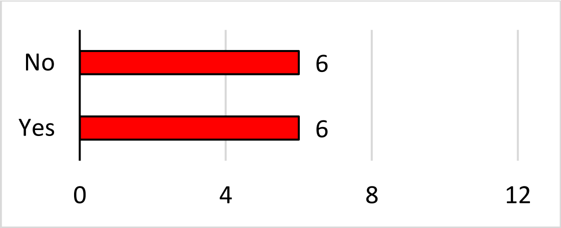

Figure S46: Bar chart showing responses to question 6.2 of the first survey round. Red bars indicate dissent.

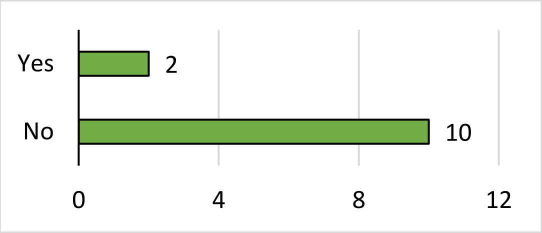

Figure S47: Bar chart showing responses to question 6.2 of the second survey round. Green bars indicate consensus.

6.3 In your opinion, what additional measures are necessary to prevent misuse, given the advances in science and technology?

The results of the first round of surveys are shown in Figure 52. The first survey round did not produce a consensus, but revealed strong tendential consensus regarding eight different items, weak tendential consensus in seven items.

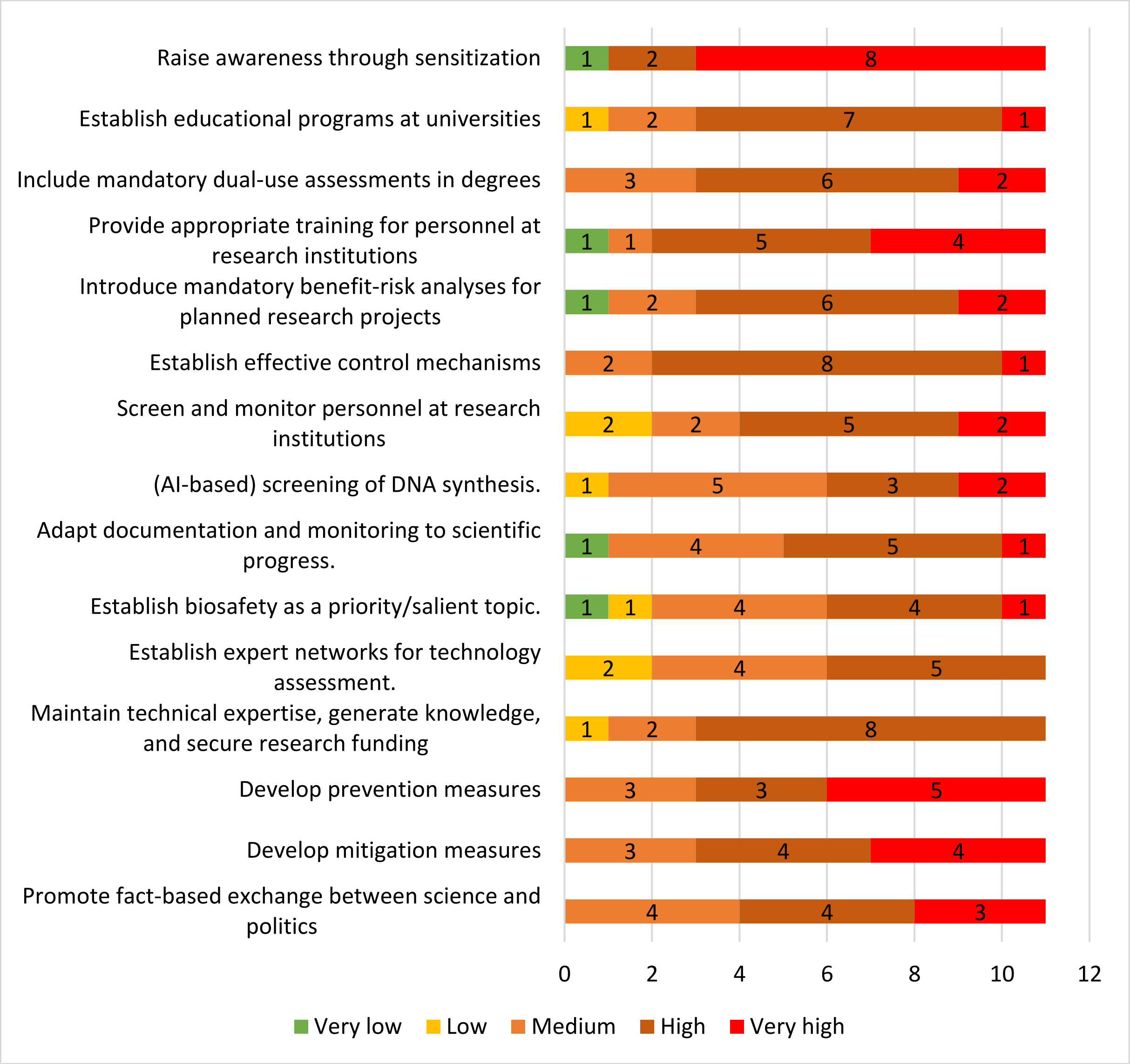

Figure S48: Stacked bar chart of responses to question 6.3 of the first survey round.

The ranking of the assessments is shown in Figure S49. Here, “Raise awareness through sensitization”, “Develop preventive measures”, and “Develop mitigation measures” were rated as the three most

necessary measures to prevent misuse. “Establish biosafety as a priority/salient topic” and “Establish expert networks for technology assessment” were rated the least necessary.

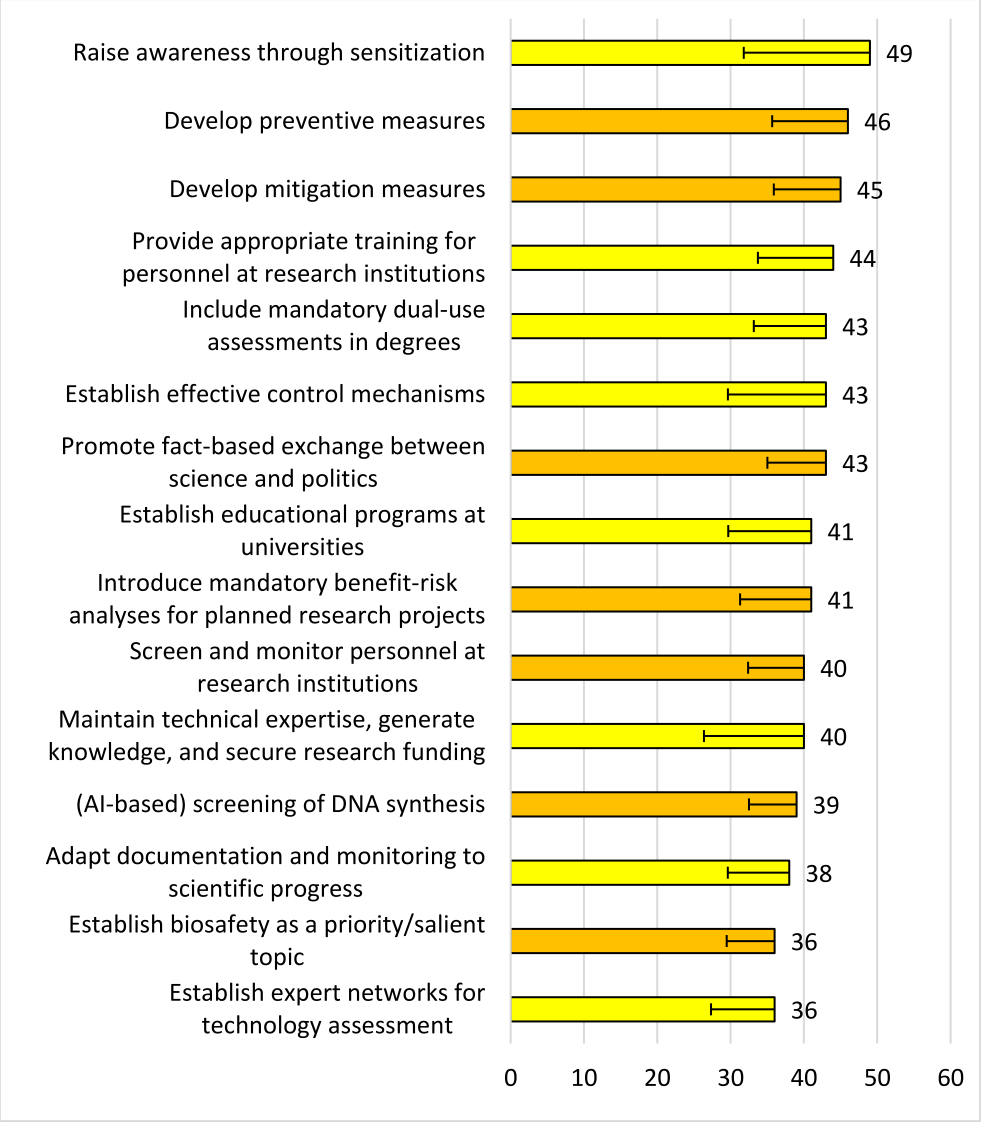

Figure S49: Ranking of responses to question 6.3 from the first round of the survey. Higher values indicate greater influence. Yellow bars indicate strong tendential consensus and orange bars indicate weak tendential consensus. Error bars represent the standard deviation depicted only in one direction for clarity.

Figure S50 shows the results of the second survey round. Here, there was a 10:2 consensus on “Raise awareness through sensitization”. The items “Establish biosafety as a priority/salient topic”, “(AI-based) screening of DNA synthesis”, and “Introduce mandatory benefit-risk analyses for planned research projects” shifted to strong tendential consensus.

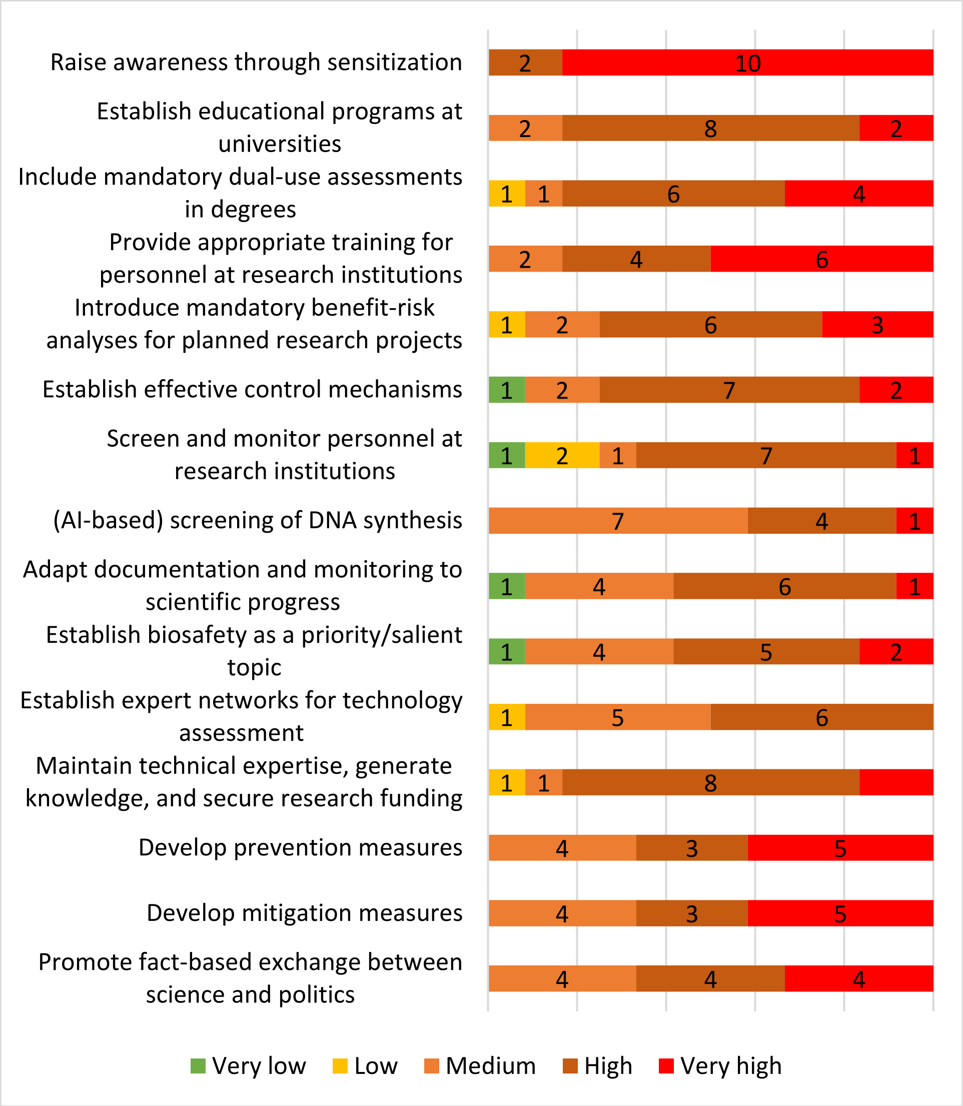

Figure S50: Stacked bar chart of responses to question 6.3 of the second survey round.

The ranking of responses to the second survey is shown in Figure S51. The three measures considered most necessary were still “Raise awareness through sensitization,” “Provide appropriate training for staff at research institutions,” and “Include mandatory dual-use assessments in degrees.” The least necessary measure in this round was “Establish expert networks for technology assessment.”

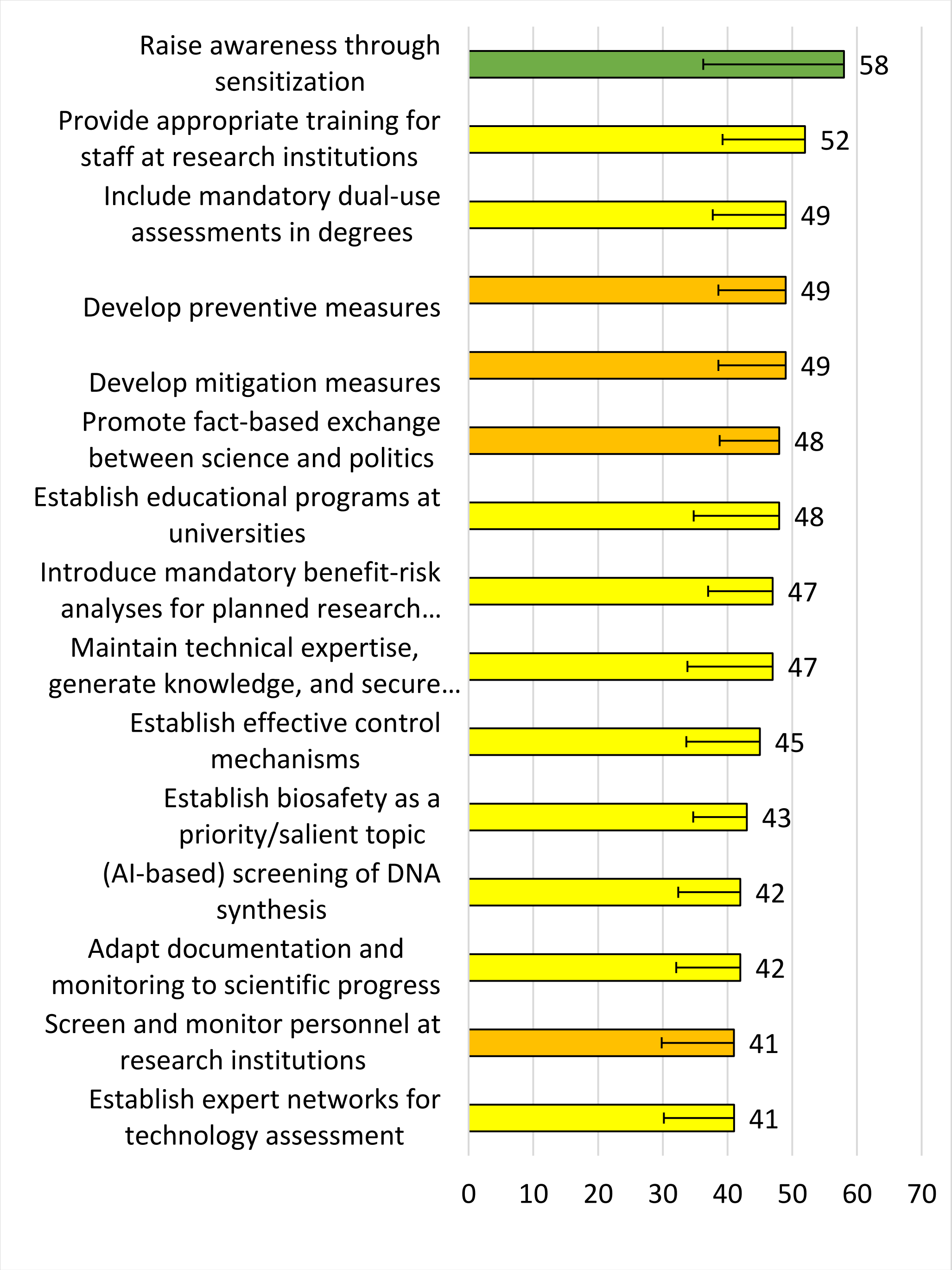

Figure S51: Ranking of responses to question 6.3 of the second survey round. Higher values indicate greater influence. Green bars indicate consensus. Yellow bars indicate strong tendential consensus and orange bars indicate weak tendential consensus. Error bars represent the standard deviation depicted only in one direction for clarity.

6.4 How significant do you consider the impact of the following governance measures mentioned in the interviews in terms of mitigating the threat posed by synthetic biological warfare agents and weapons?

Figure S52 shows the results of the first survey round. Two items, “Increased education and progressive attitude” and “Establishment of interministerial round tables,” achieved a consensus of 9:3. These items were therefore not included in the second survey round. Strong tendential consensus was identified in five items and weak tendential consensus in seven. These can also be tracked in Figure S53, together with the ranking.

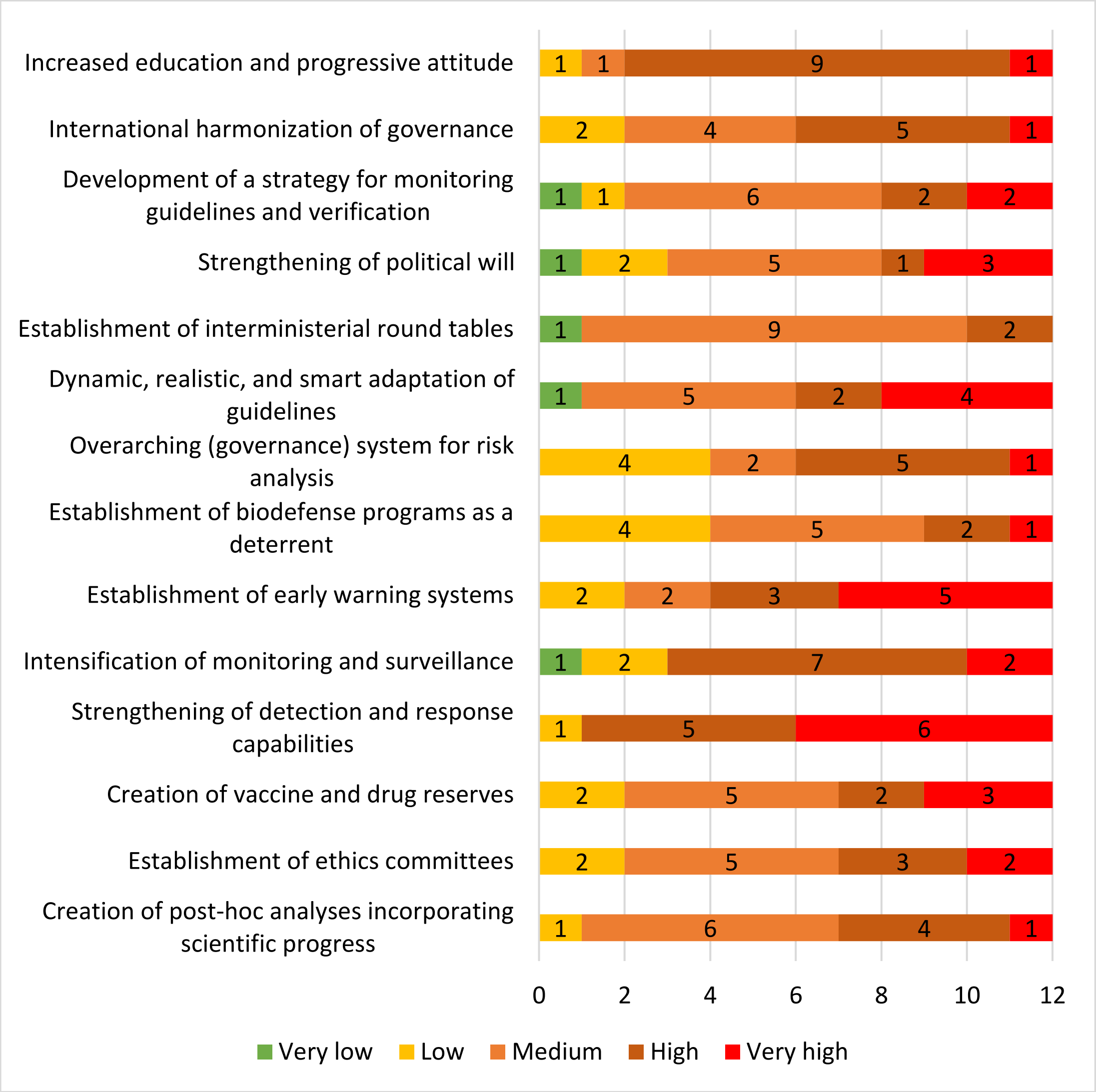

Figure S52: Stacked bar chart of responses to question 6.4 of the first online survey.

The governance measures that experts believe have the greatest impact on mitigating the threat posed by synthetic biological warfare agents are “Strengthen detection and response capabilities”, “Establishment of early warning systems”, and “Increased education and a progressive stance.” The results of the second round of surveys are shown in Figure S54.

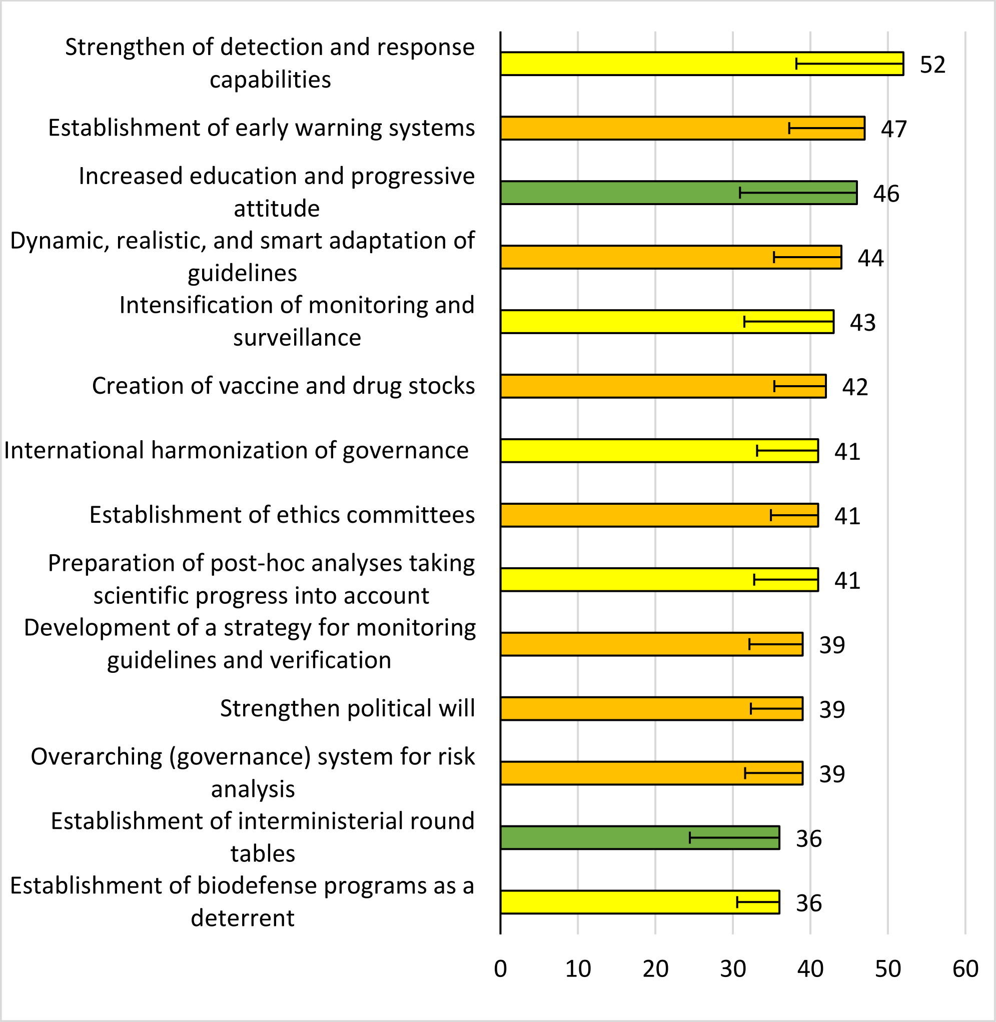

Figure S53: Ranking of responses to question 6.4 of the first survey round. Higher values indicate greater influence. Green bars indicate consensus. Yellow bars indicate strong tendential consensus and orange bars indicate weak tendential consensus. Error bars represent the standard deviation depicted only in one direction for clarity.

No further consensus was reached in the second round, but all remaining items showed tendential consensus, with only three of them showing weak tendential consensus. The ranking and type of tendential consensus is shown in Figure S55. The three governance measures rated as most important were “Strengthen detection and response capabilities”, “Establishment of early warning systems”, and “Intensification of monitoring and surveillance.” The least important measure was “Establishment of ethics committees.”

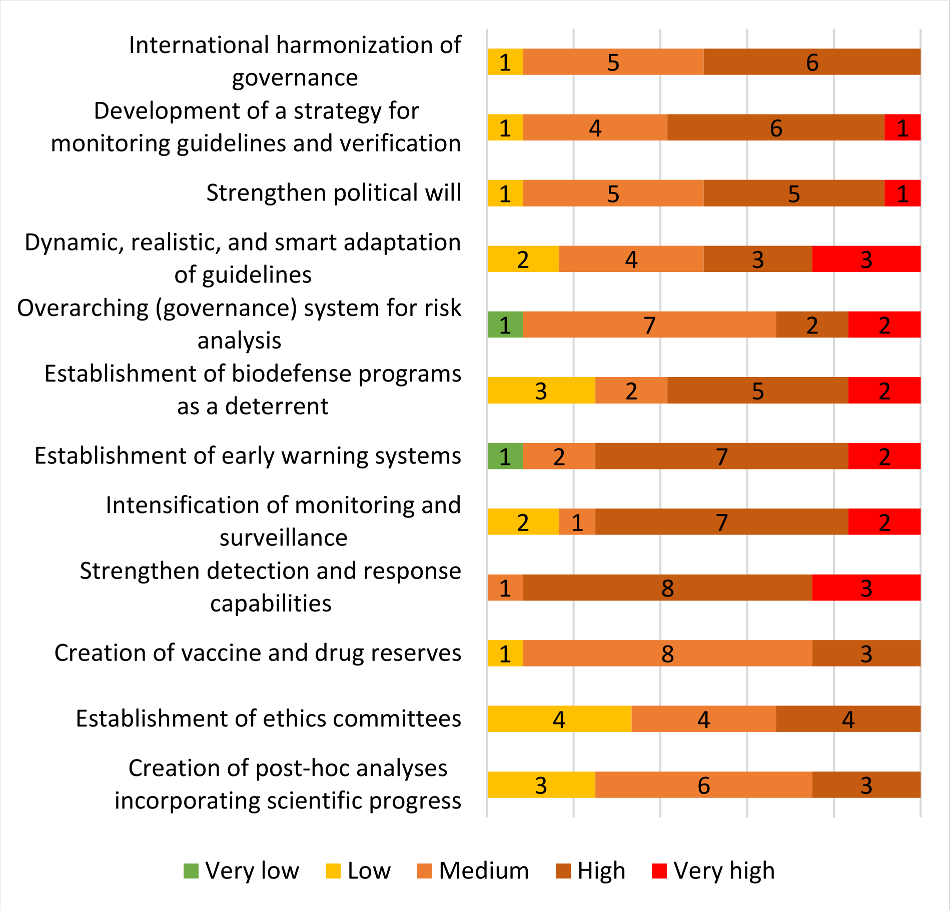

Figure S54: Stacked bar chart of responses to question 6.4 of the second survey round.

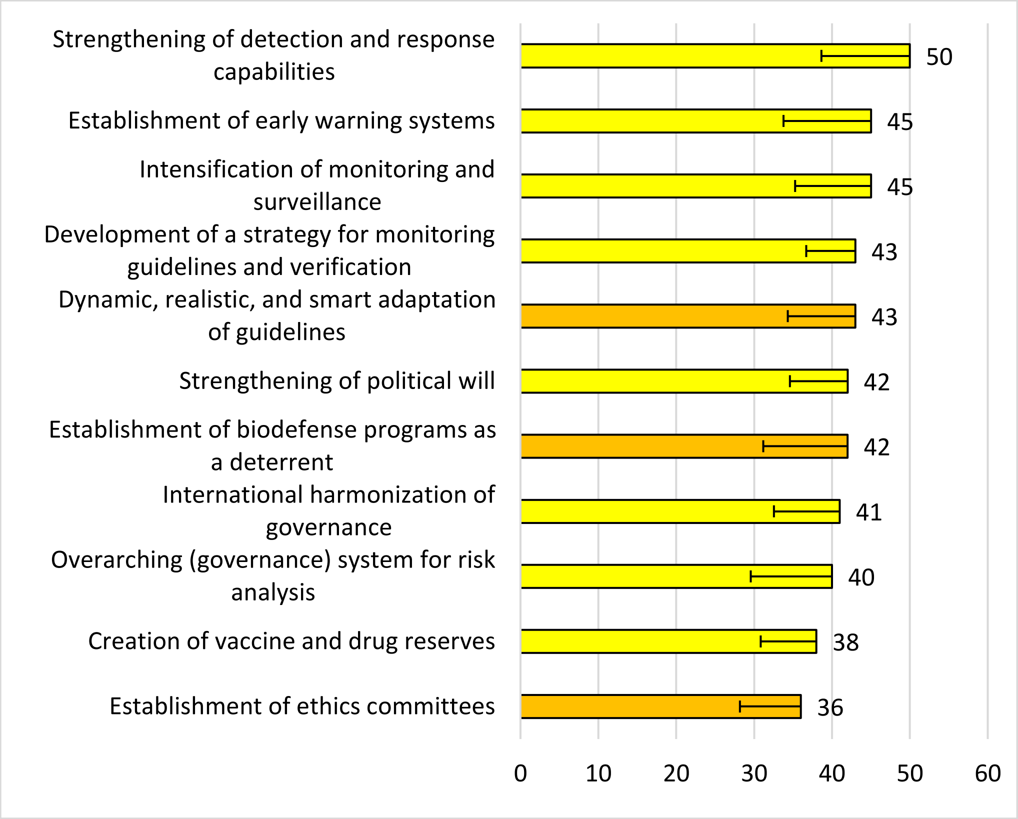

Figure S55: Ranking of responses to question 6.4 of the second survey round. Higher values indicate greater influence. Yellow bars indicate strong tendential consensus and orange bars indicate weak tendential consensus. Error bars represent the standard deviation depicted only in one direction for clarity.

The three governance measures rated as most important were “Strengthening detection and response capabilities”, “Establishment of early warning systems”, and “Intensification of monitoring and surveillance.” The least important measure was “Establishing ethics committees.”

7. Public awareness and education

7.1 How can awareness of biological risks be raised? Please rank the proposed measures in order of importance (1 = most important, 7 = least important).

The distribution of votes in this ranking from the first round of surveys is shown in Figure S56. Apart from dissent in the items “Avoid unnecessary bureaucracy in the implementation of regulations” Use communication experts as mediators (science/society/politics)”, and “Create incentives“ all other items resulted in weak tendential consensus.

The resulting ranking is shown in Figure S57. The three most important items were rated as “Communicate clearly and comprehensibly”, “Communicate directly and openly or transparently”, and “Create incentives”, while “Avoid unnecessary bureaucracy in the implementation of regulations” received the fewest votes.

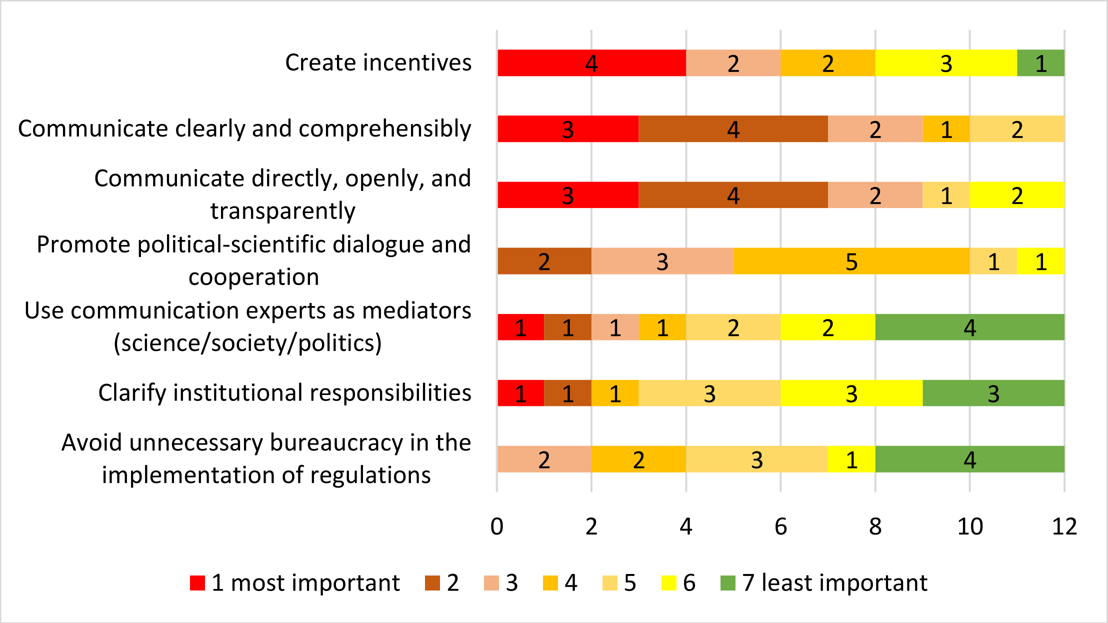

Figure S56: Stacked bar chart of responses to question 7.1 of the first survey round.

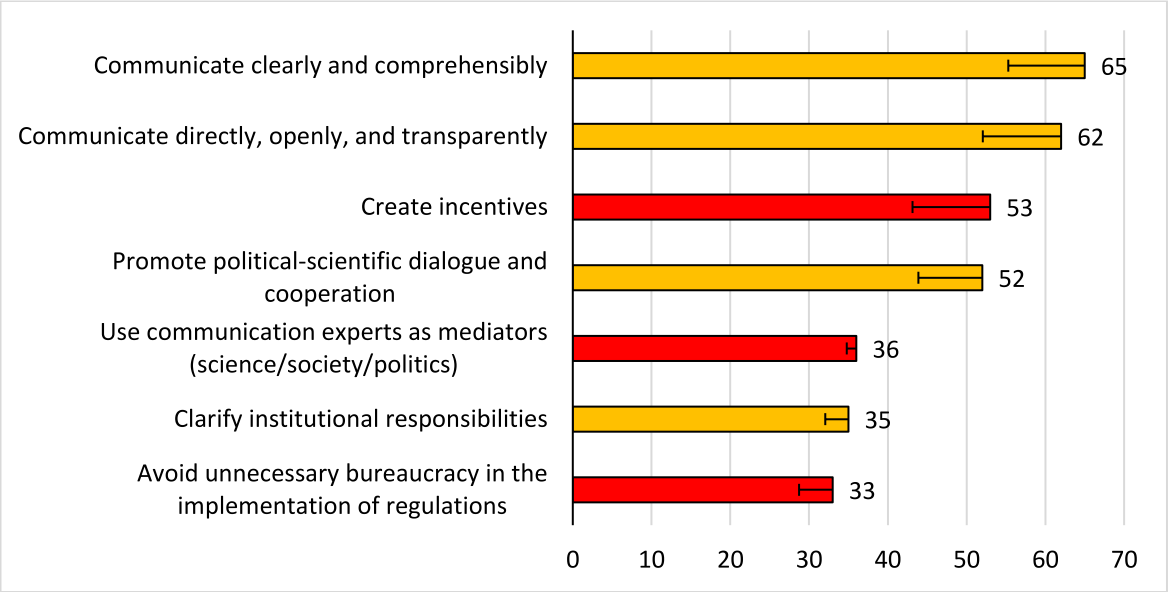

Figure S57: Ranking of responses to question 6.4 of the first survey round. Higher values indicate greater importance. Orange bars indicate weak tendential consensus. Red bars indicate dissent. Error bars represent the standard deviation depicted only in one direction for clarity.

The distribution of votes in the second survey round is shown in Figure S58. Here, there was weak tendential consensus in “Create incentives” and strong tendential consensus in “Communicate directly, openly, and transparently.” All other items showed dissent. The ranking of the second round of the survey is shown in Figure S59 The items “Create incentives”, “Communicate clearly and comprehensibly”, and “Clarify institutional responsibilities” were rated as the three most important. The least important item was “Use communication experts as mediators (science/society/politics)”.

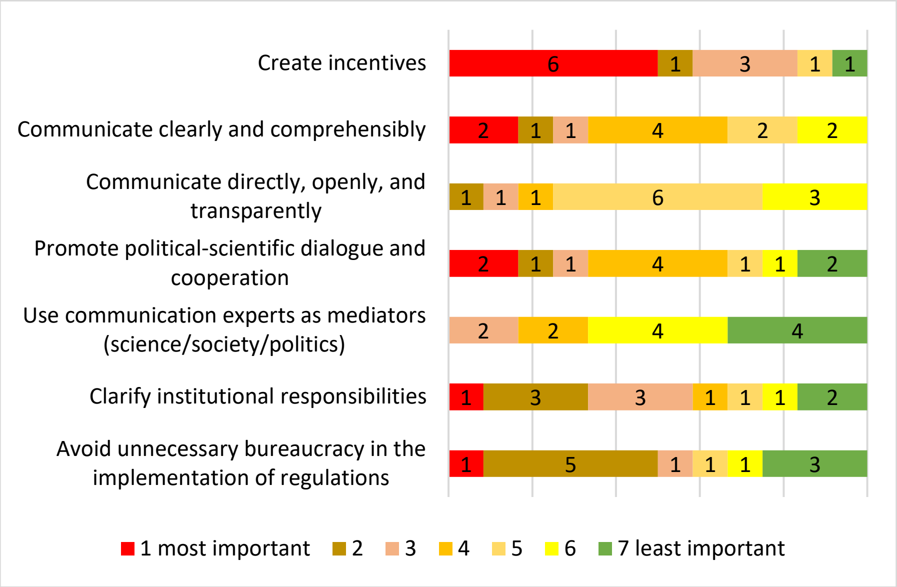

Figure S58: Stacked bar chart showing the responses to question 7.1 of the second survey round.

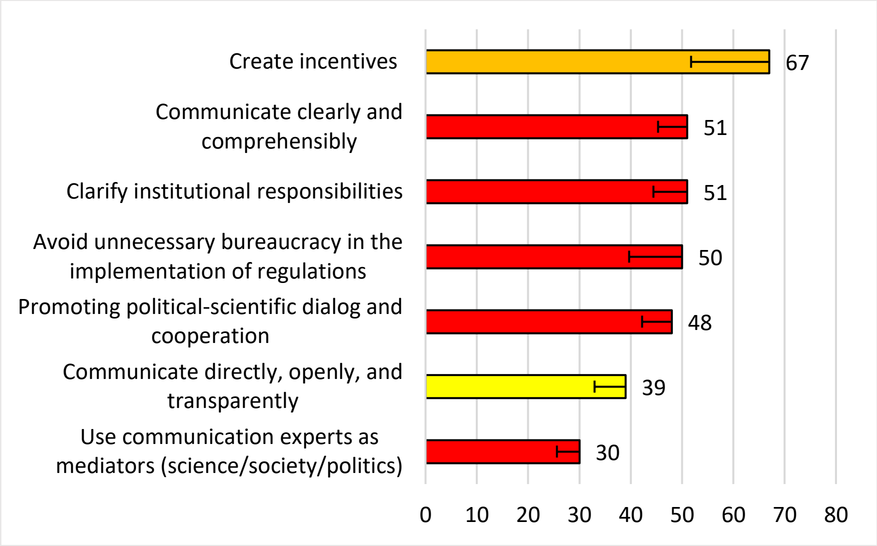

Figure S59: Ranking of responses to question 7.1 of the second survey round. Higher values indicate greater importance. Yellow bars indicate strong tendential consensus and orange bars indicate weak tendential consensus. Red bars indicate dissent. Error bars represent the standard deviation depicted only in one direction for clarity.

7.2 Should more educational programs be implemented to raise awareness of dual use among young scientists?

The results are shown in Figure S60. There was an 11:1 consensus that more educational programs should be conducted for scientists in order to promote greater awareness of dual use. The majority of experts voted “Yes, but participation should be mandatory.” Accordingly, this question was removed from the questionnaire for the second round of the survey.

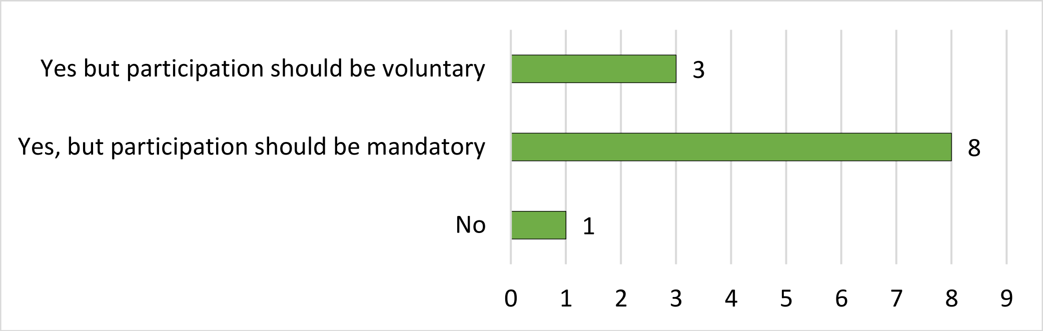

Figure S60: Bar chart showing the responses to question 7.2 of the first survey round.

8. Future-oriented action

8.1 How effective do you think the following approaches by governments and international organizations are in improving global biosecurity?

Figure S61 shows the results of the first survey round. Strong tendential consensus was observed in most items. Weak tendential consensus was observed in four items. Figure S62 shows the ranking of the approaches. The three approaches rated as most effective were “Raise awareness/promote sensitization/conduct training”, “Strengthen preparedness and early detection”, and “Develop mitigation strategies”. “Strive for a just world as a basic prerequisite” was rated as the least effective.

Figure S63 shows the results of the second survey round. Here, there was consensus on two items: “Strengthen the Biological Weapons Convention” and “Promote non-proliferation.” There was weak tendential consensus in three items, while strong tendential consensus was observed in all other items.

Figure S64 shows the ranking of the responses. The three approaches rated as most effective merely changed in order in the second round, with “Develop mitigation strategies” now in second place. “Evaluate and regulate biological design tools” was rated last.

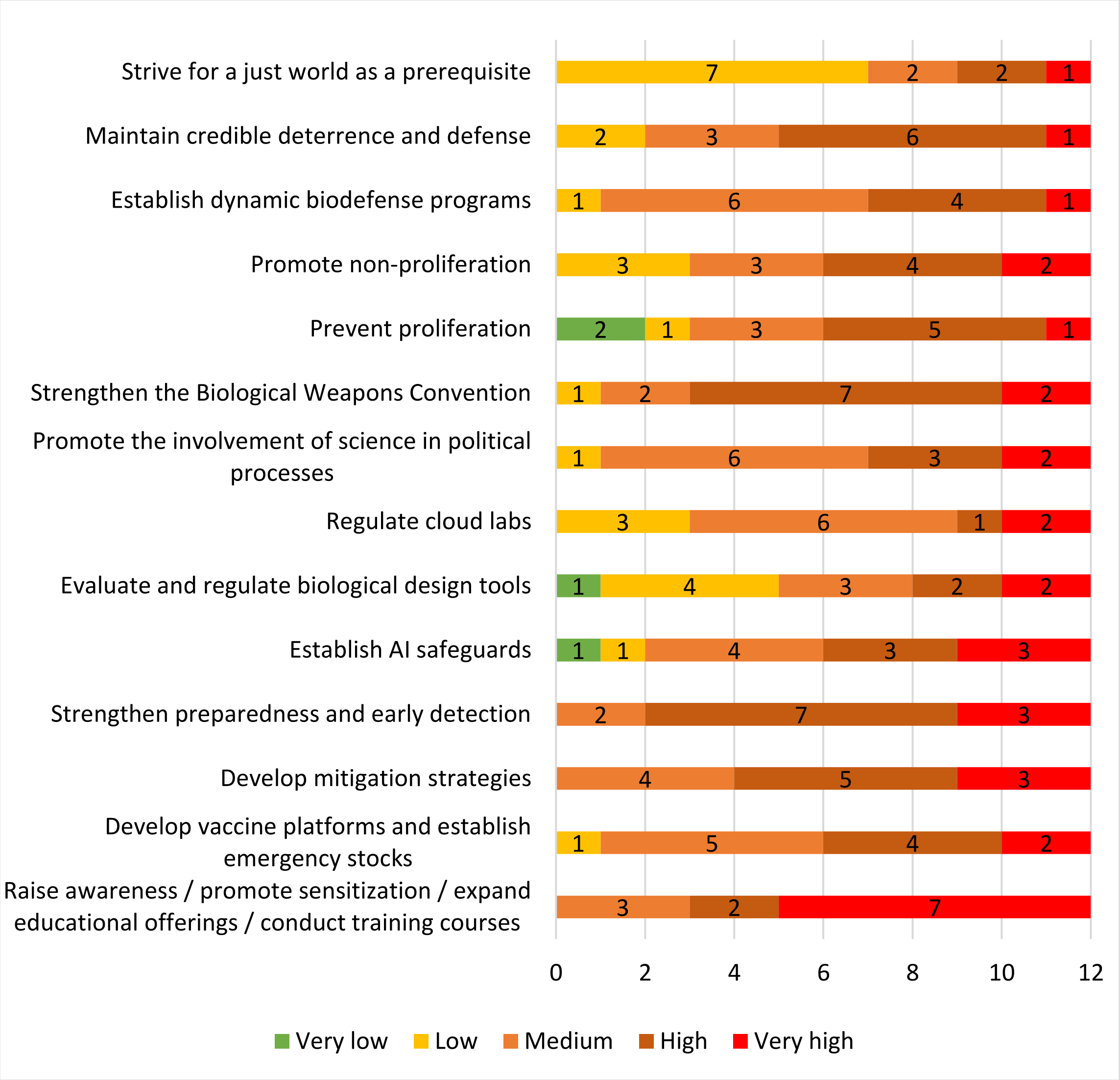

Figure S61: Stacked bar chart of responses to question 8.1 of the first survey round.

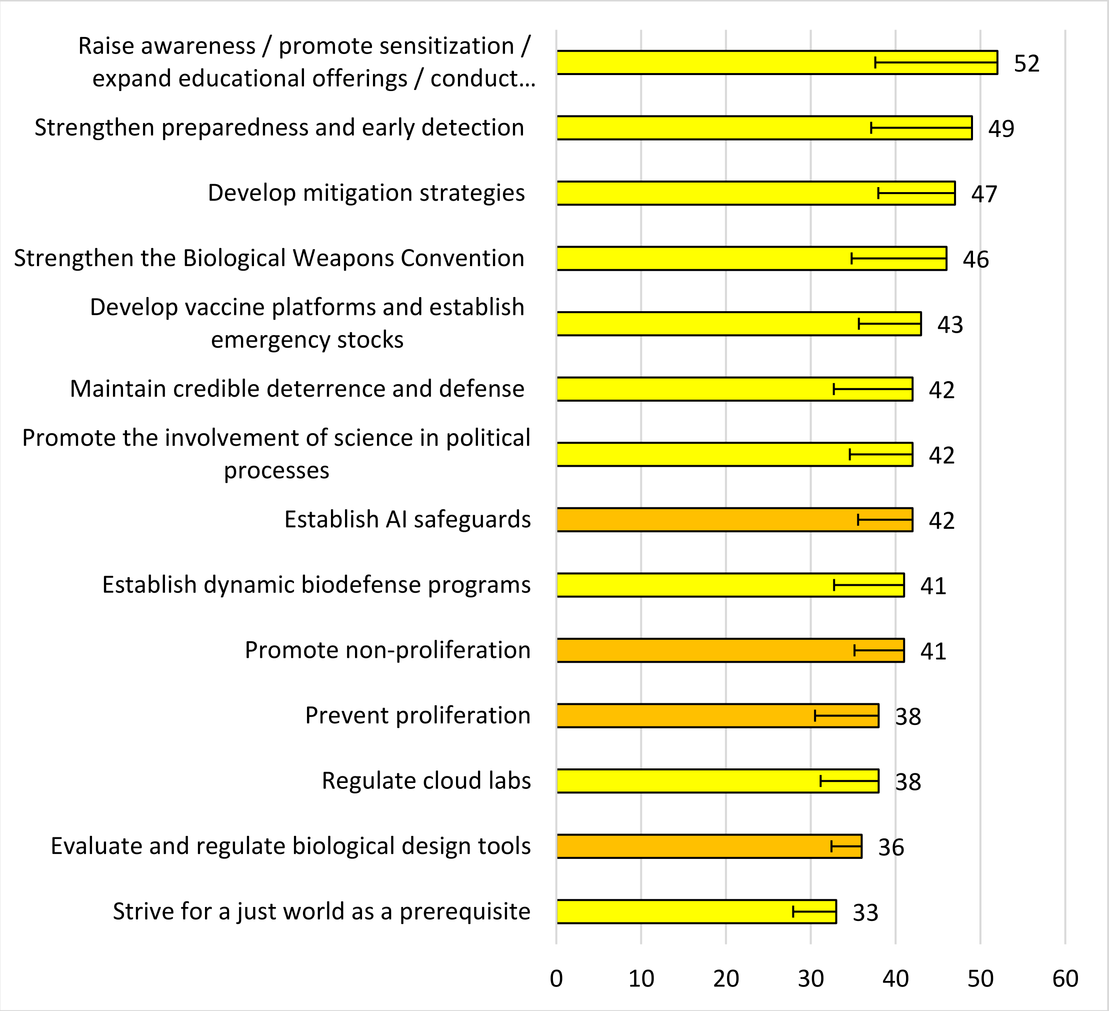

Figure S62: Ranking of responses to question 8.1 of the first survey round. Higher values indicate greater effectiveness. Yellow bars indicate strong tendential consensus and orange bars indicate weak tendential consensus. Error bars represent the standard deviation depicted only in one direction for clarity.

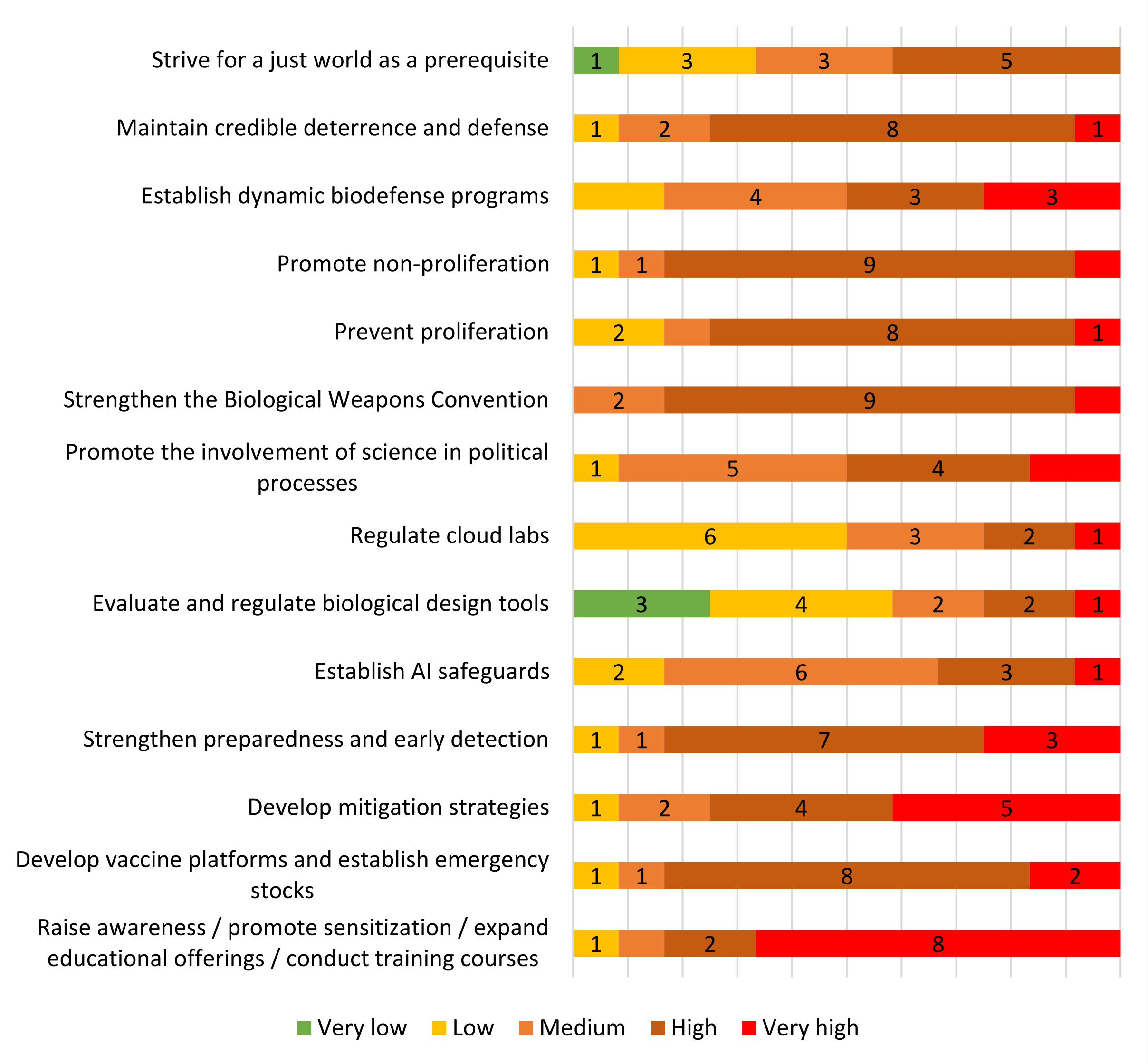

Figure S63: Stacked bar chart of responses to question 8.1 of the second survey round.

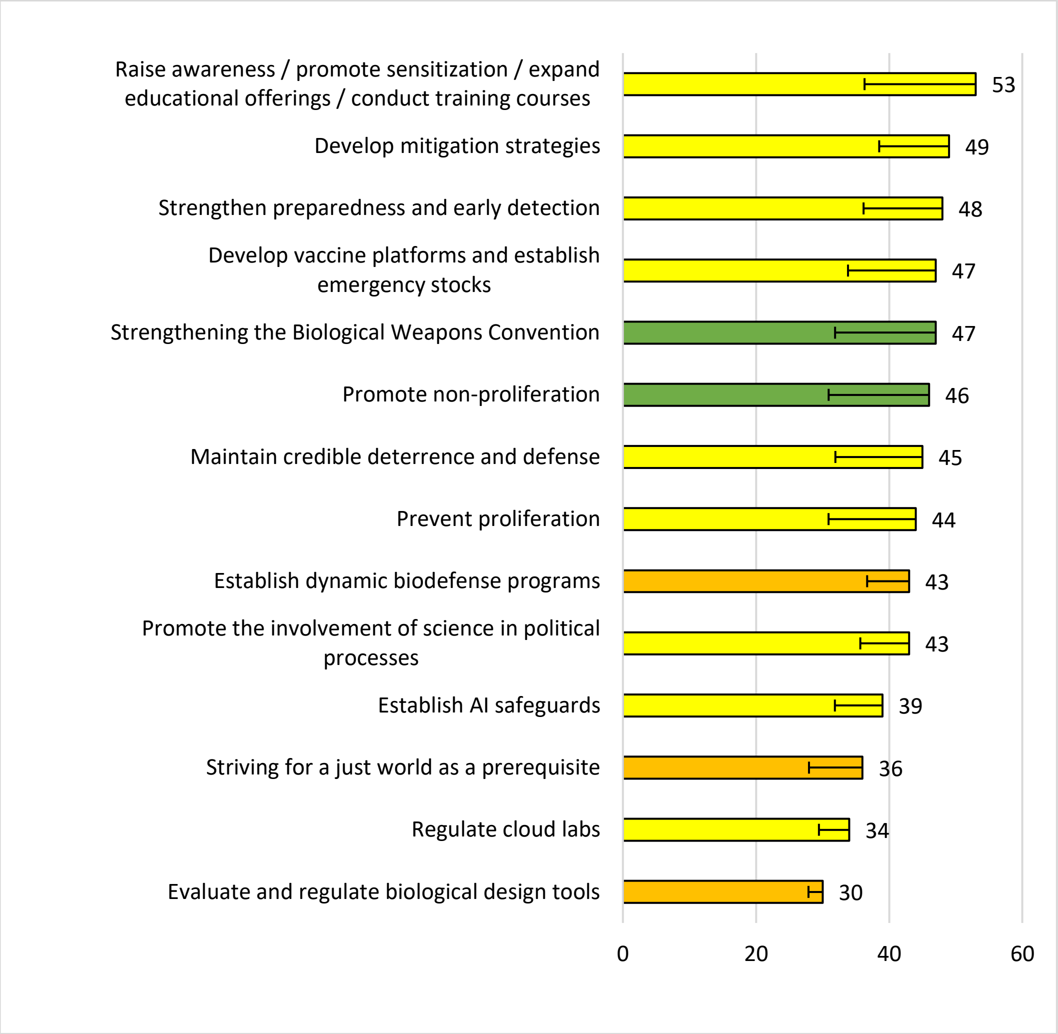

Figure S64: Ranking of responses to question 8.1 of the second survey round. Higher values indicate greater effectiveness. Green bars indicate consensus. Yellow bars indicate strong tendential consensus and orange bars indicate weak tendential consensus. Error bars represent the standard deviation depicted only in one direction for clarity.

The results of the first two survey rounds, like mean values, standard deviations and rankings are also listed in the subsequent Table 1. The comprehensive data and can be found in the Delphi Evaluation Excel file.

Table 1: Numerical data of the first two survey rounds.

		1st Round			2nd Round		
	Item	Mean	SD	Ranking	Mean	SD	Ranking
**1.**							
1.1.							
	Synthetic Biowarfare Agents	1.833	2.082	22	-	-	-
	Conventional Biowarfare Agents	2.083	4.726	25	-	-	-
	Biosecurity	2.500	9.165	30	-	-	-
	Biosafety	2.583	10.504	31	-	-	-
**2.**							
2.1.		**Kendalls W:**		**0.289**	**Kendalls W:**		**0.193**
	Intentional release of a genetically modified pathogen	1.833	12.903	22	2.583	8.012	31
	Intentional release of a natural pathogen	3.167	7.823	38	3.500	7.797	42
	Unintentional release of a genetically modified pathogen	2.667	7.874	32	3.250	7.155	39
	Unintentional release of a pathogen	3.167	5.404	38	3.250	10.257	39
	Naturally occurring pathogens	4.167	3.647	50	4.500	19.409	54
2.4							
	Geopolitical developments and tensions	3.583	9.737	43	3.583	7.335	43
	Existing or increasing ideological motivation	2.917	3.162	35	3.000	9.960	36
	Few or inadequate control options	3.083	5.727	37	3.583	9.317	43
	Increased accessibility for a wider audience	2.500	6.000	30	2.500	5.099	30
	Declining tacit knowledge requirements	2.417	7.014	29	3.167	8.019	38
	Open Access data/databases	2.917	8.000	35	2.583	8.438	31
	Simplified misuse of technology and science	2.583	5.933	31	3.333	5.099	40
	Lower costs of technologies	3.167	5.030	38	3.667	8.349	44
	Greater availability of technologies	3.750	7.483	45	4.000	9.915	48
	Technological and scientific progress	3.583	9.607	43	4.417	16.846	53
**3.**							
3.1							
	Greater availability	3.333	7.106	40	3.083	7.797	37
	Process acceleration through automation	3.250	7.190	39	2.750	6.542	33
	Accelerated development	3.333	11.314	40	3.417	10.109	41
	Increased accessibility for a broader audience	2.667	6.107	32	2.667	5.505	32
	Simplified handling of technologies	3.583	7.861	43	3.500	9.290	42
	Decreasing costs	3.167	4.561	38	3.417	7.887	41
3.2							
	Synthetic bioregulators	3.333	5.099	40	3.333	5.099	40
	HEGAA (Insect Allies)	2.917	6.856	35	2.500	6.364	30
	Gene drives	2.667	6.066	32	2.500	8.485	30
	Genetically modified bacteriophages	2.750	5.814	33	2.333	5.857	28
	De novo synthesis of nucleic acids	3.750	7.483	45	3.167	5.941	38
	Gain-of-function research	3.333	7.450	40	3.500	11.194	42
	CRISPR/Cas	3.500	6.387	42	3.083	6.618	37
	Modular biology (kits for everyone/everything)	2.833	6.535	34	3.083	5.079	37
	Bench-top DNA synthesizers	3.833	8.468	46	3.500	9.607	42
	Next-generation sequencing	2.833	6.535	34	2.833	6.611	34
	Targeted drug delivery	3.000	5.975	36	3.167	8.019	38
	Artificial organelles	2.333	7.403	28	-	-	-
3.3							
	Microelectronics	2.333	5.177	28	2.000	5.357	24
	Additive Manufacturing	2.667	6.107	32	2.000	5.215	24
	Quantum Computing	2.500	6.364	30	2.083	3.000	25
	Automatization and Robotics	2.917	6.856	35	2.583	5.848	31
	Cloud Computing	2.833	6.261	34	2.500	6.745	30
	Cloud Labs	2.667	5.505	32	2.333	4.775	28
	Shared laboratory spaces	1.917	5.814	23	1.917	4.450	23
	AI-based Large Language Models	2.917	8.602	35	2.833	6.535	34
	AI-modified protein-design, e.g. AlphaFold	3.583	7.537	43	3.250	7.190	39
	Synthetic Chemistry and machine-learning for reaction conditions	3.333	10.198	40	3.083	8.473	37
3.4							
	Use of synthetic biowarfare agents increased by converging and emerging technologies	2.000	6.083	24	1.833	2.082	22
	Use of synthetic biocidal agents developed through synthetic biology	1.917	5.686	23	2.083	6.028	25
	Use of conventional bioweapons intensified by converging and emerging technologies	2.333	7.371	28	2.083	8.737	25
	Use of conventional bioweapons	2.167	5.774	26	2.500	10.149	30
**4.**							
4.1							
	Possibility of circumventing existing regulations	0.417	1.414	5	0.583	2.729	7
	Lack of control regimes	0.250	4.243	3	0.500	2.825	6
	Lack of verification instruments under the BWC	0.500	0.000	6	0.667	2.921	8
	Incomplete harmonization of biosafety regulations	0.167	5.657	2	0.333	3.023	4
	Inadequate governance measures	0.333	2.828	4	0.583	3.029	7
	Rigid, outdated governance	0.250	4.243	3	0.500	3.177	6
	Inadequate detection/testing capacities	0.333	2.828	4	0.583	3.327	7
	Unpredictable synergies between individual technologies	0.583	1.414	7	0.583	3.528	7
	Insufficient monitoring of access authorization to high-security laboratories	0.667	2.828	8	0.917	4.275	11
	Increasing number of high-security laboratories	0.167	5.657	2	0.250	4.190	3
	Open access to platforms/databases	0.250	4.243	3	0.250	4.598	3
	Open access to dual-use literature/data	0.250	4.243	3	0.250	4.845	3
	Inadequate monitoring of scientific activities	0.417	1.414	5	0.583	5.125	7
	Inadequate biosafety training/education of (life) scientists	0.667	2.828	8	0.917	5.718	11
	Lack of risk awareness/sensitivity among (life) scientists	0.917	7.071	11	-	-	-
	Illegal procurement of agents/technology from less regulated countries	0.750	4.243	9	-	-	-

		1st Round			2nd Round		
	Item	Mean	SD	Ranking	Mean	SD	Ranking
5.							
5.1							
	How do you assess the potential impact of synthetic biology and new technologies (e.g. gene editing, CRISPR,...) on biosafety?	3.909	3.202	43 of 60	3.417	10.826	41 of 60
5.3							
	How do you rate the scientific community's ability to early on identify the scientific advances and driving forces that could facilitate the development of synthetic biological warfare agents and weapons?	3.182	6.928	35 of 60	2.833	7.430	34 of 60
5.4							
	How high do you rate the ability of states to recognize and respond appropriately to the development of synthetic biowarfare agents and weapons?	2.727	9.028	30 of 60	2.583	9.3381	31 of 60
6.							
6.3							
	Promote evidence-based exchange between science and politics	3.909	7.987	43	4.000	9.209	48
	Develop mitigation measures	6.250	9.110	45	4.083	10.402	49
	Develop prevention measures	5.250	10.330	46	4.083	10.402	49
	Maintain expertise, generate knowledge and secure research funding	10.000	13.638	40	3.917	13.183	47
	Establish biosafety expert networks on technology assessment in networks on technology assessment	9.000	8.672	36	3.417	10.826	41
	Establish biosafety as a priority/salient topic	7.750	6.535	36	3.583	8.295	43
	Adapt documentation and monitoring to scientific progress	8.250	8.385	38	3.500	9.915	42
	(AI-based) screening of DNA synthesis	7.250	6.496	39	3.500	9.607	42
	Screen and monitor staff at research institutions	7.500	7.616	40	3.417	11.167	41
	Establish effective control mechanisms	9.500	13.372	43	3.750	11.358	45
	Introduce mandatory risk-benefit analyses for planned research projects	7.750	9.706	41	3.917	9.990	47
	Provide appropriate training for staff at research institutions	6.000	10.281	44	4.333	12.759	52
	Incorporate mandatory dual-use assessments into degrees	8.250	9.839	43	4.083	11.278	49
	Establish educational opportunities at universities	9.000	11.323	41	4.000	13.221	48
	Raise awareness through sensitization	2.250	17.210	49	4.833	21.744	58
6.4							
	Preparation of post-hoc analyses taking into account scientific progress	3.417	8.258	41	-	-	-
	Establishment of ethics committees	3.417	6.099	41	-	-	-
	Creation of vaccine and drug stockpiles	3.500	6.656	42	3.167	10.431	38
	Strengthening of detection and response capability	4.333	13.813	52	4.167	13.766	50
	Tightening of monitoring and surveillance	3.583	11.524	43	3.75	11.225	45
	Establishment of early warning systems	3.917	9.737	47	3.75	11.358	45
	Establishment of biodefence programmes for deterrence	3.000	5.450	36	3.5	7.403	42
	Overarching (governance) system for risk analysis	3.250	7.430	39	3.333	8.456	40
	Dynamic, realistic and smart adaptation of guidelines	3.667	8.701	44	3.583	6.309	43
	Establishment of interministerial round tables	3.000	11.563	36	-	-	-
	Strengthening of political will	3.250	6.686	39	3.5	8.678	42
	Development of a strategy for monitoring guidelines and verification	3.250	6.870	39	3.583	9.737	43
	International harmonization of governance	3.417	7.887	41	3.417	10.826	41
	Increased education and progressive attitude	3.833	15.090	46			
7.							
7.1		**Kendalls W**		**0.152**	**Kendalls W**		**0.194**
	Avoid unnecessary bureaucracy in the implementation of regulations	2.750	4.271	33	4.167	10.32	50
	Clarify institutional responsibilities	2.917	2.944	35	4.250	6.576	51
	Use communication experts as mediators (science/society/policy)	3.000	1.215	36	2.500	4.386	30
	Promote political-scientific dialog and cooperation	4.333	8.121	52	4.000	5.786	48
	Communicate directly and openly or transparently	5.167	9.940	62	-	-	-
	Communicate clearly and comprehensibly	5.417	9.708	65	4.250	5.678	51
	Create incentives (incentivization)	4.417	9.863	53	5.583	15.24	67
8.							
8.1							
	Raise awareness / promote awareness-raising / expand educational opportunities / provide training	4.333	14.398	52	4.417	16.697	53
	Develop vaccine platforms and establish emergency stocks	3.583	7.335	43	3.917	13.183	47
	Develop mitigation strategies	3.917	9.044	47	4.083	10.498	49
	Strengthen preparedness and early detection	4.083	11.883	49	4.000	11.845	48
	Establish AI safeguards	3.500	6.427	42	3.250	7.155	39
	Evaluate and regulate biological design tools	3.000	3.564	36	2.500	2.121	30
	Regulate cloud labs	3.167	6.841	38	2.833	4.550	34
	Promote the integration of science into political processes	3.500	7.403	42	3.583	7.335	43
	Strengthen the Biological Weapons Convention	3.833	11.189	46	3.917	15.126	47
	Prevent proliferation	3.167	7.503	38	3.667	13.103	44
	Promote non-proliferation	3.417	5.848	41	3.833	15.090	46
	Establish dynamic biodefence programmes	3.417	8.258	41	3.583	6.309	43
	Maintain credible deterrence and defence	3.500	9.290	42	3.750	13.077	45
	Aim for a just world as a prerequisite	2.750	5.079	33	3.000	8.044	36

Scenario workshop

In an online group workshop, we presented three bioweapon scenarios (bacteria, bioregulators, gene drive-yeasts) to the expert panel. The information on those scenarios was sent to the experts one week prior to the workshop. The experts had the opportunity to ask questions on the scenarios beforehand. Also, the experts were asked to rank the BW agents presented in the categories of development,

release, human health impact, societal impact, economic impact, environmental impact and mitigation. During the three-hour workshop the experts discussed in breakout groups the following questions:

How could SynBio and converging technologies influence these three scenarios with regard to a) biothreat, b) mitigation, c) potential actors and their motivation.

Subsequently, the experts were asked to brainstorm and sketch-out a fourth scenario on a vial agent.

Information on the specifics of the scenarios is withheld due to information hazard concerns.

Survey workshop

The second workshop of the project was held on May 29, 2024. This workshop focused on the results of the surveys and aimed to inform participants about the results regarding consensus and dissent, and to discuss these points of dissent in more detail. These questions were then put to a vote again. Finally, the recommendations for action that were to emerge from the Delphi process and their addressees were decided upon. The elaborated recommendations formulated in the manuscript resulted from the notes taken during this workshop.

The following comments from the experts regarding the discussion of the results and possible recommendations were made:

•Disagreement may have been caused by a lack of clarity regarding the actors considered (governmental or non-governmental), whether intentional or unintentional release was considered, or the intended target groups for the questions.

• The recommendations should make clear that they relate more to biosecurity than to biosafety.

The following questions were addressed in breakout session:

Discussion of dissenting topics

What is the basis for the dissent regarding the intentional release of a genetically modified pathogen?

- It was noted here that the threat posed by state actors is not acutely perceived.
- Another possible reason cited was that the consequences in the field of GM pathogens are rather uncertain and require more work.
- Likewise, it may be unclear here whether this refers to a natural, accidental, or intentional release.
- Furthermore, the depth of experience of the participants may vary, particularly in their perception of the threat, which can lead to further disagreement.
- The ability to produce genetically modified pathogens is perceived more with state actors.

What is the basis for the dissent regarding biothreats (in general)?

- As before, it was suggested that the disagreement could be due to the breadth of different biothreats. Be they natural biothreats, such as a pandemic, or man-made threats such as unintentional releases, laboratory accidents, or the intentional release of biological warfare agents.
- The latter were considered more relevant to the question.
- Likewise, the probability of occurrence should also be considered for various threats, especially with regard to policy recommendations.
- However, the impacts of threats can at best be estimated with a high degree of variance.

- Individual scientists often lack sufficient awareness of the consequences of their experiments and their dual-use potential.
- Thus, there is heterogeneous awareness in different scientific fields.
- To increase consensus, individual, clearly defined scenarios could be addressed in the questions.
- Policymakers want simple answers, so a caveat should be made that these are based on the available information. This is important for assessing the strength of the recommendation.

What is the basis for the dissent regarding sinking costs?

- Different assumptions regarding the actors; state actors may be less influenced by falling costs.
- A distinction should also be made with non-state actors. State-sponsored non-state actors may consider falling costs to be secondary.

What is the basis for the dissent regarding the establishment of biodefense programs for deterrence?

- The topic is very broad. What would such a program entail, particularly with regard to deterrence? Does it primarily revolve around detection and prevention, stockpiling of PPE, etc.?
- What is meant by deterrence? Because there are effective countermeasures, or more in the direction of deterrence?
- Others understood the term as a nationally coordinated defense strategy, coordinated across multiple ministries with numerous measures, in a layered defense system.
- Deterrence would be understood to open the barrier to an offensive context.
- In NATO, the nuclear umbrella is considered a deterrent against the use of weapons of mass destruction A, B, and C. Accordingly, a biodefense program need not fulfill this role of deterrence. A more sensible question would be about increased mitigation capabilities.
- However, prevention can never cover everything in detail; at best, it can establish general plans.

Policy Recommendations

How can awareness of bio-risks be increased?

- It should be addressed early in the career, i.e., already during studies, starting with master's students, with mandatory courses, in the form of instruction.
- This could be handled through ethics workshops.
- Mandatory events, but not a mandatory exercise that can be checked off.
- Workshops could be offered at institutions, including workshops that focus on networking between institutions.
- It would be sensible to offer appropriate incentives to improve implementation in these institutions.
- Germany is one of the few countries that actually has a genetic engineering law and enforces it, and non-compliance is connected to fines.
- Regarding voluntary committees for ethics in research, there are large universities in Germany that do not yet participate. Accordingly, voluntary participation is mandatory for everyone, from the bottom up and from the top down, i.e., students as well as project leaders. Not necessarily in the form of annual DURC inspections, but as a fixed responsibility.
- Incentives might be possible in the bureaucratic area, or rather, it would be a shame if such measures entailed additional bureaucratic hurdles. However, prior coverage during the studies

could help to ease the management of such bureaucratic requirements regarding applications and administration.

- Most of the measures being discussed here already existed in the context of iGem and are mandatory. It would be interesting to see whether it has become a mandatory exercise; there are the Responsible Research Workshops, but the question would be what benefits it has achieved and what impact it has on the participants. My fear is that the participants are not affected.
- Effective Altruism also flirts with these risks, explicitly to obtain research funding. One conceivable innovation would be to include not only teaching and research for university lecturers, but also communication with stakeholders and engagement with DURC and dialogue events.
- It has been very beneficial for the iGem participants. Especially since they have recently focused more on biosecurity, even if it only reaches a certain number of people.
- And be careful about the differentiation between elective and mandatory. In my experience, students, once introduced to this topic, are very interested. All of the aspects mentioned are important and good.
- As an incentive, the DFG had required a DURC assessment in project proposals. However, this requires an oversight committee, which again places training and teaching at the forefront.
- The National Academy is attempting to establish such a system, but it is proving difficult.
- International collaboration between institutions declines with lower awareness.
- Ethics awareness must be raised, and mandatory ethical considerations and DURC assessments must be conducted for project proposals. Self-initiative to report DURC aspects.

What measures can governments and international organizations take to improve global biosecurity?

- Standards in Europe are different from those in the rest of the world, so training programs would be useful to align skills. Congruent guidelines, for example, on DNA synthesis, would also be desirable.
- Mitigation strategies, whether political or technological, should be categorized. Then you have prevention, detection, and response, and these three areas are needed. Since these are global problems, multilateral cooperation is necessary, which is also supported nationally by security and health experts. However, the categories in this question were very vague and partially overlapping, making evaluation difficult.
- Ethics committees and committees for committees for ethics in research (KEF) are two different things.

References

De Loë, R.C., Melnychuk, N., Murray, D., Plummer, R., 2016. Advancing the state of policy Delphi practice: a systematic review evaluating methodological evolution, innovation, and opportunities. Technol. Forecast. Soc. Change 104, 78–88.

Denscombe, M., 2021. The good research guide: research methods for small-scale social research projects. McGraw-Hill Education (UK).

Turoff, M., 1970. The design of a policy Delphi. Technol. Forecast. Soc. Change 2, 149–171.
